# Mass spectrometry imaging as a potential technique for diagnostic of Huanglongbing disease using fast and simple sample preparation

**DOI:** 10.1038/s41598-020-70385-4

**Published:** 2020-08-10

**Authors:** João Guilherme de Moraes Pontes, Pedro Henrique Vendramini, Laura Soler Fernandes, Fabricio Henrique de Souza, Eduardo Jorge Pilau, Marcos Nogueira Eberlin, Rodrigo Facchini Magnani, Nelson Arno Wulff, Taicia Pacheco Fill

**Affiliations:** 1 Laboratório de Biologia Química Microbiana (LaBioQuiMi), IQ-UNICAMP, Campinas, SP Brazil; 2 ThoMSon Mass Spectrometry Laboratory, IQ-UNICAMP, Campinas, SP Brazil; 3 MackMass Laboratory, School of Engineering - PPGEMN, Mackenzie Presbyterian University, São Paulo, SP Brazil; 4 Programa de Pós-graduação Em Ciências Biológicas, Universidade Estadual de Maringá, Maringá, PR Brazil; 5 Departamento de Química, Laboratório de Biomoléculas E Espectrometria de Massas (LabioMass), Universidade Estadual de Maringá, Maringá, PR Brazil; 6 Departamento de Pesquisa & Desenvolvimento, Fundo de Defesa da Citricultura (FUNDECITRUS), Araraquara, SP Brazil

**Keywords:** Chemical biology, Natural products

## Abstract

Huanglongbing (HLB) is a disease of worldwide incidence that affects orange trees, among other commercial varieties, implicating in great losses to the citrus industry. The disease is transmitted through *Diaphorina citri* vector, which inoculates *Candidatus* Liberibacter spp. in the plant sap. HLB disease lead to blotchy mottle and fruit deformation, among other characteristic symptoms, which induce fruit drop and affect negatively the juice quality. Nowadays, the disease is controlled by eradication of sick, symptomatic plants, coupled with psyllid control. Polymerase chain reaction (PCR) is the technique most used to diagnose the disease; however, this methodology involves high cost and extensive sample preparation. Mass spectrometry imaging (MSI) technique is a fast and easily handled sample analysis that, in the case of Huanglongbing allows the detection of increased concentration of metabolites associated to the disease, including quinic acid, phenylalanine, nobiletin and sucrose. The metabolites abieta-8,11,13-trien-18-oic acid, suggested by global natural product social molecular networking (GNPS) analysis, and 4-acetyl-1-methylcyclohexene showed a higher distribution in symptomatic leaves and have been directly associated to HLB disease. Desorption electrospray ionization coupled to mass spectrometry imaging (DESI-MSI) allows the rapid and efficient detection of biomarkers in sweet oranges infected with *Candidatus* Liberibacter asiaticus and can be developed into a real-time, fast-diagnostic technique.

## Introduction

Huanglongbing (HLB) disease affects different species of citrus, such as lemons, limes, oranges and mandarins. The disease causes the development of small and defective or lopsided fruits, while leaves show blotchy mottle, resulting in development of yellow shoots. Twig dieback, stunting, and tree decline are common symptoms in the affected trees^1,2^. The etiological agent of HLB disease is the bacteria *Candidatus* Liberibacter spp. This pathogen is transmitted when inoculated in the sap of citrus plants through the vector *Diaphorina citri* or *Trioza erytreae* and by grafting^3,4^. The etiological agent is widespread, so the species are named in accordance to the continent where they were initially identified (*Candidatus* Liberibacter asiaticus (*C*Las), *Candidatus* Liberibacter americanus (*C*Lam), *Candidatus* Liberibacter africanus (*C*Laf)), and *Candidatus* Liberibacter europaeus (*C*Leu)^1,5–7^. Currently the disease is responsible for the greatest economic loss in the citrus industry^8,9^.

The HLB disease has no cure, therefore diseased plants need to be eradicated as a control method^10,11^. Scouting is the way field inspector finds the symptomatic plants in the orchards. Symptoms are recognized and based on the training of the scouts. However, tree detection based on symptoms is inefficient, requiring several passes of diverse team to find all symptomatic trees^12^. The development of a faster and effective diagnostic tool for HLB disease is crucial to prevent the spread of the disease. Currently, the diagnosis of the disease in the symptomatic phase is mainly performed by the polymerase chain reaction (PCR) technique. However, the PCR methodology is expensive and demands a time-consuming sample preparation. Furthermore, the evaluation of HLB disease through *C*Las detection is negatively affected by its irregular distribution in leaves and by the low concentration of *C*Las cells during the initial phase of the latent period. In addition, the technique has limitations in differentiating viable and dead bacterial cells from each other, leading to an inaccurate quantification^13–15^. Efforts have been made in order to have a successful disease diagnostic through other analytical platforms, such as nuclear magnetic resonance (NMR) spectroscopy^16,17^, infrared spectroscopy (IV)^18^, capillary electrophoresis with diode array detection (CE-DAD)^19^, differential mobility spectrometry (DMS)^20^, and confocal Raman microscopy^21^. Despite this, these techniques sometimes require a time-consuming sample preparation methodologies and generate complex data that need a time-demanding statistical data treatment (spectroscopic methods) or final adjustments for spatial resolution (microscopic methods)^22–25^.

Mass spectrometry imaging (MSI) enables variations of metabolites to be identified on tissue sample regions on the cellular or subcellular level. This special feature allows new possibilities in chemical biology research^26,27^. MSI analyses are suitable for complex surfaces because it allows the detection of hundreds to thousands of compounds, such as lipids, proteins, and low molecular weight molecules. Furthermore, MSI indicates the spatial distribution of compounds in the sample surface^28–31^.

MSI was applied to analyze leaves from *Citrus sinensis* grafted on *Citrus limonia* infected by *Xylella fastidiosa*. The technique was used with success to monitor the presence of hesperidin and rutin in the infection site^32^. MSI was also applied to monitor other plant diseases^33–35^; however, this technique has never been applied to understand the infection process of *C*Las in citrus. Herein, we applied for the first time MSI analyses combined with HPLC–MS/MS to investigate healthy and diseased leaves (both asymptomatic and symptomatic stages) in the search for HLB biomarkers. We believe that these results may further help understanding the pathogenicity of *C*Las.

## Materials and methods

### Sample collection

Valencia sweet orange (*Citrus sinensis* (L.) Osbeck) grafted in Rangpur lime (*Citrus limonia* Osb.) rootstocks infected by grafting with budsticks carrying *C*Las, were kept in the greenhouse under standard phytosanitary and nutritional conditions, protected from the outside by screen, avoiding the entrance of insect vectors. The greenhouse contained a pad-fan to avoid excessive heating, but temperature fluctuations still occurred in correspondence to the environmental conditions. Healthy and *C*Las-infected plants were pruned 13 months before sampling and allowed to grow. PCR-positive trees showed typical blotchy mottle in older leaves, while younger leaves from PCR-positive trees were mostly asymptomatic. In the sampling analyses carried out before pruning, it was found that *C*Las was detected in leaves of each tree and none in the healthy tree. DNA was extracted from midribs of the leaves^36^ and *C*Las was detected using hydrolyzing probes marked with 5-FAM and quenched with BHQ (Macrogen, Seoul, Korea) according to Li et al.^37^, considering as positive samples with Ct below 35.0 and negative those with Ct values above 35.

Leaves at stage V7^38^ were collected directly in the greenhouse from five healthy plants (negative in qPCR analysis), three asymptomatic but *C*Las infected-plants confirmed by qPCR, and five symptomatic plants (*C*Las infected confirmed by qPCR). The leaf samples were placed inside individual plastic bags and stored at 4 °C until analyses. Healthy and *C*Las-infected plants were subjected to the same cultural practices, with the same scion/rootstock combination, and from the same age, handling, storage and nutritional treatments^39,40^ in order to avoid metabolic changes in the leaves that were not related to the infection process. The diseased status of the plants was the only contrasting characteristic between healthy and *C*Las-infected. Symptomatic samples were selected based on the presence of blotchy mottle, the most conspicuous symptom displayed by HLB-infected plants. Asymptomatic leaves were from the *C*Las-infected trees, but without any typical HLB symptom.

### DESI-MSI analyses

**Figure 1:**
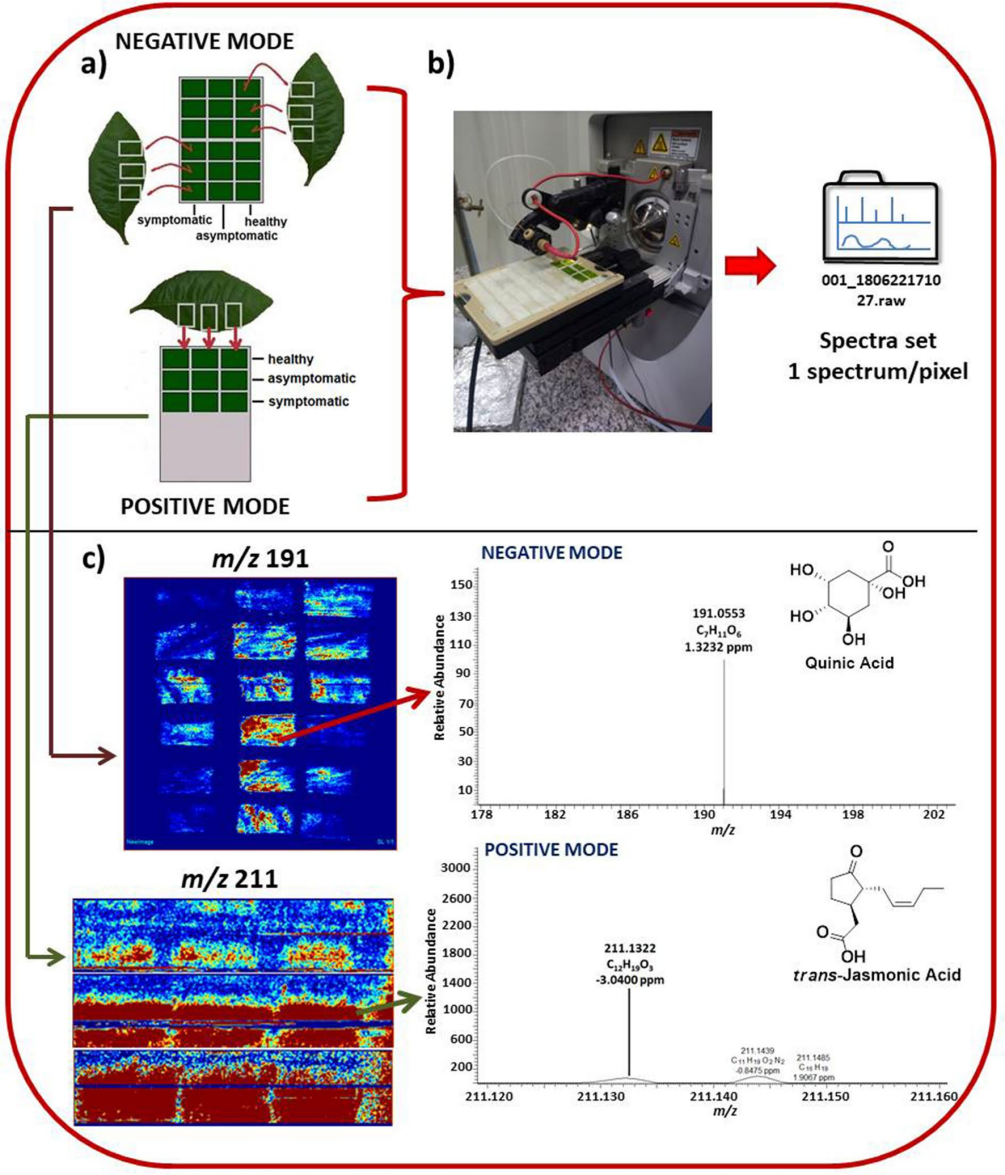
Image acquisition for mass spectrometry imaging of abaxial side of leaves. (**a**) Preparation of samples for leaf metabolite analysis on the plate in negative and positive mode; (**b**) scanning of leaf tissue surface and acquisition of the spectrum set; (**c**) DESI-MSI of the *m/z* 191 that corresponds with quinic acid and *m/z* 211 that corresponds to jasmonic acid in negative and positive mode, respectively. The exact masses were found in Xcalibur software.

The leaf preparation for DESI-MSI analyses was based on scientific literature that reviews methodologies for sample preparation of plants such as *Arabidopsis thaliana* and *Lotus japonicus*^26,27^. Firstly, the abaxial side of the leaves were lightly scraped with a small scalpel, cut in square format, and glued with tape on the glass plate (Figures S1 to S8 in Supplementary Information). Each horizontal row was immediately analyzed to avoid dryness of the sample, which would impair the analysis. The plates were prepared in accordance to Fig. 1, and the analyses were performed four times with different samples from the same treatment.

The analyses were performed in a Thermo Scientific Q-Exactive MSI using desorption electrospray ionization (DESI) source (Omni Spray 2D-3201 model—Prosolia, Indianapolis, USA). The values range of mass-to-charge (*m/z*) scanned was between 100 and 1,000, both in positive and negative modes. Other parameters adjusted were spray voltage of 1.5 kV, capillary temperature at 250 °C, S-lens of 100.00, pixel scan of 200 µm, resolution of 17,500 (*m/*Δ*m* at *m/z* 200)^41^, flow rate of 5 µL min^−1^ and a scanning rate of 740 μm s^−1^. In the DESI-MSI analyses conducted, a sample size of 1.5 cm × 2.0 cm (length × width) for each leaf region was used. Images were processed with the use of BioMAP 3.8.0.4 (Novartis, Basel, Switzerland) software. For each *m/z* value obtained, an image was generated; all images were processed with a bin size of 4.0 and standardized with the same intensity level (5.0 in the intensity level bar). The exact mass was obtained using Thermo Xcalibur Roadmap software 3.0.63 (Waltham, MA, USA).

### Sample preparation for LC–MS/MS analyses

The same groups of leaf samples used in previous analyses were macerated with liquid nitrogen. Three leaves from the symptomatic, asymptomatic, and healthy groups were macerated together. It was weighed 0.2 g of the leaves, which was extracted with 2 mL of different organic solvents. The solvents were tested to optimize metabolite extraction; ethyl acetate, dichloromethane, and methanol were evaluated. The samples were vortexed and were dried under a nitrogen flow in order to remove the extraction solvent. Then, the samples were dissolved in 1 mL of HPLC-grade methanol and filtered using a Millipore PTFE hydrophobic membrane filter with a 0.22 μL pore diameter.

The HPLC–MS analyses were performed three times in order to evaluate reproducibility. The first analysis was done in negative mode with 18 leaves of each group (asympotamic, symptomatic and healthy groups), been six samples per group, totalizing 54 leaves and 18 spectra; the second analysis was done in negative mode with nine leaves of each group, been three samples per group, totalizing 27 leaves and nine spectra. The third analysis was done in positive mode and in duplicate with nine leaves of each group (3 samples/group), totalizing 27 leaves and 18 spectra (Figure S9 in Supplementary Information).

### LC–MS/MS analyses

The analyses were performed using ultra performance liquid chromatography (Shimadzu, Nexera X2, Japan) coupled to mass spectrometry (Impact II, Bruker Daltonics Corporation, Germany). It was used an Acquity UPLC CSH C18 column with 2.1 × 100 mm (Waters, Ireland), 2.1 μm of particle size and flow rate 0.200 mL min^−1^. The mobile phase was composed of 1% formic acid in water (*v/v*) and 0.1% methanol in formic acid (*v/v*) at 40 °C.

The analyses were performed using a quadrupole time-of-flight mass analyzer (QTOF) with a collision energy ramp of 10–45 eV. The mass range (*m/z*) scanned was between 50 and 1,400 with an acquisition rate of 5 Hz for MS and 7 Hz for MS/MS in positive mode [M + H]^+^ with end plate offset potential − 500 V. The four most intense ions were selected for automatic fragmentation (AutoMS/MS). Spectra were processed in DataAnalysis 4.2 software.

### Metabolites identification

The molecular formulas obtained in addition to the MS/MS fragmentation pattern were submitted to different databases for identification. The results obtained were compared with human metabolome database (HMDB)^42^, global natural product social molecular networking (GNPS)^43^, dictionary of natural products (DNP)^44^, and literature^16,45–49^. The GNPS analyses for dereplication (or library search) were performed with precursor ion mass tolerance of 0.2 and fragment ion mass tolerance of 0.1, which are selected parameters in the GNPS related to mass tolerance and how much the fragment ion can be shifted from expected *m/z* values, respectively. For molecular networking, it was used precursor ion mass tolerance of 2.0, fragment ion mass tolerance of 0.5, and a minimum cosine score (min. pairs cos.) of 0.7. In dereplication the search for similarity of mass spectra is done based on databases available in the GNPS, while molecular networking enables discover new molecules and chemical species from databases and data submitted by users of the GNPS.

## Results and discussion

### Sample preparation and image acquisition

HLB disease was responsible for the eradication of approximately fifty million orange trees (100,000 ha) from 2004 to 2018 in São Paulo state, Brazil^50^ and 72.2% reduction of orange production in United States between 2007 and 2018, implicating in huge losses for worldwide citriculture^51^.

Currently, HLB disease detection methods are time consuming and involve multiple stages of sample preparation. Efforts have been done to have an early diagnostic of the disease with no sucess^16–21^. Due to these reasons, our group has been focusing attention on HLB disease and a new potential diagnostic method. Therefore, in order to monitor the metabolic profile of healthy leaves from *Citrus sinensis* compared to the diseased metabolic profile in asymptomatic and symptomatic stages of *C*Las infection, we applied for the first time mass spectrometry imaging technique. These studies would potentially contribute to helping understand the metabolites related to plant defense process, stress response, or those potentially produced as virulence factor by the pathogen.

Sample preparation for MSI analysis is relatively simple compared to other techniques such as those used within molecular biology. Healthy, PCR-negative sweet orange nursery trees were used as controls, with Ct values above the threshold limit and most often with undetectable fluorescence in qPCR analysis^37^ (data not shown), while *C*Las-infected samples had average Ct values of 20.9–23.9 for the asymptomatic leaves and Ct 19.1–21.6 for symptomatic leaves. It is possible to extract data using a leaf tissue surface with the size ranging to millimeter to centimeters and a scan time/pixel about 0.96–1.6 s^52–54^, which makes MSI a potential diagnostic tool to be implemented for a real-time detection of HLB in the field. Different leaf regions were fixed on the surface of a plate (Fig. 1) in order to cover different areas of the sample surface and detect a larger number of metabolites that may be distributed throughout the leaf. Figure 1 indicates the main steps for DESI-MSI image acquisition.

Another sample preparation strategy used in our studies for imaging was the imprinting procedure^31^; however, we chose to perform the direct analysis in the leaves tissue due to the similar results obtained in both strategies. The direct analysis led to almost zero sample preparation and faster diagnostics.

### Metabolites identification

Biomap software was used for image processing. All the images acquired by MSI were standardized to have the same coloring in a blue-red scale. The metabolites accumulated in the disease are represented by the red color. Therefore, the most intense peak (100%) is indicated by red coloration, while other colors are proportional to its relative intensity^55^.

The metabolites accumulated in the affected plants that were identified (putatively annotated compounds—Table 1) showed an exact mass compatible with an elemental composition with low value of delta (below 4.5 ppm) for all the results.

**Table 1:** Metabolites putatively identified using DESI-MSI (*full scan*) predominantly produced in diseased samples of sweet orange leaves.

Entry	Metabolites	Experimental *m/z* (Xcalibur)	Database *m/z* (HMDB)	Error (ppm)	Mode (DESI-MSI)	References associated with HLB disease or plant defense	Figures in Supplementary Information
1	Abieta-8,11,13-trien-18-oic acid (Dehydroabietic acid)	301.2162	301.2162	0.24	Positive	^56^	S10–S12
2	Abscisic acid (ABA)	265.1434	265.1440	− 2.77	Positive	^57^	S13–S16
3	4-Acetyl-1-methylcyclohexene (4-AMCH)	139.1117	139.1123	− 0.08	Positive	^58,59^	S17–S20
4	Asparagine	133.0608	133.0613	3.17	Positive	^51,60^	S21–S24
5	Feruloylputrescine	265.1547	265.1552	− 1.92	Positive	^47^	S25–S28
6	*β*-Glucose	179.0550	179.0556	1.59	Negative	^16,45,61^	S29–S31
7	Guaiacol	125.0597	125.0603	1.47	Positive	^62^	S32–S35
8	*p*-Hydroxycinnamic acid (*p*-coumaric acid)	165.0546	165.0552	2.24	Positive	^47,51,63^	S36–S39
9	Isoleucine	132.1019	132.1025	1.10	Positive	^45,46^	S40–S43
10	*trans*-Jasmonic acid (*t*-JA)	211.1329	211.1334	− 3.04	Positive	^46,57^	S44–S47
11	Nobiletin	403.1387	403.1393	− 1.05	Positive	^48,51^	S48–S50
12	Phenylalanine	166.0863	166.09	0.57	Positive	^45,46,49,51^	S51–S53
13	Pipecolic acid	130.0863	130.0868	0.81	Positive	^45,46^	S54–S57
14	Quinic acid	191.0553	191.0556	1.34	Negative	^64^	S58–S60
15	Sucrose	343.1235	343.1235	2.89	Positive	^16,61^	S61–S63
16	Synephrine	168.1019	168.1025	− 0.15	Positive	^49^	S64–S66
17	Tangeretin	373.1282	373.1287	− 0.88	Positive	^48,51^	S67–S69
18	Tetramethoxyflavone (TMF)	343.1176	343.1182	− 1.12	Positive	^65^	S70–S73
19	Tryptophan	205.0972	205.0977	− 1.34	Positive	^16,46^	S74–S77
20	Tyrosine	182.0812	182.0812	− 0.44	Positive	^16,66^	S78–S81
21	Valine	118.0863	118.0868	1.40	Positive	^16,46^	S82–S85

The metabolites shown in Table 1 were previously reported in citrus by using chromatography and mass spectrometry techniques: dehydroabietic acid, ABA, 4-acetyl-1-methylcyclohexene (4-AMCH)^56–58^, asparagine, pipecolic acid, quinic acid^45^, guaiacol^67^, isoleucine, *t*-JA, phenylalanine, tryptophan, valine^46^, feruloylputrescine, hydroxycinnamates (HCAs)^47^, nobiletin and tangeretin^48^. Other metabolites in Table 1 were identified previously by NMR spectroscopy: synephrine^49^, *β*-glucose, sucrose, tryptophan, tyrosine and valine^16^.

**Figure 2:**
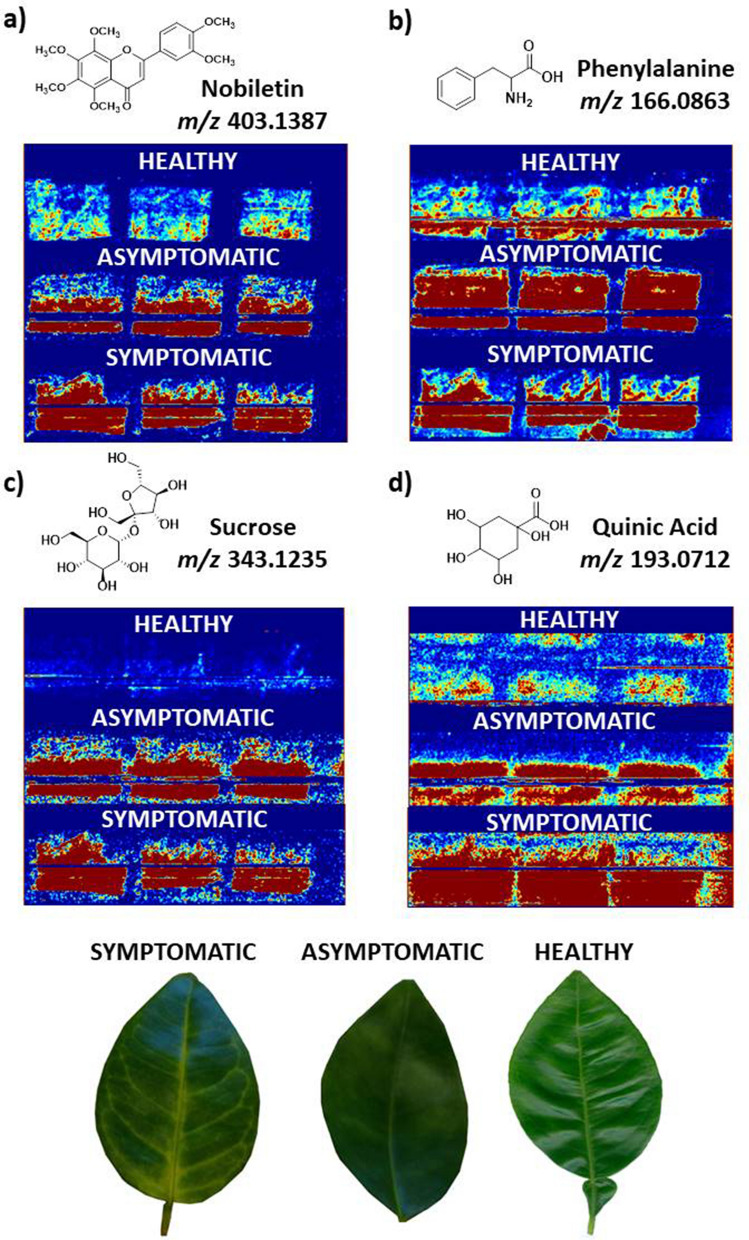
Biomarkers of HLB disease identified for DESI-MSI analyses in positive mode. In the images acquired, the red color indicates the ion distribution on the abaxial leaf surface in asymptomatic, symptomatic and healthy samples. The images acquired were at (**a**) *m/z* 403; (**b**) *m/z* 166, (**c**) *m/z* 343, and (**d**) *m/z* 193, which the exact masses found in Xcalibur software (*m/z* 403.1387, *m/z* 166.0863, *m/z* 343.1235, and *m/z* 193.0712) correspond to nobiletin, phenylalanine, sucrose, and quinic acid, respectively.

Based on the DESI-MSI analyses conducted for this study, it was observed an increase in the accumulation of nobiletin (Fig. 2), tangeretin, feruloylputrescine, synephrine, and asparagine after the increase of disease symptoms in leaves. These results corroborate with quantification data obtained for HPLC for the feruloylputrescine^47^, nobiletin, and tangeretin^48^ and NMR data for synephrine^49,60^. Furthermore, nobiletin has previously been associated as a biomarker of HLB disease in the fruit juice^68^.

Other metabolites such as abscisic acid (ABA), asparagine, glucose, *p*-hydroxycinnamic acid, isoleucine, *trans*-jasmonic acid (*t*-JA), phenylalanine, quinic acid, sucrose, tryptophan, and valine had an increase in concentration in the diseased leaves (images in Supplementary Information—Figures S16, S22, S31, S39, S43, S47, S53, S60, S63, S77, and S85, respectively), which corroborate with quantification data obtained for GC–MS (tryptophan, *t*-JA, phenylalanine, isoleucine, valine in *C*Las-infected)^46,69^, GC–MS-SIM (ABA, *t*-JA and tryptophan in *C*Las-infected)^57^, UV–Vis (hydroxycinnamates)^63^, starch assay (sucrose and glucose)^61^ and NMR data (asparagine and quinic acid)^60^. Figure 2 shows the image acquired for DESI-MSI for four metabolites (nobiletin, phenylalanine, sucrose and quinic acid) that are clearly differentiated in the diseased leaves and that were previously associated as biomarkers for HLB disease^16,61,64,68^, indicating that MSI technique would be a potential great tool for real-time diagnostics of HLB. The images and *m/z* obtained for each of the metabolites mentioned in Table 1 are available within the Supplementary Information (Pages S8–S55).

### Flavonoids, flavones and compounds from orange

Nobiletin, tangeretin, tetramethoxyflavone, and feruloylputrescine were reported in leaf samples from *Citrus sinensis* (L.) Osbeck^65,68,70^ while synephrine and 4-acetyl-1-methylcyclohexene in orange juice^58,60^.

Hijaz, et al*.* investigated the production of tangeretin and nobiletin in HLB disease. The authors confirmed the accumulation of both metabolites in leaf samples in weeks 27 and 29, respectively^47^. Furthermore, Massenti et al. quantified tangeretin and nobiletin for HPLC presents in peels of Valencia orange fruits and they observed the increase of these metabolites in diseased fruits, which corroborates with our data of DESI-MSI for the leaf samples (Figures S50 and S69 in Supplementary Information)^48^.

4′,5,6,7-Tetramethoxyflavone (or tetramethyl-O-scutellarein) also accumulated in the infected leaves based on DESI-MSI analyses (Figure S73). This metabolite is a polymethoxyflavone (PMF), as well as, nobiletin (5,6,7,8,3,4′-hexamethoxyflavone) and tangeretin (5,6,7,8,4′-pentamethoxyflavone)^65^. The change in the concentration of PMF is based on fruit maturity and such compounds have been associated to plant defense process against pathogens^65^. Hijaz et al*.* observed an increased concentration of 4′,5,6,7-tetramethoxyflavone on week 29 which is in accordance to our DESI-MSI data (Figure S73 in Supplementary Information)^47^.

Feruloylputrescine is a secondary metabolite from orange that is a conjugate of putrescine and ferulic acid^51^. The increase of feruloylputrescine production levels in *C*Las infection was quantified by HPLC–MS for Baldwin et al. in orange juice, which corroborate with our MSI data (Figure S28 in Supplementary Information)^71^.

Guaiacol production is a response to *C*Las infection based on the DESI-MSI analyses (Figure S35 in Supplementary Information), since it was observed in higher concentration in infected leaves. To the best of our knowledge, guaiacol had never been reported as a biomarker to HLB disease. However, an increasing level of catechol O-methyltransferases (enzyme that synthesizes guaiacol) occur in infected plants^62^, which may result in an increase in guaiacol concentration.

Another metabolite detected by the DESI-MSI was synephrine (Figure S66 in Supplementary Information). Synephrine is an alkaloid of occurrence in *Citrus* of Rutaceae family and is known for its natural antihistaminic effect and ability to induce lipolysis^72–74^. Although the increase of synephrine levels in fruits infected by *C*Las is well reported in literature^49,60^, its role in the infection process remains unclear.

4-Acetyl-1-methylcyclohexene, a volatile oxidation product of limonene, also accumulated in the plants infected with *C*Las as indicated in our DESI-MS analyses. This compound is present in citric peel oil, orange juice, and other food sources^58,75–76^. Tao et al*.* verified that 4-AMCH is part of the essential oil of Ponkan (*Citrus reticulata* Blanco) composition, which showed antibacterial and antifungal activities against *Escherichia coli* and *Bacillus subtilis*^77^. So far, 4-AMCH has not been associated to *Candidatus Liberibacter* spp. infection and HLB disease.

### Amino acids

Asparagine, isoleucine, phenylalanine, tryptophan, tyrosine, and valine were the amino acids detected by DESI-MSI analyses, showing a higher concentration in diseased leaves (Figures S22, S43, S53, S77, S81, and S85 in Supplementary Information, respectively). Isoleucine was detected by LC–MS/MS analysis in leaf samples (Figure S41 in Supplementary Information), and this accumulation was also reported in *C*Las infected leaves^46^. The increase of valine production in *C*Las infected leaves and fruit was also reported^69^. The role of the amino acids isoleucine, valine, and tyrosine in the *C*Las infection process is not yet fully understood; however, it is known that *C*Las is incapable to produce such amino acids^78^. The same amino acids were detected in the flush shoots of Rutaceae host plants, flush shoots of *D. citri* nymphs^66^, and citrus leaves during defense responses to stress^45^.

DESI-MSI also indicated the increase of phenylalanine, tryptophan, and asparagine. Phenylalanine and tryptophan are precursors of the phytohormone salicylic acid (SA) and the auxin indole-3-acetic acid (IAA), respectively. SA and IAA are signaling compounds and may have a role in *C*Las as well as *D. citri* infection process in sweet oranges^57^. The increase of asparagine levels observed by DESI-MSI in Valencia oranges during the plant-pathogen interactions corroborates with literature^59,68^ and was reported^66^ as an abundant free amino acid (FAA) in permissive hosts that may be modulated by *C*Las.

Although DESI-MSI is able to differentiate healthy and diseased profiles through images of isoleucine, tryptophan, and phenylalanine, amino acids are not specific biomarkers for HLB disease. The variations in the levels of amino acids, as well as organic acids, are a common response during the disease process^45–46^.

### Phytohormones

Phytohormones associated with the defense response of *C*Las infection and *D. citri*-infestation, such as ABA and *t*-JA, are also detected at higher concentrations by DESI-MSI (Figures S16 and S47, respectively in Supplementary Information). Absisic acid is a phytohormone associated with many plant physiological functions, including, plant defense response to abiotic stress and disease tolerance in different types of plant–pathogen interactions^57,79^.

Jasmonic acid is a phytohormone that activates the expression of plant defense against herbivores as the *Diaphorina citri*^46,80^. Nehela et al. detected the phytohormones (auxins, ABA, SA and *t*-JA) of Valencia sweet orange leaves by GC–MS-SIM in different stages—healthy, *C*Las infected, *D. citri* infested, and double attacked. In this study they verified that ABA and SA are related to plant defense to *C*Las, while that *t*-JA is associated with the *D. citri* infestation^57^.

### Organic acids

Organic acids are commonly associated with plant stress^81,82^. This alteration in the metabolic profile of organic acids was also reported in plants infected by *C*Las^47,64^. Some organic acids, such as quinic acid and *p*-hydroxycinnamic acid, were previously detected and quantified in symptomatic leaves^47,64^, which corroborate with our MSI results (Figures S39 and S60 in Supplementary Information).

Quinic acid was identified in different varieties of healthy citrus^83^ and has been associated as a citrus leaf metabolite during defense responses^64^. In our analysis, the peak at *m/z* 191.0550 refers to quinic acid and the MS/MS fragmentation pattern indicates an ion at *m/z* 127.0399 corresponding to the loss of CO and two molecules of H_2_O^84^. A higher concentration of quinic acid was detected by DESI-MSI (Fig. 2) in asymptomatic profile followed by symptomatic profile. The increase in quinic acid level in diseased leaves was also reported by Jones et al.^64^, Chin et al.^60^ and Yao et al.^69^, corroborating with our DESI-MSI data. The increase in quinic acid production in the asymptomatic plants makes it a good biomarker to HLB disease as previously suggested by Jones et al.^64^.

Quinic acid is a primary organic acid present in leaves of sweet orange trees and can be converted to shikimic acid. Quinic acid and shikimic acid are precursors of other compounds, such as lignin, phenylalanine, and tyrosine^64,85^. Quinic acid has been detected in semi-tolerant variety of fruits to *C*Las^85^ and against other citrus pathogens as *Xylella fastidiosa*^86^; however, its role in the defense mechanism against the *C*Las is still not completely clear.

In our studies, *p*-hydroxycinnamic acid was also detected by DESI-MSI technique; the correspondent images obtained indicated an increase in the production of this metabolite in both asymptomatic and symptomatic profiles. The increase in hydroxycinnamates levels in oranges’ leaves during *C*Las infection was reported in literature^47,63^ and is associated to defense responses. The metabolite *p*-hydroxycinnamic acid is a precursor in the biosynthesis of phenylpropanoid derivatives that have antimicrobial activities and are related to plant defense^47,87^.

Abieta-8,11,13-trien-18-oic acid (Dehydroabietic Acid—DHA) was identified in GNPS analyses (gold classification) and was previously isolated by Vargas et al*.* from nonvolatile residue of citric essential oil. It is a metabolite found in resin acids of colophonium and can be present also in natural fragrance and when oxidized can act as allergen^88^. This metabolite is part of pine (*Pinus nigra*) resin’s constitution and is reported to be produced by the plant against pathogens^89,90^. DHA was also identified in *Chamaecyparis pisifera*^91^ and *Tripterygium wilfordii*^92^ and has been studied against various bacteria species due its antimicrobial activity^56,93,94^. However, this metabolite has never been associated with *C*Las infection, therefore more studies need to be performed to understand its role in HLB disease.

Another organic acid detected by DESI-MSI in both asymptomatic and symptomatic leaves was pipecolic acid^46,95^, that is biosynthesized from lysine and acts as an immune signal regulating the plant defense responses. Pipecolic acid has been suggested in literature^46,96^ to participate in the defense against *C*Las, inducing the SA-mediated pathway.

### Sugars

Sugar, such as sucrose and glucose, levels increasing in leaves infected by *C*Las can be observed in the images of DESI-MSI analyses (Figures S31 and S63 in Suplemmentary Information) and the higher levels are in accordance with previous studies^61,97^. During the infection process, sucrose and glucose act as signaling compounds, inducing the feedback inhibition of photosynthesis^61,98^.

**Figure 3:**
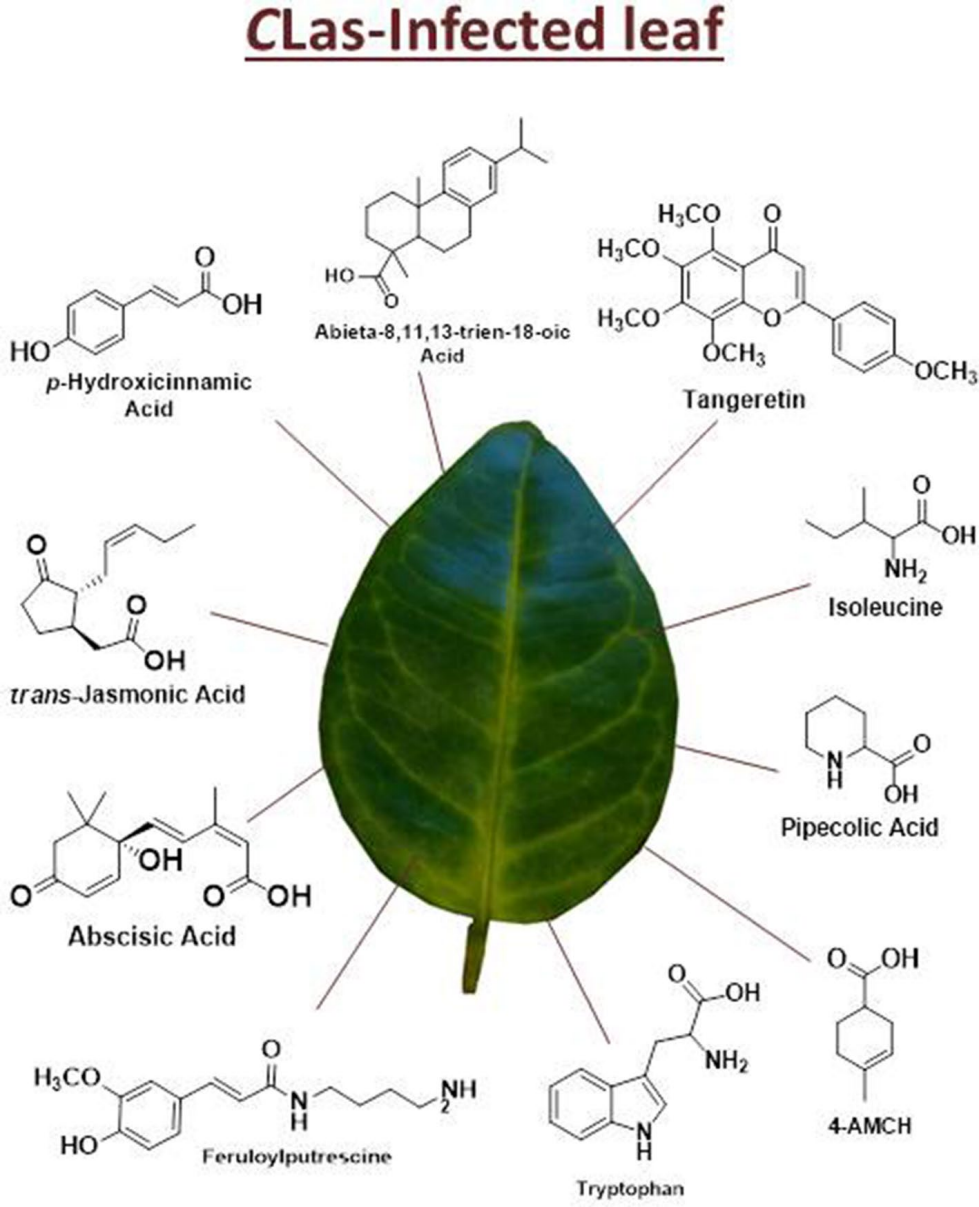
Potential biomarkers of HLB disease. The metabolites Abieta-8,11,13-trien-18-oic acid, abscisic acid, 4-acetyl-1-methylcyclohexene (4-AMCH), feruloylputrescine, *p*-hydroxycinnamic acid, isoleucine, *trans*-jasmonic acid, pipecolic acid, tangeretin, and tryptophan, which were detected by DESI-MSI had an increase of concentration in asymptomatic and symptomatic *C*Las-infected leaves. The increase of concentration is based on images acquired by DESI-MSI available in Supplementary Information (Figures S12, S16, S20, S28, S39, S43, S47, S57, S69, and S77, respectively).

Figure 3 summarizes the major metabolites identified for DESI-MSI analyses involved in the HLB disease.

Mass spectrometry imaging proved to be a suitable technique to identify a range of metabolites regulated during the *C*Las-sweet orange interaction, identify compounds produced in higher levels in both the asymptomatic and symptomatic stages, and indicate potential biomarker candidates with potential to develop an early diagnostic of HLB disease. Furthermore, MSI analysis showed to be a fast technique with little sample preparation and manipulation, fulfilling requirements compatible with a diagnostic process. There are examples of the accumulation of compounds in the healthy leaves as shown in Figure S86 in Supplementary Information; however, here we discussed the metabolites that had an increase in the diseased leaves and could be potential biomarkers.

Quinic acid, phenylalanine, nobiletin, and sucrose are metabolites that were previously reported in literature as biomarkers of HLB disease in orange fruits^16,61,64,68^. In addition to metabolites associated to plant defense or stress such as quinic acid, *t*-JA and tryptophan^45,57^ were also detected by DESI-MSI in asymptomatic leaves of *Citrus sinensis* with significant difference of healthy profile (Figs. 2 and S47 and S77 in Supplementary Information). We believe that these metabolites could be a potential parameter for infection recognition still in the asymptomatic stage; however, further validation to use the DESI-MSI as a diagnostic tool is necessary. Other metabolites that are potential biomarkers of HLB disease in the asymptomatic stage are: ABA, abieta-8,11,13-trien-18-oic acid, 4-AMCH, feruloylputrescine, *p*-hydroxycinnamic Acid, isoleucine, pipecolic acid, tangeretin, and TMF.

## Conclusions

Through MSI analyses, it was possible to identify metabolites that participate in the *C*Las infection process and plant defense, such as phytohormones, organic acids, carbohydrates, flavonoids, and amino acids that previously were reported in literature^46,48,57,61^. The images provide a better understanding of *m/z* distribution on the leaf’s surface in the different HLB disease stages. The detection of these metabolites in asymptomatic leaves is important as a potential technique for HLB diagnosis, opening possibilities to search for new biomarkers. The metabolites abieta-8,11,13-trien-18-oic acid, suggested by GNPS, and 4-acetyl-1-methylcyclohexene showed a higher distribution in symptomatic leaves. These metabolites have been directly related with HLB disease and needs to be further investigated concerning the function in the *C*Las infection.

## Supplementary information

Supplementary information.
