# Supplementary material for: Mass spectrometry imaging as a potential technique for diagnostic of Huanglongbing disease using fast and simple sample preparation
Source: Sci Rep. 2020 Aug 10;10:13457. doi: 10.1038/s41598-020-70385-4 (PMC7417563; doi:10.1038/s41598-020-70385-4)
Supplement: Supplementary file 1 — Supplementary information. [file 41598_2020_70385_MOESM1_ESM.pdf]

Supplementary Information

Mass Spectrometry Imaging as a potential technique for diagnostic of Huanglongbing disease using fast and simple sample preparation

João Guilherme de Moraes Pontes[1], Pedro Vendramini[2], Laura Soler Fernandes[1], Fabricio Henrique de Souza[3], Eduardo Jorge Pilau[4], Marcos Nogueira Eberlin[2], Rodrigo Facchini Magnani[5], Nelson Arno Wulff[5], Taicia Pacheco Fill[*1]

1- Laboratório de Biologia Química Microbiana (LaBioQuiMi), IQ-UNICAMP, Brazil

2- ThoMSon Mass Spectrometry Laboratory, IQ-UNICAMP, Brazil

3- Programa de Pós-graduação em Ciências Biológicas, Universidade Estadual de Maringá, Maringá-PR, Brazil

4- Laboratório de Biomoléculas e Espectrometria de Massas (LabioMass), Universidade Estadual de Maringá, Departamento de Química, Maringá-PR, Brazil

5- Departamento de Pesquisa & Desenvolvimento, Fundo de Defesa da Citricultura (FUNDECITRUS), Araraquara-SP, Brazil

# Corresponding author: Tel. +55-19-3521-3092

- e-mail address: taicia@unicamp.br

Summary

Sample preparation - Negative mode MSI analyses	S3
Sample preparation - Positive mode MSI analyses	S5
Reproducibility of HPLC-MS analyses	S7
Abieta-8,11,13-trien-18-oic acid	S8
Abscisic acid	S10
4-Acetyl-1-methylcyclohexene	S13
Asparagine	S16
Feruloylputrescine	S18
β-Glucose	S20
Guaiacol	S22
p-Hydroxycinnamic acid	S25
Isoleucine	S28
trans-Jasmonic acid	S30
Nobiletin	S33
Phenylalanine	S35
Pipecolic acid	S37
Quinic acid	S40
Sucrose	S42
Synephrine	S44
Tangeretin	S46
4',5,6,7-Tetramethoxyflavone	S48
Tryptophan	S50
Tyrosine	S52
Valine	S54
Accumulation of metabolites in healthy metabolic profile	S56

NEGATIVE MODE MSI ANALYSES

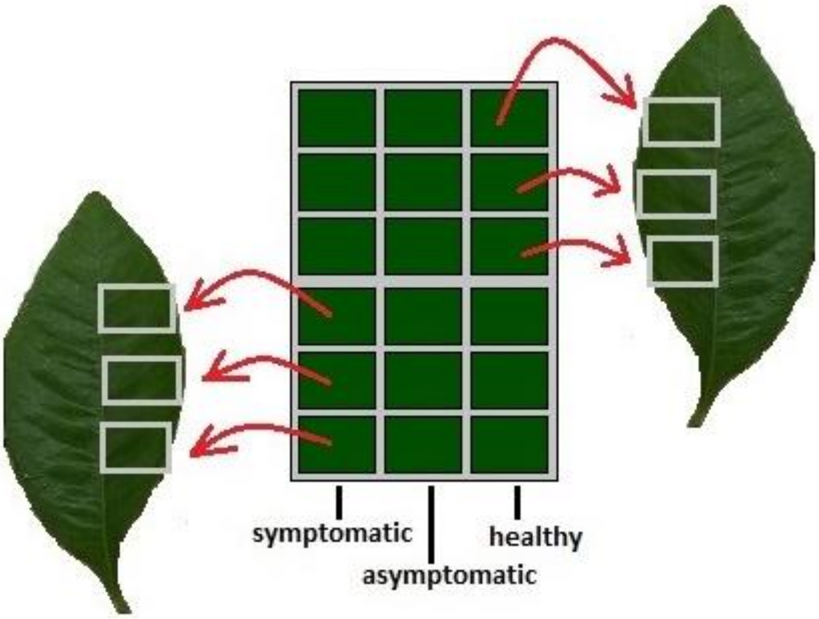

Figure S1. Sample preparation for negative mode MSI analyses

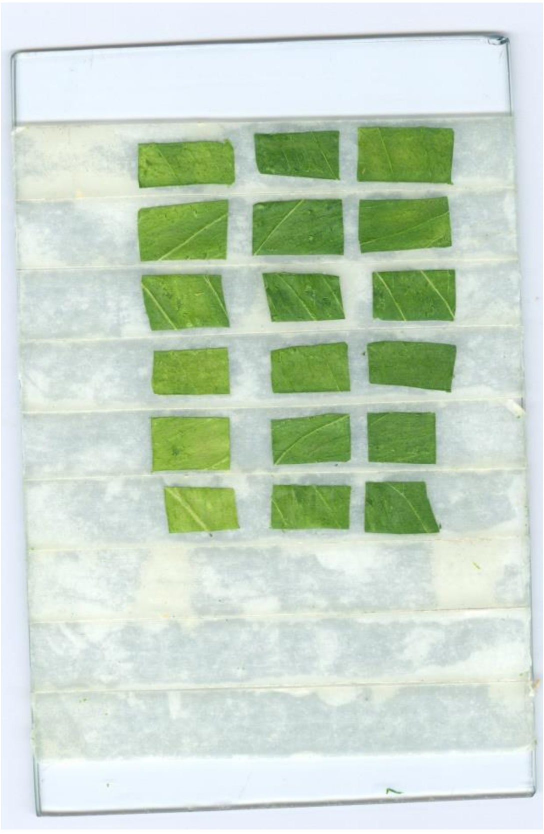

Figure S2. Photo of surface of samples for negative mode MSI analysis

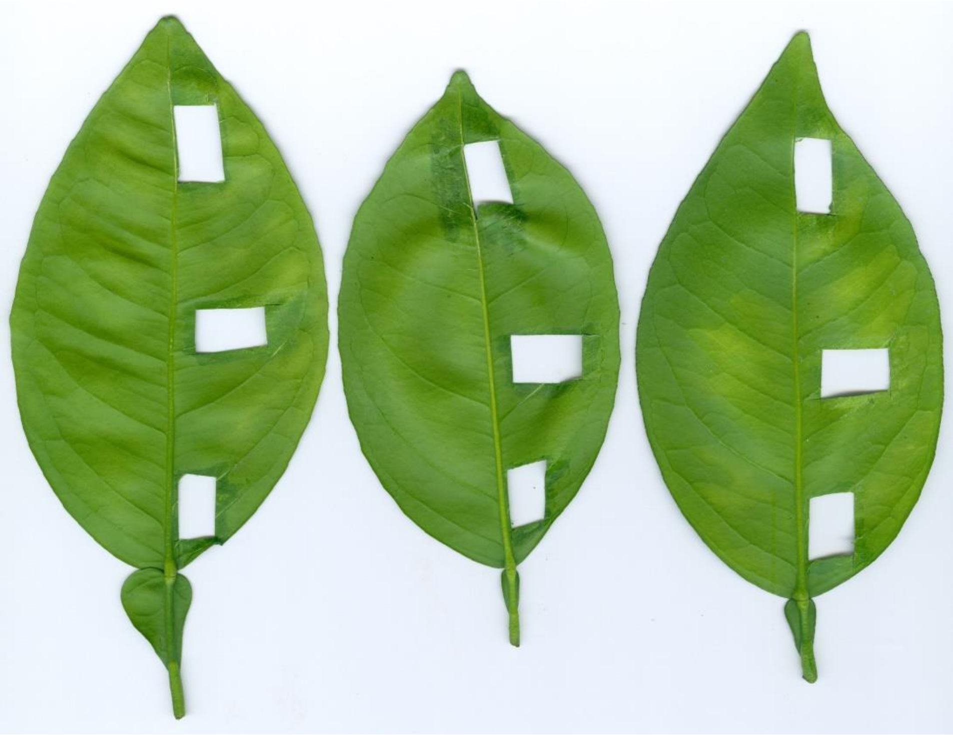

Figure S3. Healthy sample, asymptomatic sample and symptomatic sample from left to right, respectively (top of the plate)

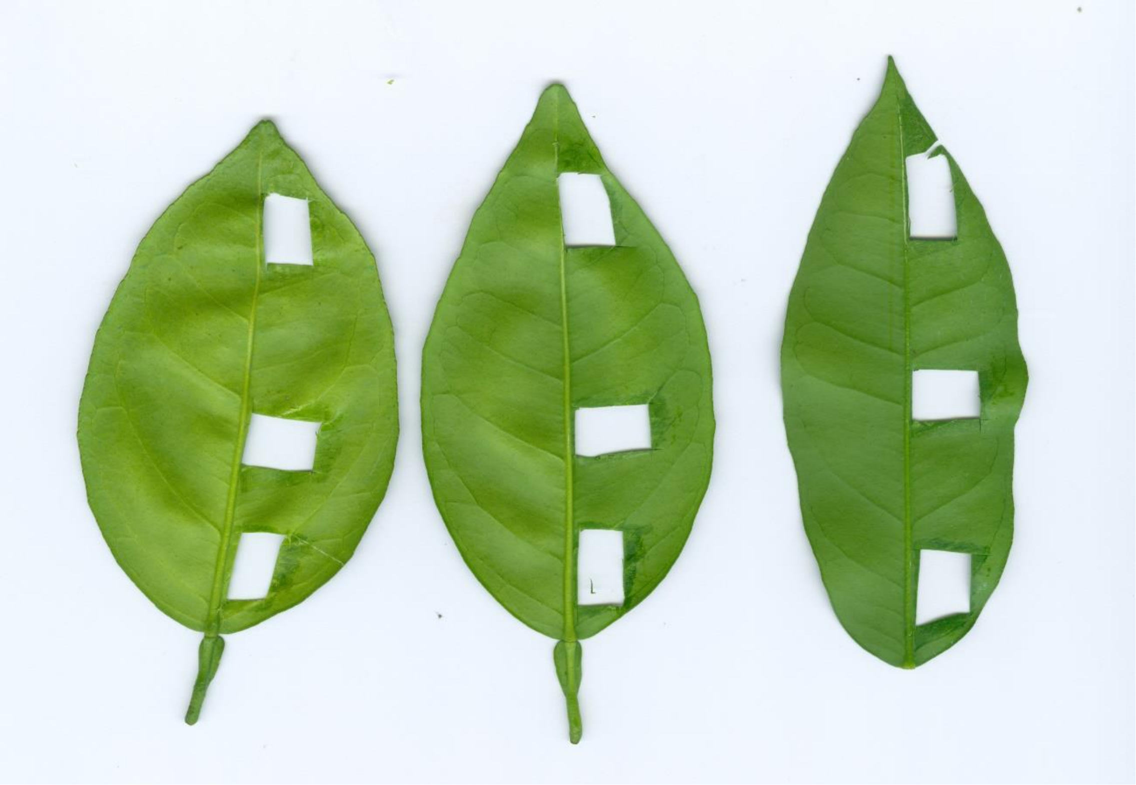

Figure S4. Symptomatic sample, asymptomatic sample and healthy sample from left to right, respectively (Bottom of the plate)

POSITIVE MODE MSI ANALYSES

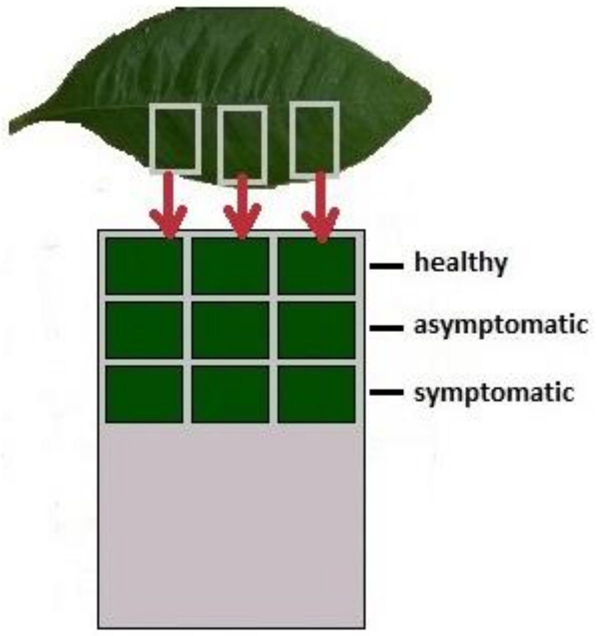

Figure S5. Figure of sample preparation for positive mode MSI analyses

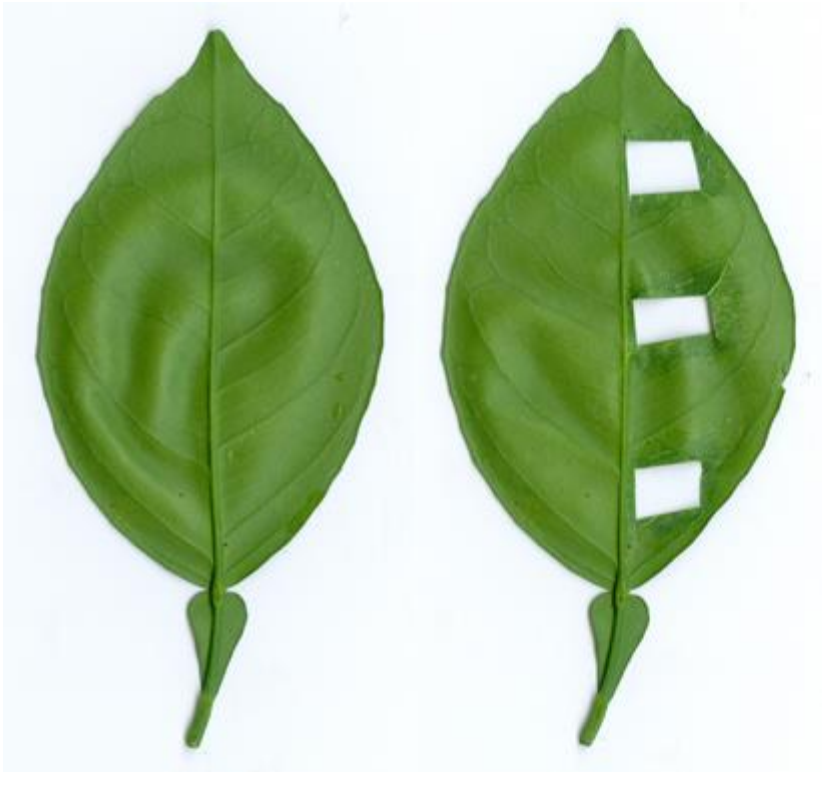

Figure S6. Healthy Leaf

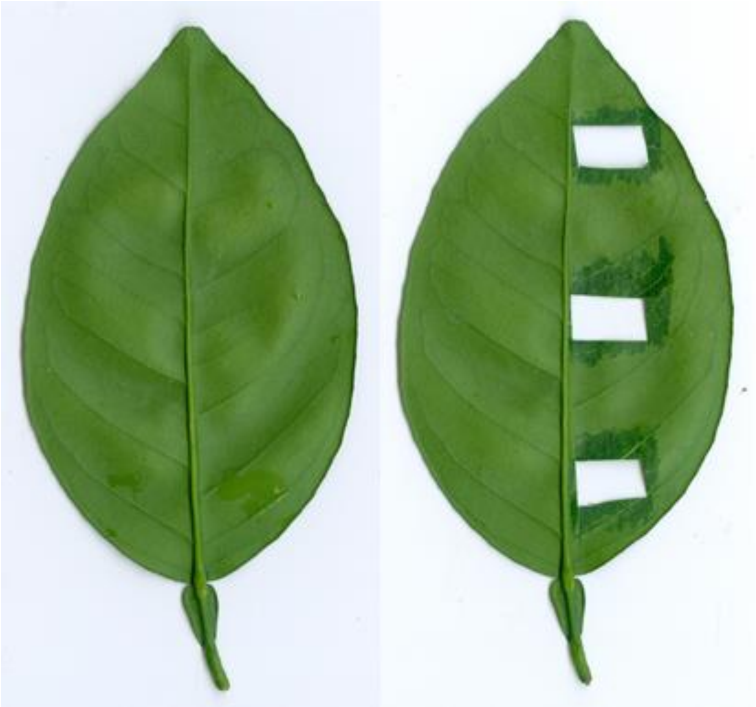

Figure S7. Asymptomatic Leaf

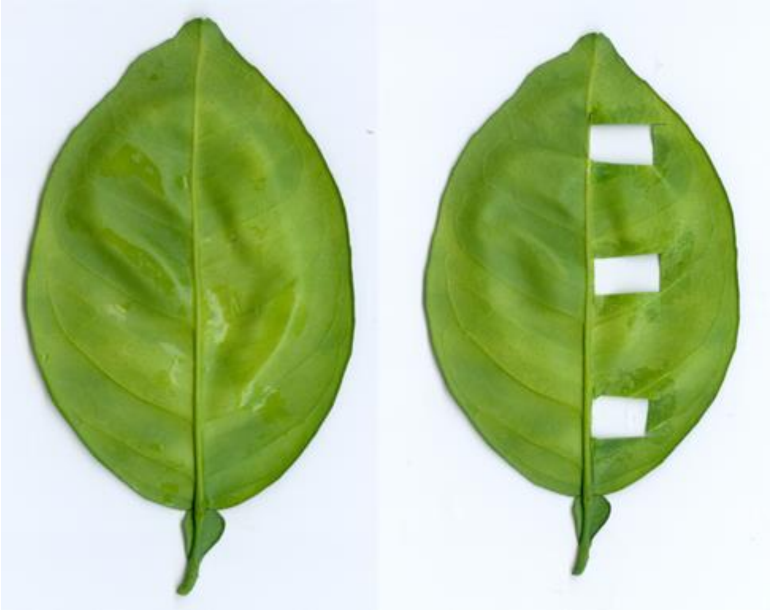

Figure S8. Symptomatic Leaf

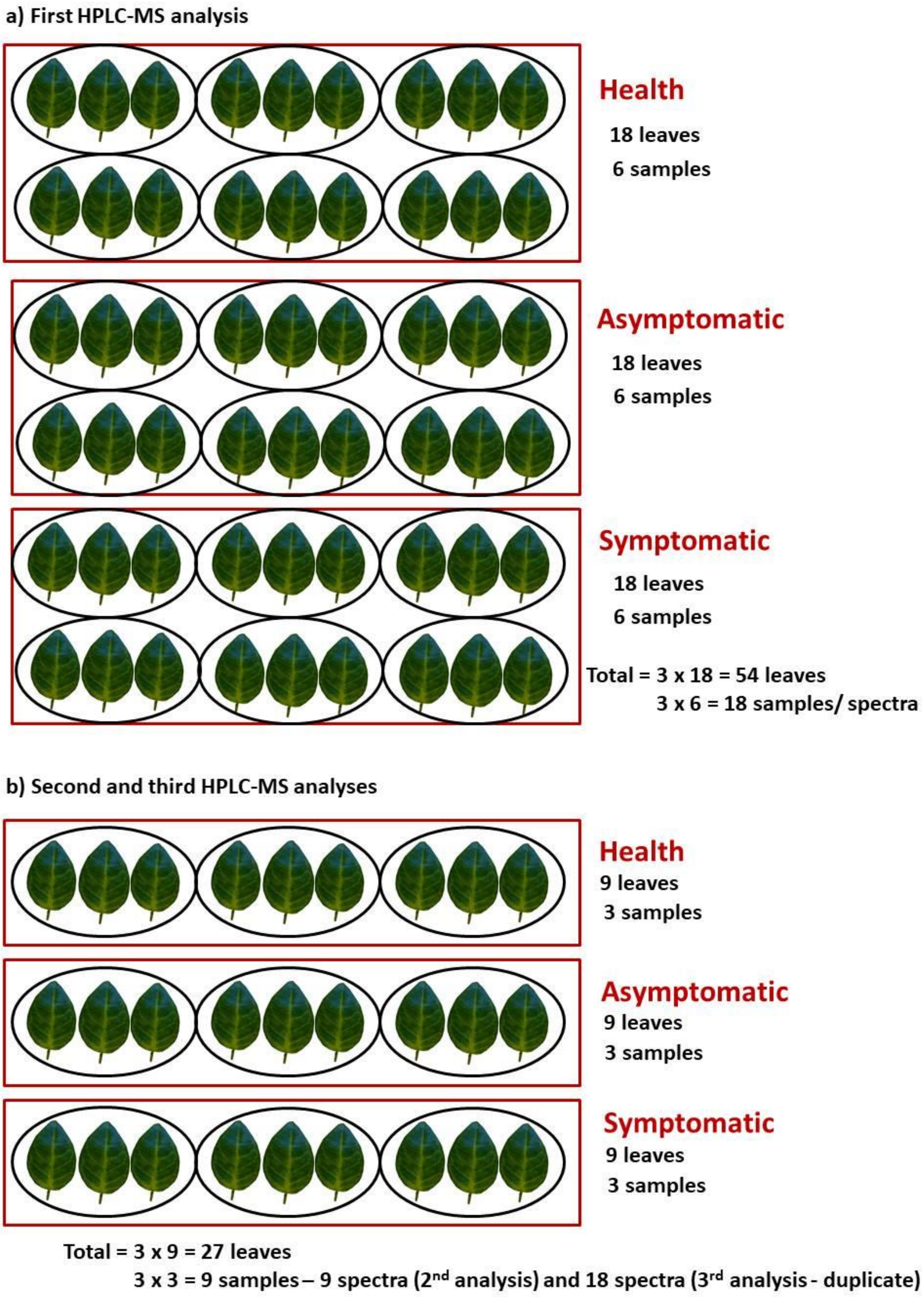

Figure S9. Reproducibility of HPLC-MS analyses. **a)** First HPLC-MS analysis performed with 18 leaves of each group, been 6 samples per group, totalizing 54 leaves and 18 spectra; **b)** Second and third HPLC-MS analyses performed with 9 leaves of each group, been 3 samples per group, totalizing 27 leaves, 9 spectra in the second analysis and 18 spectra in third analyses.

Abieta-8,11,13-trien-18-oic Acid ($C_{20}H_{28}O_2$)

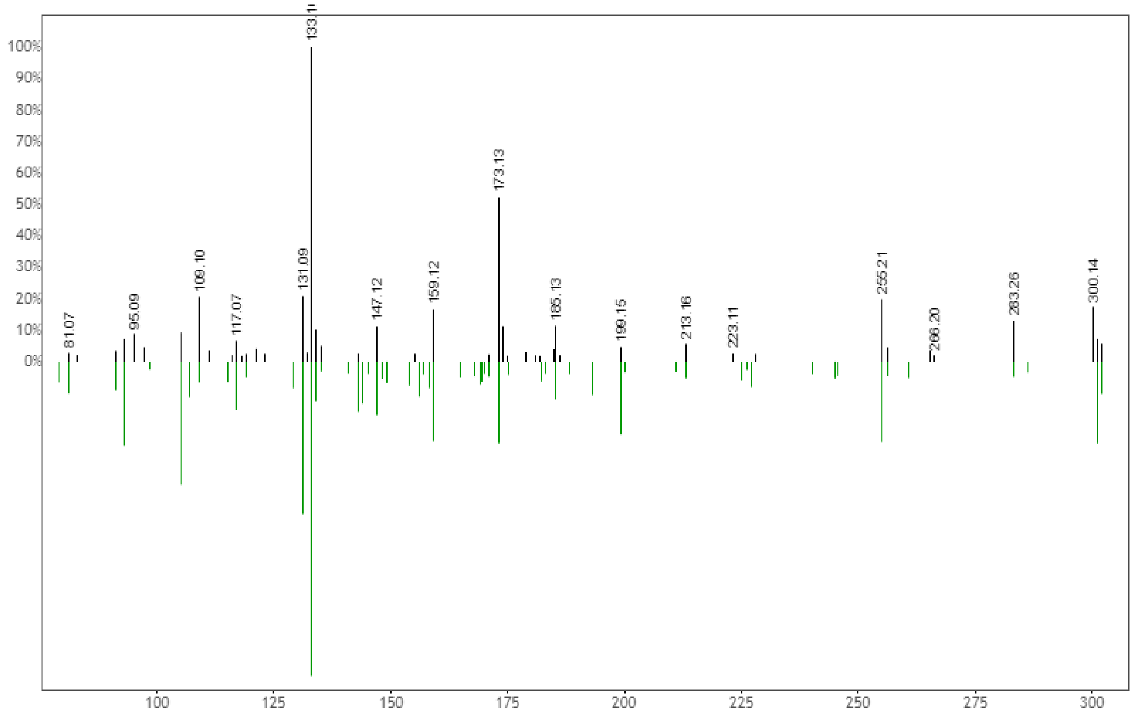

Figure S10. Mirror Match of GNPS to abieta-8,11,13-trien-18-oic acid in positive mode with Gold classification in Library Class of asymptomatic and symptomatic leaf sample. Green data are *m/z* values of GNPS Library and black are experimental data.

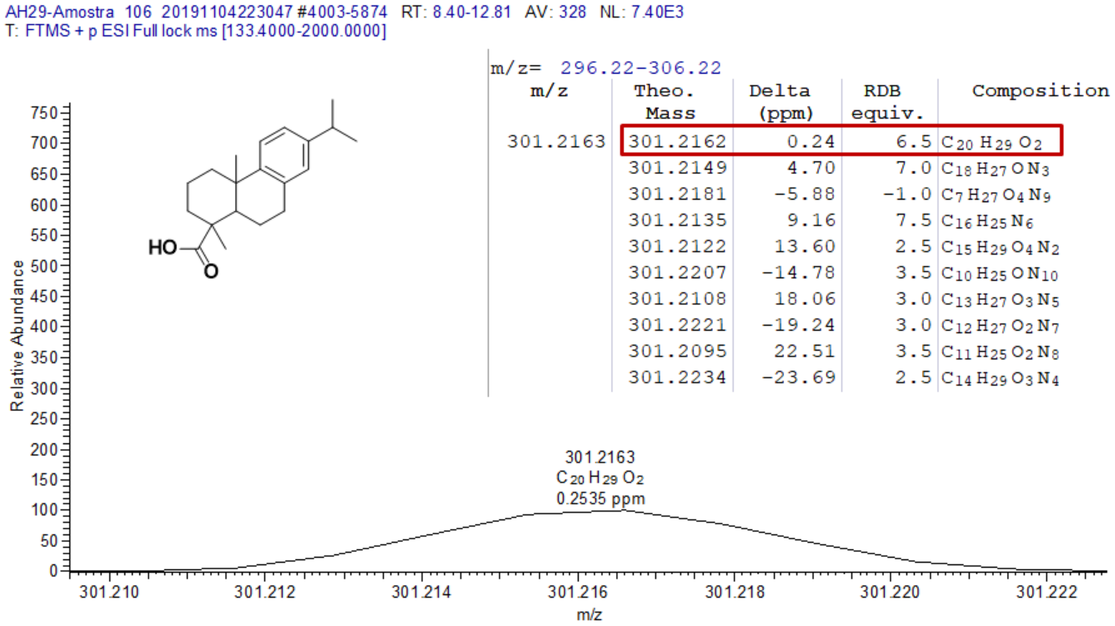

Figure S11. Mass spectrum - LC-MS in positive mode of leaf samples from *Citrus sinensis*. Assignment of *m/z* 301.2162 to abieta-8,11,13-trien-18-oic acid.

Abieta-8,11,13-trien-18-oic acid ($C_{20}H_{28}O_2$)

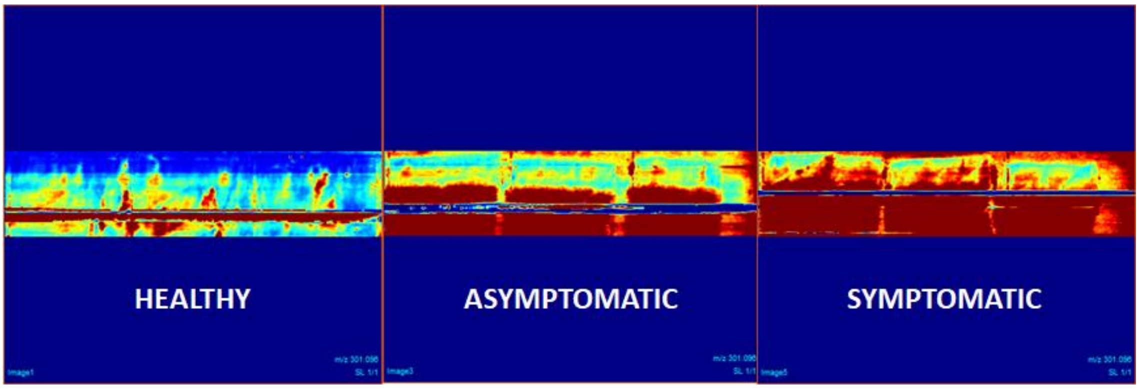

Figure S12. Image obtained by Mass Spectrometry Imaging (MSI) in positive mode of leaf samples from *Citrus sinensis* for abieta-8,11,13-trien-18-oic acid produced in different conditions.

Abscisic acid ($C_{15}H_{20}O_4$)

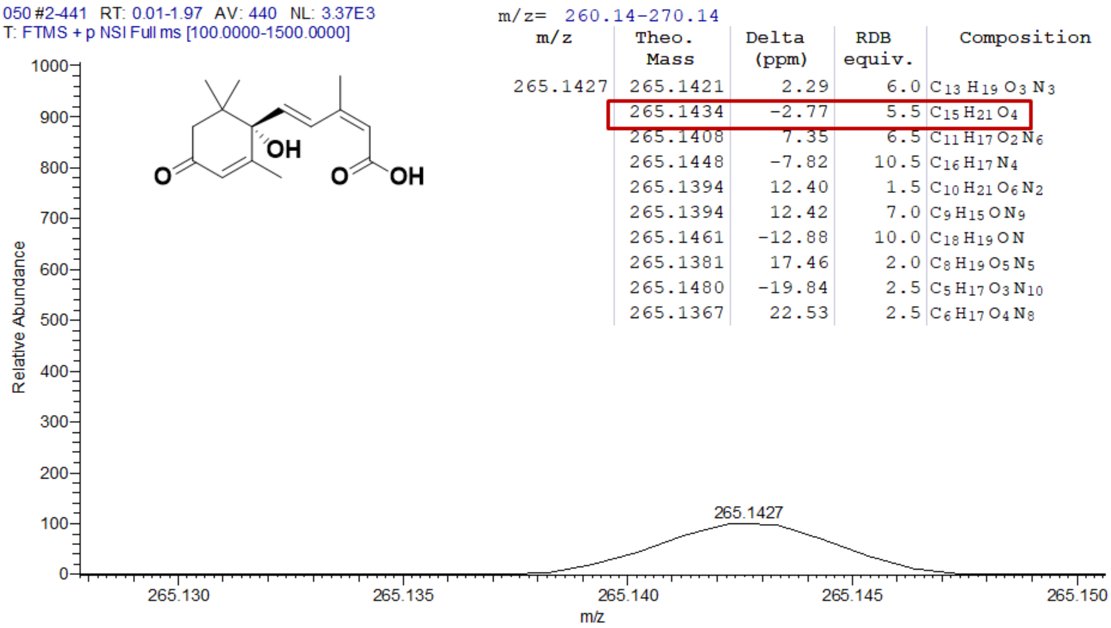

Figure S13. Mass spectrum - LC-MS in positive mode of leaf samples from *Citrus sinensis*. Assignment of *m/z* 265.1434 to abscisic acid.

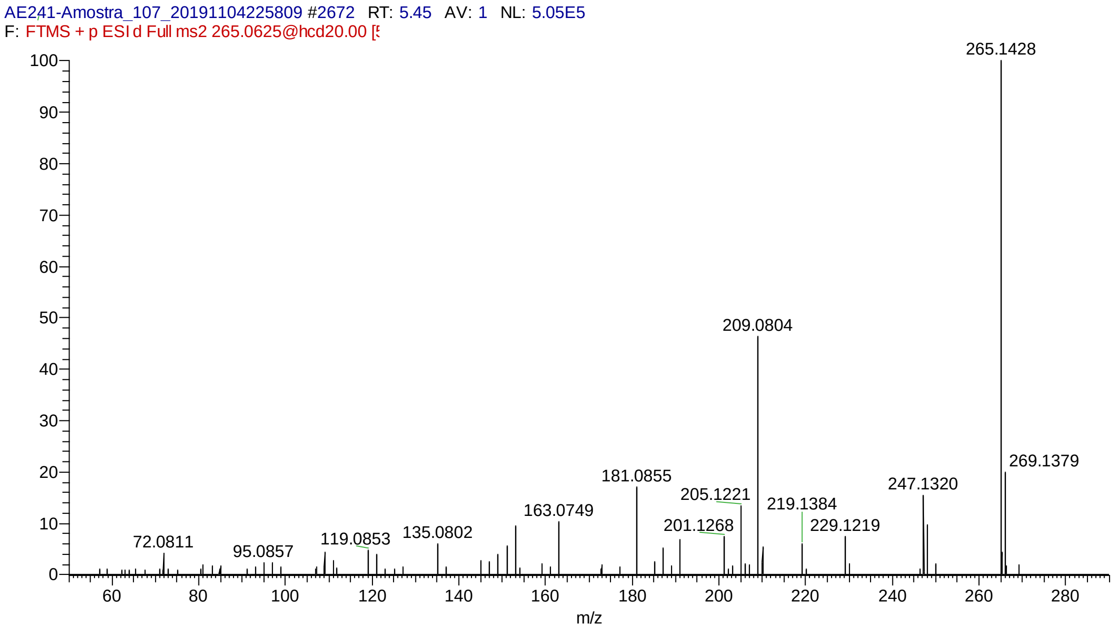

Figure S14. LC-MS/MS in positive mode (*m/z* 265.0625) of leaf samples from *Citrus sinensis*.

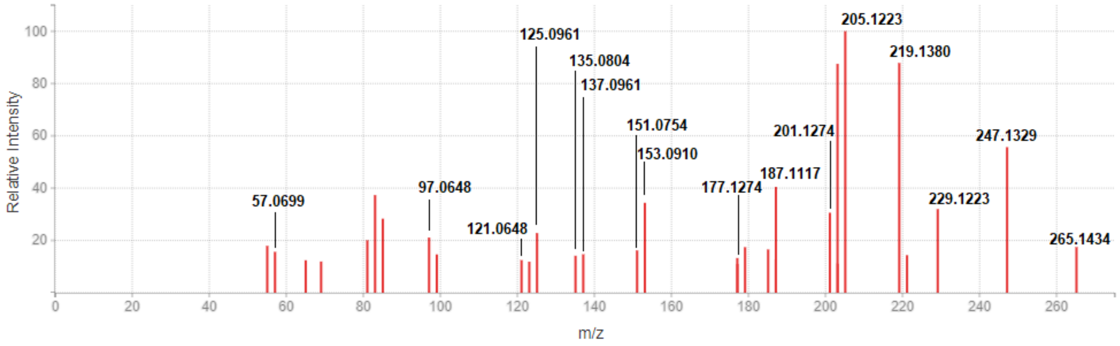

Figure S15. Predicted LC-MS/MS spectrum in positive mode of abscisic acid available in Human Metabolome Database (http://www.hmdb.ca/spectra/ms_ms/323747).

Comparison between experimental *m/z* values of LC-MS/MS and database to abscisic acid in positive mode

Database *m/z* (HMDB) predicted spectrum	Database *m/z* (GNPS) deposited spectrum	Experimental *m/z*	Error (ppm)	Formula Xcalibur
265.1434	265.00	265.1428	-2.44	$C_{15}H_{21}O_4$
247.1329	247.00	247.1320	-3.32	$C_{15}H_{19}O_3$
229.1223	229.00	229.1219	-1.64	$C_{15}H_{17}O_2$
219.1380	---	219.1384	2.16	$C_{14}H_{19}O_2$
205.1223	---	205.1221	-1.20	$C_{13}H_{17}O_2$
201.1274	---	201.1268	-3.09	$C_{14}H_{17}O$
187.1117	---	187.1111	-3.27	$C_{13}H_{15}O$
177.1274	---	177.1269	-2.72	$C_{12}H_{17}O$
153.0910	---	153.0906	-2.52	$C_9H_{13}O_2$
151.0754	---	151.0750	-2.49	$C_9H_{11}O_2$
137.0961	---	137.0953	-5.63	$C_9H_{13}O$
135.0804	---	135.0802	-1.64	$C_9H_{11}O$
125.0961	---	125.0952	-7.05	$C_8H_{13}O$
121.0648	---	121.0644	-3.56	C_8H_9O
97.0648	---	97.0647	-0.84	C_6H_9O
57.0699	---	57.0704	8.99	C_4H_9

Abscisic acid ($C_{15}H_{20}O_4$)

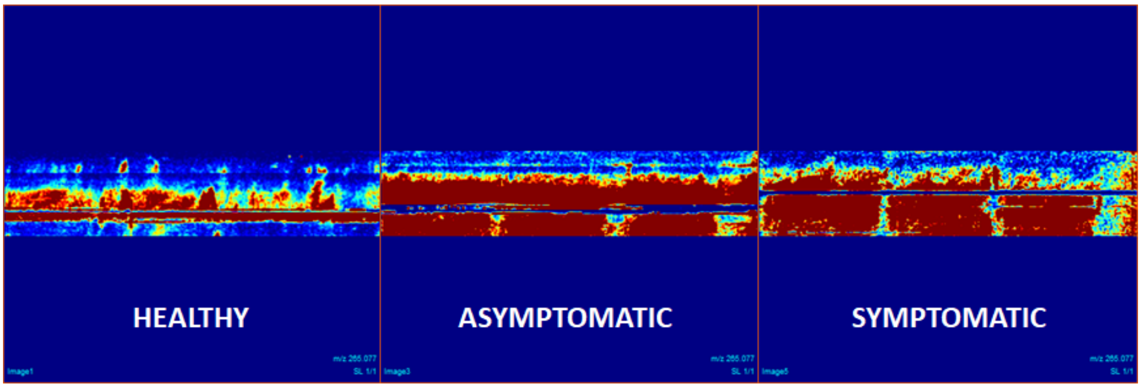

Figure S16. Image obtained by Mass Spectrometry Imaging (MSI) in positive mode of leaf samples from *Citrus sinensis* for abscisic acid produced in different conditions.

4-Acetyl-1-methylcyclohexene ($C_9H_{14}O$)

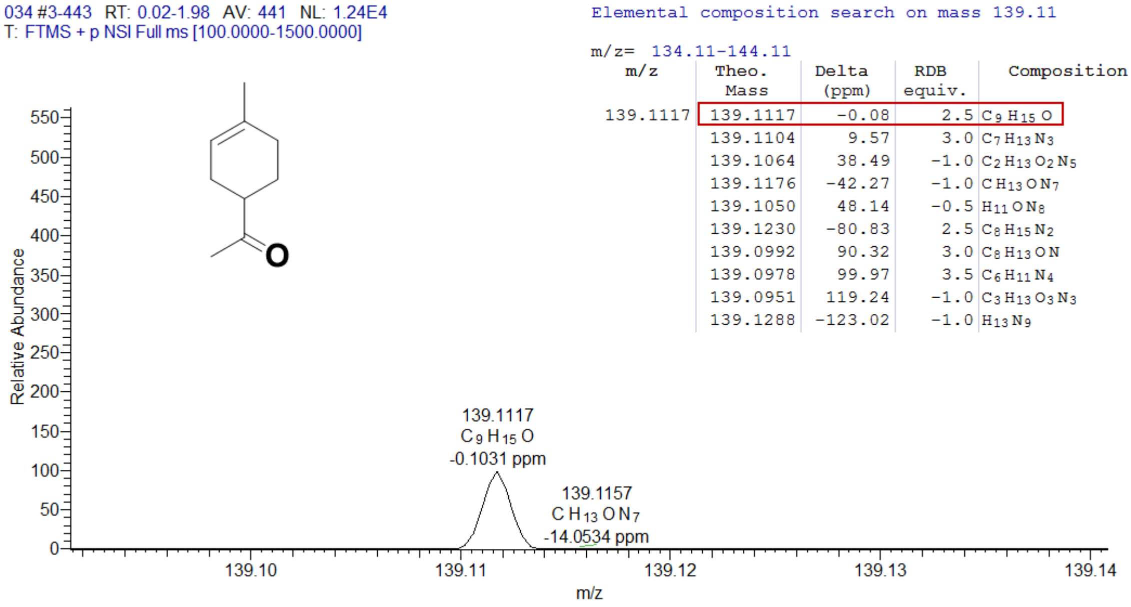

m/z	Theo. Mass	Delta (ppm)	RDB equiv.	Composition
139.1117	139.1117	-0.08	2.5	$C_9H_{15}O$
	139.1104	9.57	3.0	$C_7H_{13}N_3$
	139.1064	38.49	-1.0	$C_2H_{13}O_2N_5$
	139.1176	-42.27	-1.0	$CH_{13}ON_7$
	139.1050	48.14	-0.5	$H_{11}ON_8$
	139.1230	-80.83	2.5	$C_8H_{15}N_2$
	139.0992	90.32	3.0	$C_8H_{13}ON$
	139.0978	99.97	3.5	$C_6H_{11}N_4$
	139.0951	119.24	-1.0	$C_3H_{13}O_3N_3$
	139.1288	-123.02	-1.0	$H_{13}N_9$

Figure S17. Mass spectrum - LC-MS in positive mode of leaf samples from *Citrus sinensis*. Assignment of *m/z* 139.0502 to 4-acetyl-1-methylcyclohexene.

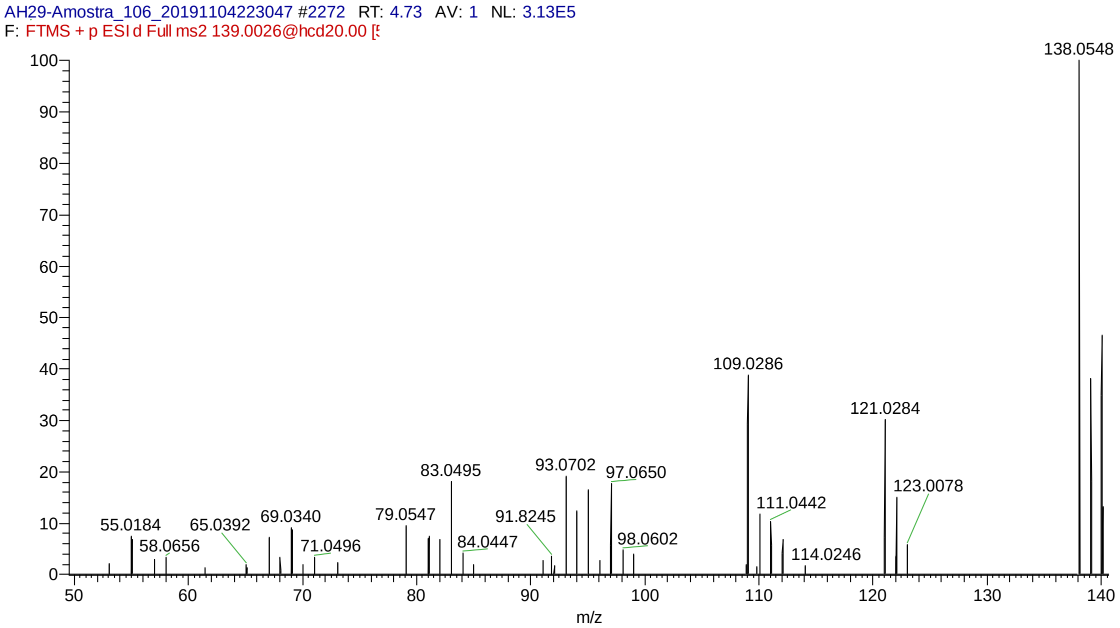

Figure S18. LC-MS/MS in positive mode (*m/z* 139.0026) of leaf samples from *Citrus sinensis*.

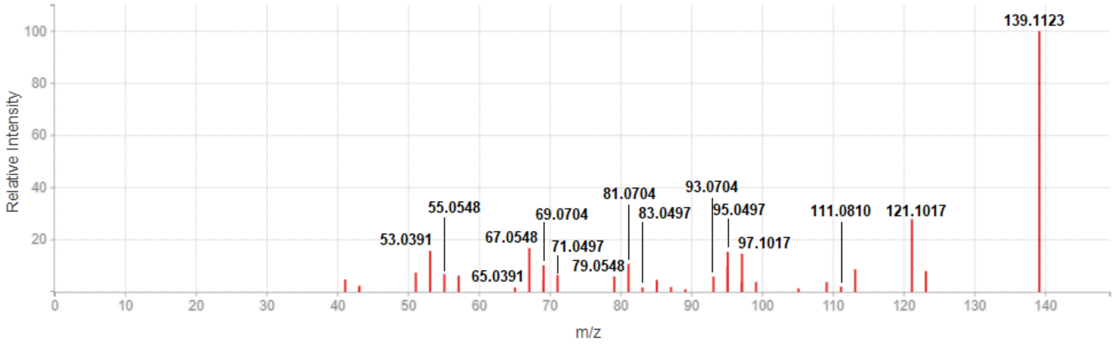

Figure S19. Predicted LC-MS/MS spectrum in positive mode of 4-acetyl-1-methylcyclohexene available in Human Metabolome Database (http://www.hmdb.ca/spectra/ms_ms/84589).

Comparison between experimental *m/z* values of LC-MS/MS and database to 4-acetyl-1-methylcyclohexene in positive mode

Database *m/z* (HMDB)	Experimental *m/z*	Error (ppm)	Formula Xcalibur
139.1123	139.1116	-1.02	$C_9H_{15}O$
121.1017	121.1013	0.69	C_9H_{13}
111.0810	111.0803	-1.00	$C_7H_{11}O$
97.1017	97.1017	5.39	C_7H_{13}
95.0497	95.0495	3.35	C_6H_7O
93.0704	93.0702	3.04	C_7H_9
83.0497	83.0495	4.56	C_5H_7O
81.0704	81.0702	4.48	C_6H_9
79.0548	79.0547	6.24	C_6H_7
71.0497	71.0496	7.02	C_4H_7O
69.0704	69.0707	11.34	C_5H_9
67.0548	67.0547	6.91	C_5H_7
65.0391	65.0392	8.97	C_5H_5
55.0548	55.0547	8.78	C_4H_7
53.0391	53.0389	5.15	C_4H_5

4-Acetyl-1-methylcyclohexene ($C_9H_{14}O$)

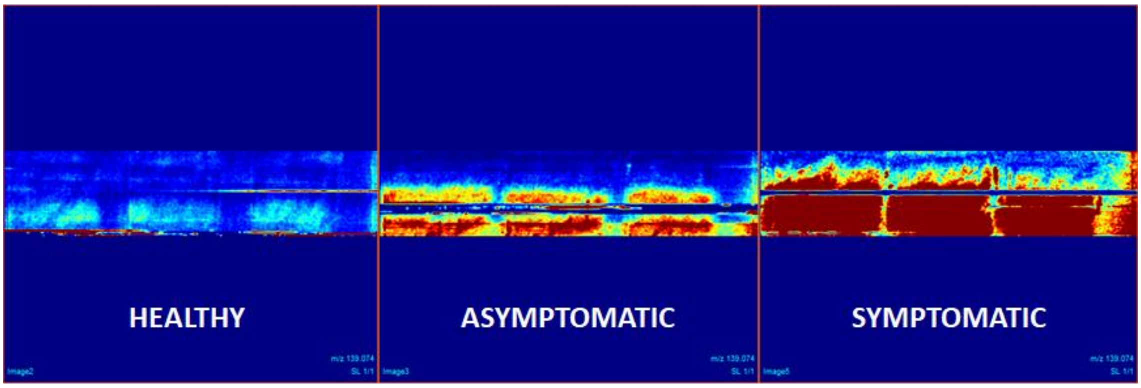

Figure S20. Image obtained by Mass Spectrometry Imaging (MSI) in positive mode of leaf samples from *Citrus sinensis* for 4-Acetyl-1-methylcyclohexene produced in different conditions.

Asparagine ($C_4H_8N_2O_3$)

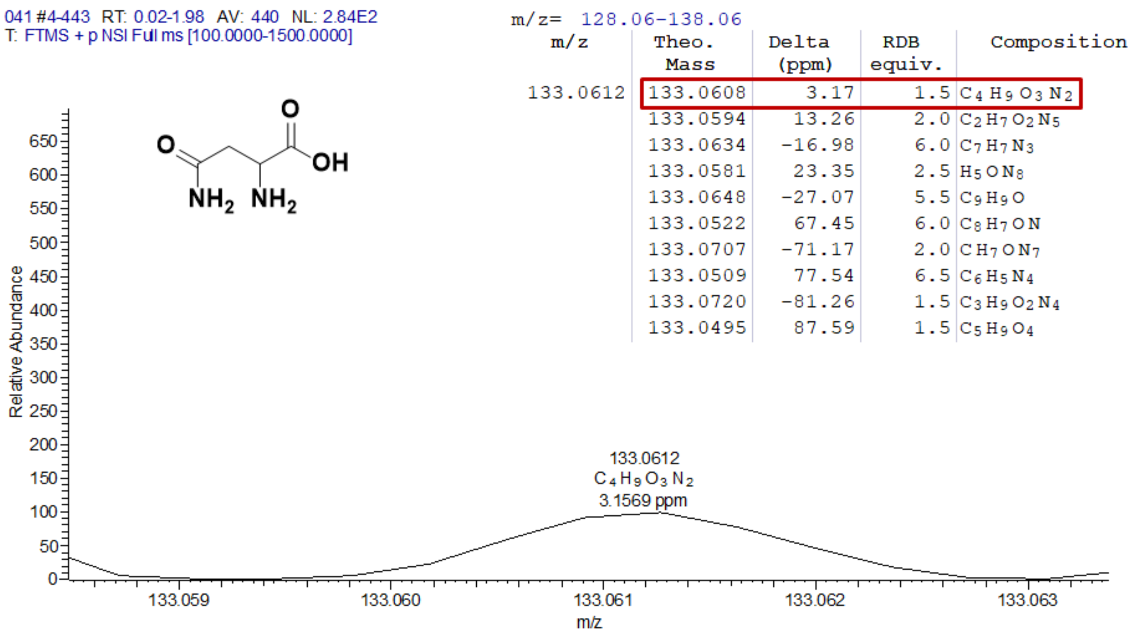

m/z	Theo. Mass	Delta (ppm)	RDB equiv.	Composition
133.0612	133.0608	3.17	1.5	$C_4H_9O_3N_2$
	133.0594	13.26	2.0	$C_2H_7O_2N_5$
	133.0634	-16.98	6.0	$C_7H_7N_3$
	133.0581	23.35	2.5	H_5ON_8
	133.0648	-27.07	5.5	C_9H_9O
	133.0522	67.45	6.0	C_8H_7ON
	133.0707	-71.17	2.0	CH_7ON_7
	133.0509	77.54	6.5	$C_6H_5N_4$
	133.0720	-81.26	1.5	$C_3H_9O_2N_4$
	133.0495	87.59	1.5	$C_5H_9O_4$

Figure S21. Mass spectrum - LC-MS in positive mode of leaf samples from *Citrus sinensis*. Assignment of *m/z* 133.0608 to asparagine.

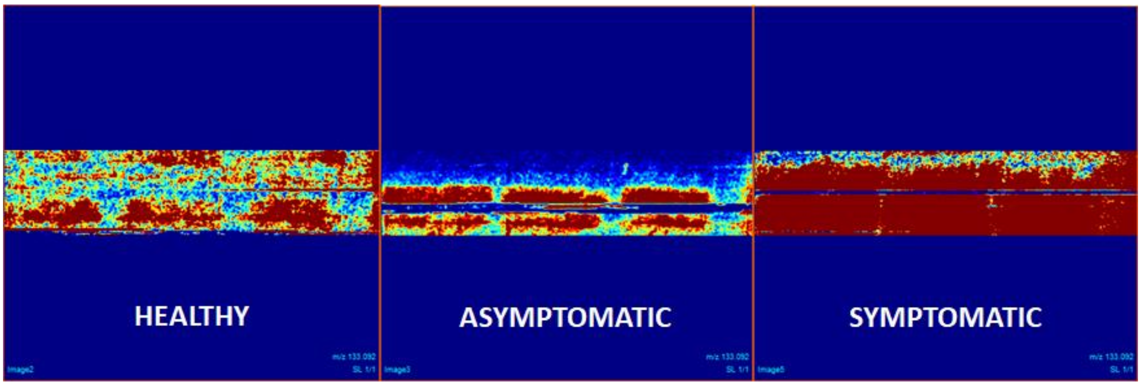

Figure S22. Image obtained by Mass Spectrometry Imaging (MSI) in positive mode of leaf samples from *Citrus sinensis* for asparagine produced in different conditions.

Asparagine ($C_4H_8N_2O_3$)

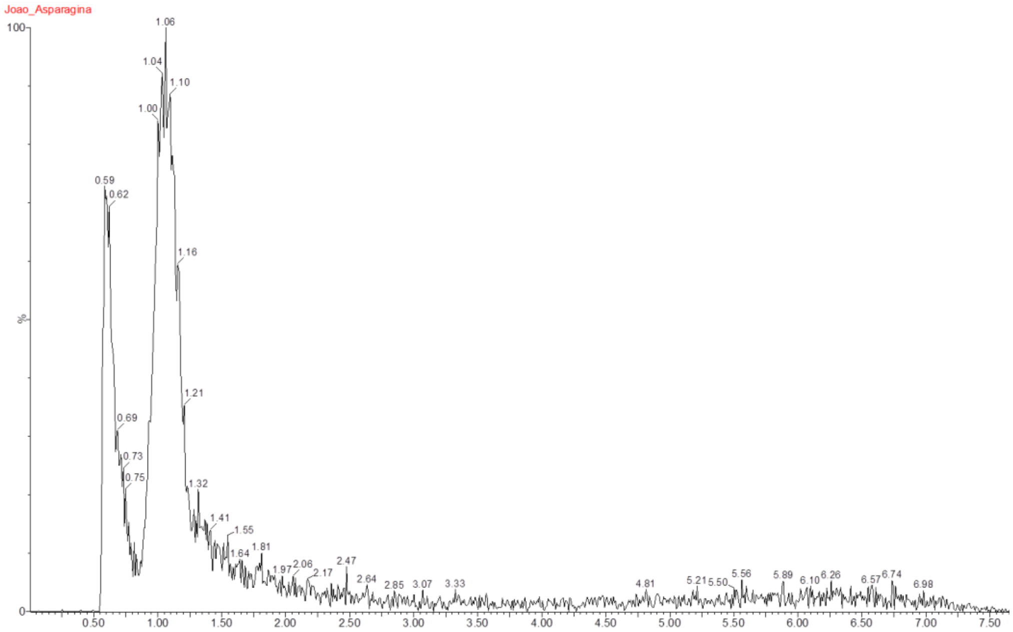

Figure S23. Selected ion chromatogram (*m/z* 133) of a standard sample of asparagine.

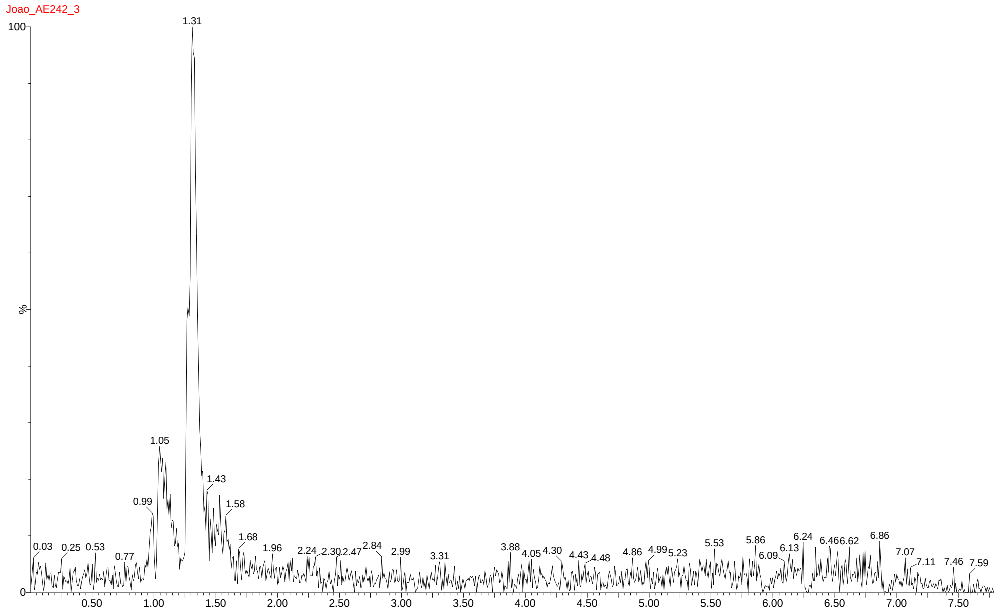

Figure S24. Selected ion chromatogram (*m/z* 133) of asymptomatic leaves sample.

Feruloylputrescine ($C_{14}H_{20}N_2O_3$)

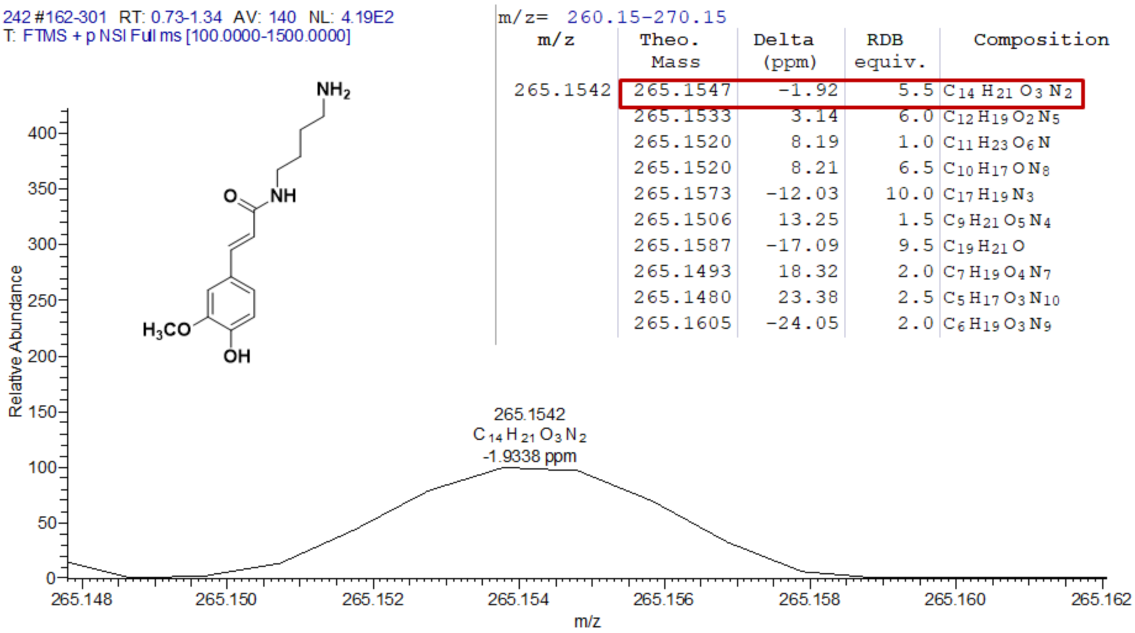

Figure S25. Mass spectrum - LC-MS in positive mode of leaf samples from *Citrus sinensis*. Assignment of *m/z* 265.1547 to feruloylputrescine.

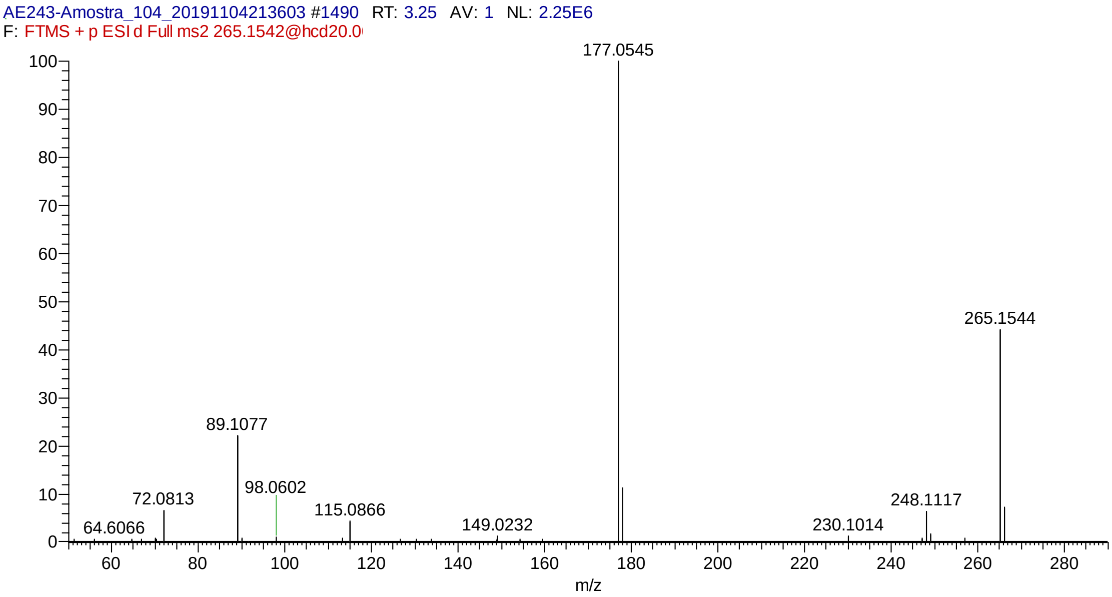

Figure S26. LC-MS/MS in positive mode (*m/z* 265.1542) of leaf samples from *Citrus sinensis*.

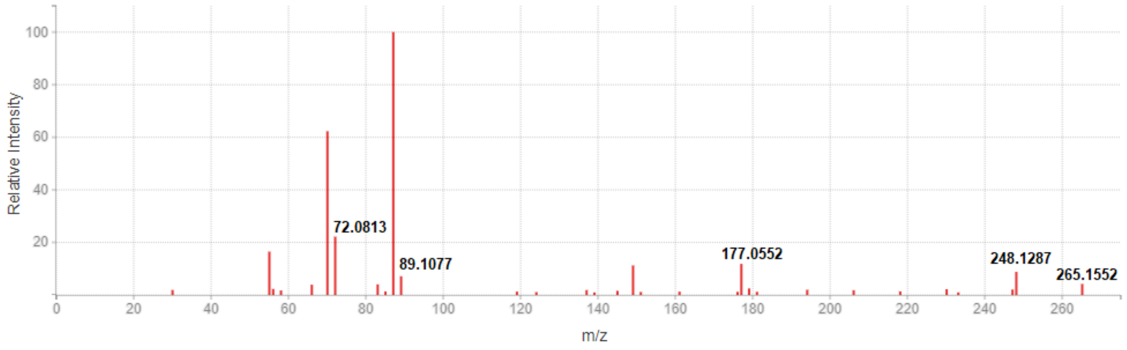

Figure S27. Predicted LC-MS/MS spectrum in positive mode of feruloylputrescine available in Human Metabolome Database (http://www.hmdb.ca/spectra/ms_ms/55360).

Comparison between experimental *m/z* values of LC-MS/MS and database to feruloylputrescine in positive mode

Database *m/z* (HMDB)	Experimental *m/z*	Error (ppm)	Formula Xcalibur
265.1552	265.1544	-1.05	$C_{14}H_{21}N_2O_3$
248.1287	248.1297	6.57	$C_{14}H_{18}NO_3$
177.0552	177.0545	-1.19	$C_{10}H_9O_3$
89.1077	89.1079	3.76	$C_4H_{13}N_2$
72.0813	72.0813	6.99	$C_4H_{10}N$

Feruloylputrescine ($C_{14}H_{20}N_2O_3$)

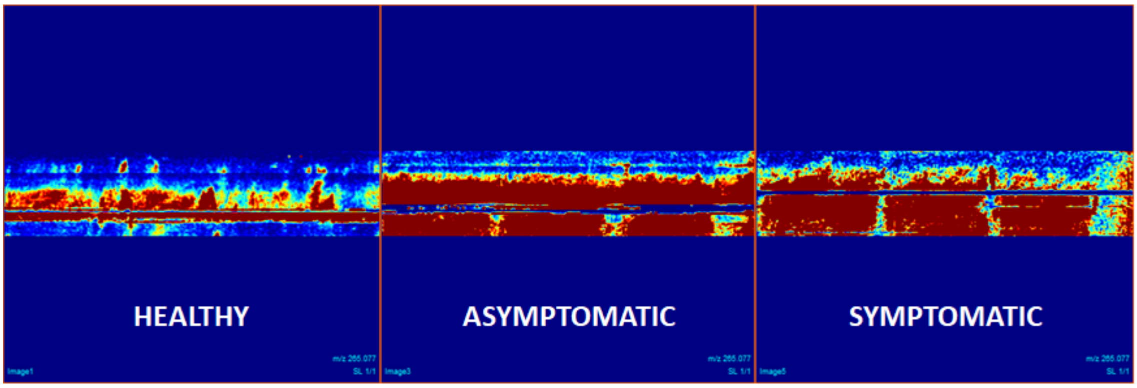

Figure S28. Image obtained by Mass Spectrometry Imaging (MSI) in positive mode of leaf samples from *Citrus sinensis* for feruloylputrescine produced in different conditions.

β-Glucose ($C_6H_{12}O_6$)

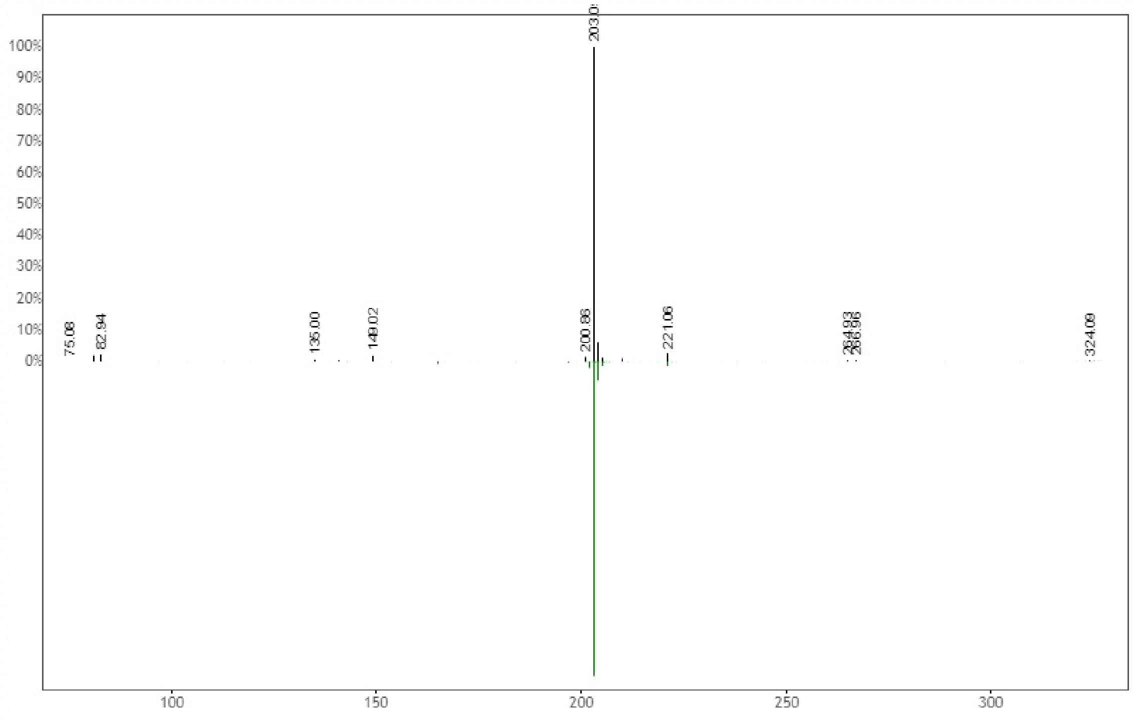

Figure S29. Mirror Match of GNPS to β-glucose in positive mode with Bronze classification in Library Class of asymptomatic leaf sample. Green data are *m/z* values of GNPS Library and black are experimental data.

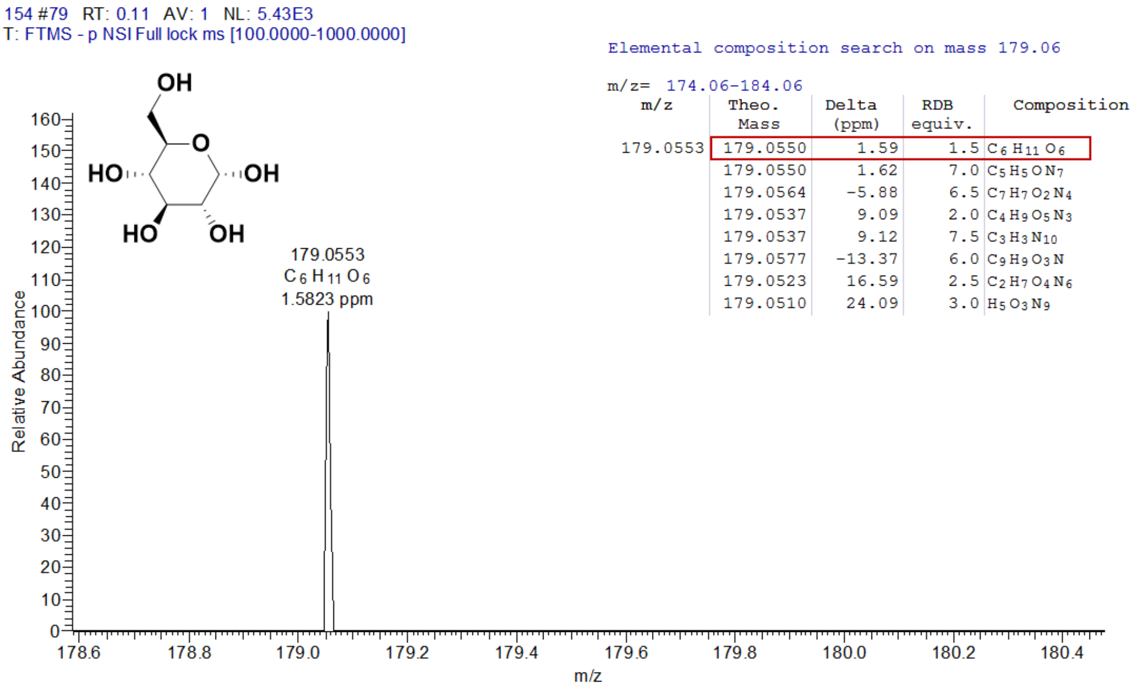

m/z	Theo. Mass	Delta (ppm)	RDB equiv.	Composition
179.0553	179.0550	1.59	1.5	$C_6H_{11}O_6$
	179.0550	1.62	7.0	$C_5H_5ON_7$
	179.0564	-5.88	6.5	$C_7H_7O_2N_4$
	179.0537	9.09	2.0	$C_4H_9O_5N_3$
	179.0537	9.12	7.5	$C_3H_3N_{10}$
	179.0577	-13.37	6.0	$C_9H_9O_3N$
	179.0523	16.59	2.5	$C_2H_7O_4N_6$
	179.0510	24.09	3.0	$H_5O_3N_9$

Figure S30. Mass spectrum - LC-MS in negative mode of leaf samples from *Citrus sinensis*. Assignment of *m/z* 179.0550 to β-glucose.

β-Glucose ($C_6H_{12}O_6$)

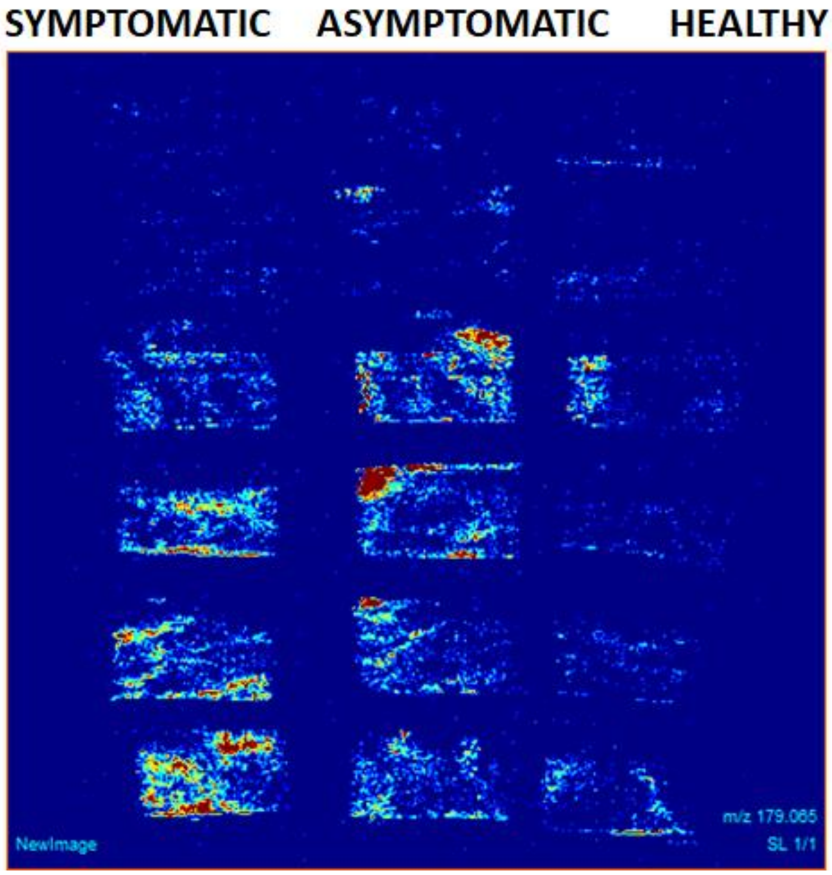

Figure S31. Image obtained by Mass Spectrometry Imaging (MSI) in negative mode of leaf samples from *Citrus sinensis* for glucose produced in different conditions.

Guaiacol ($C_7H_8O_2$)

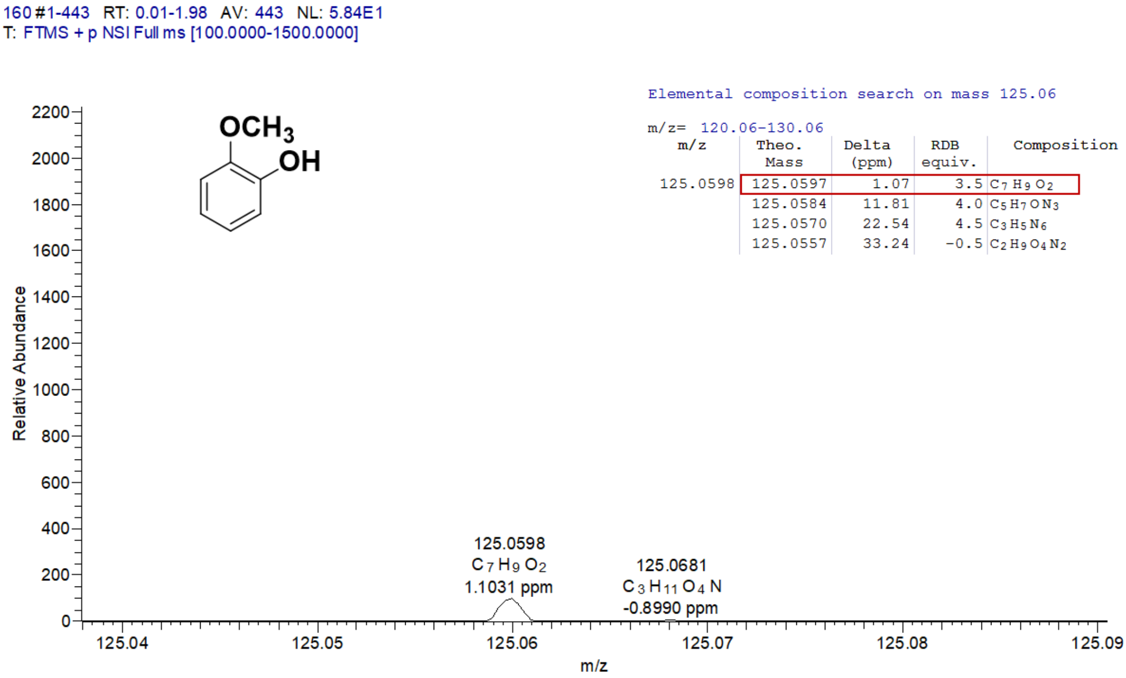

Figure S32. Mass spectrum - LC-MS in positive mode of leaf samples from *Citrus sinensis*. Assignment of *m/z* 125.0597 to guaiacol.

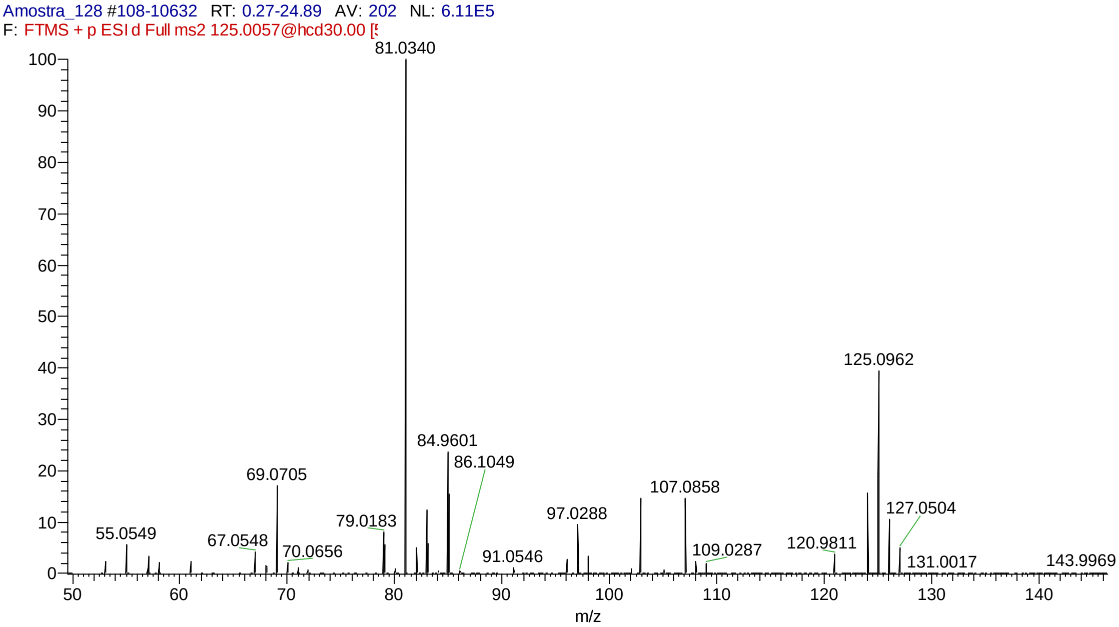

Figure S33. LC-MS/MS in positive mode (*m/z* 125.0057) of leaf samples from *Citrus sinensis*.

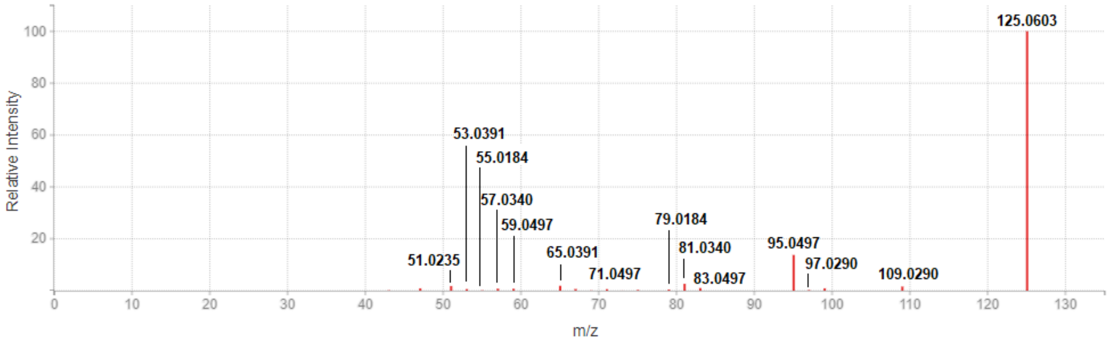

Figure S34. Predicted LC-MS/MS spectrum in positive mode of guaiacol available in Human Metabolome Database (http://www.hmdb.ca/spectra/ms_ms/7242).

Comparison between experimental *m/z* values and database to guaiacol in positive mode

Database *m/z* (HMDB)	Experimental *m/z*	Error (ppm)	Formula Xcalibur
125.0603	125.0599	1.47	$C_7H_9O_2$
109.0290	109.0287	3.06	$C_6H_5O_2$
97.0290	97.0288	4.06	$C_5H_5O_2$
95.0497	95.0495	3.67	C_6H_7O
83.0497	83.0496	5.88	C_5H_7O
81.0340	81.0340	6.28	C_5H_5O
79.0184	79.0183	6.31	C_5H_3O
71.0497	71.0498	8.57	C_4H_7O
65.0391	65.0392	8.97	C_5H_5
59.0497	59.0497	9.63	C_3H_7O
57.0340	57.0341	10.67	C_3H_5O
55.0184	55.0185	11.06	C_3H_3O
53.0391	53.0392	12.13	C_4H_5
51.0235	51.0238	16.53	C_4H_3

Guaiacol ($C_7H_8O_2$)

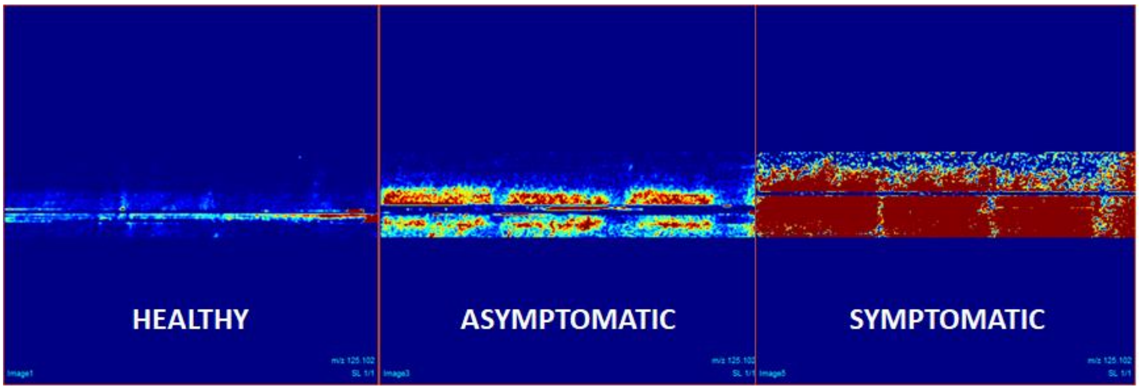

Figure S35. Image obtained by Mass Spectrometry Imaging (MSI) in positive mode of leaf samples from *Citrus sinensis* for guaiacol produced in different conditions.

p-Hydroxycinnamic acid ($C_9H_8O_3$)

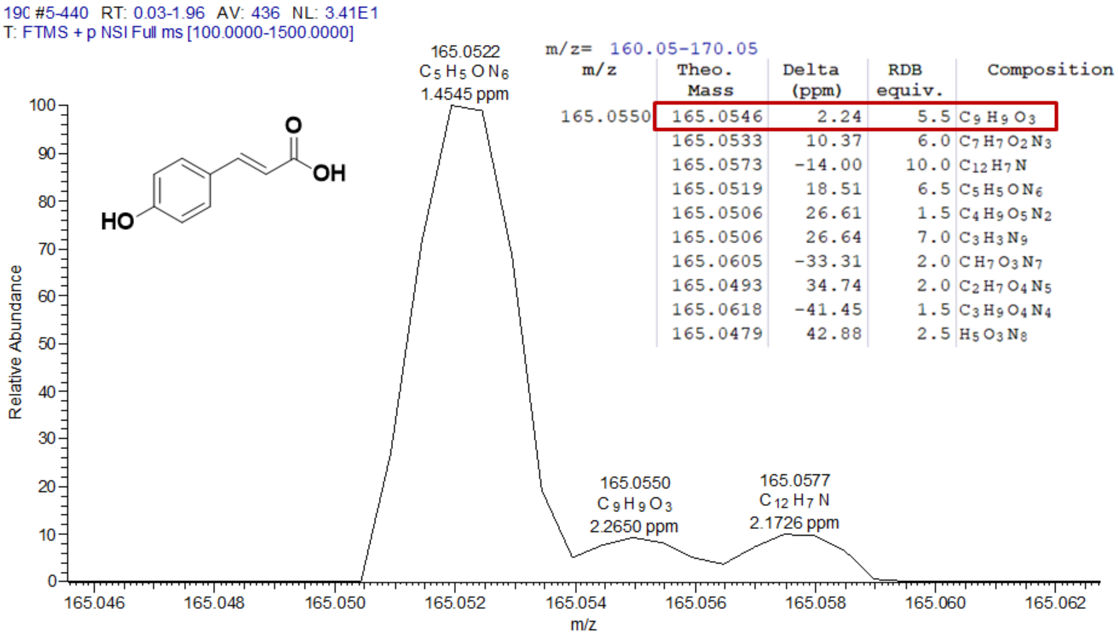

Figure S36. Mass spectrum - LC-MS in positive mode of leaf samples from *Citrus sinensis*. Assignment of *m/z* 165.0546 to *p*-hydroxycinnamic acid.

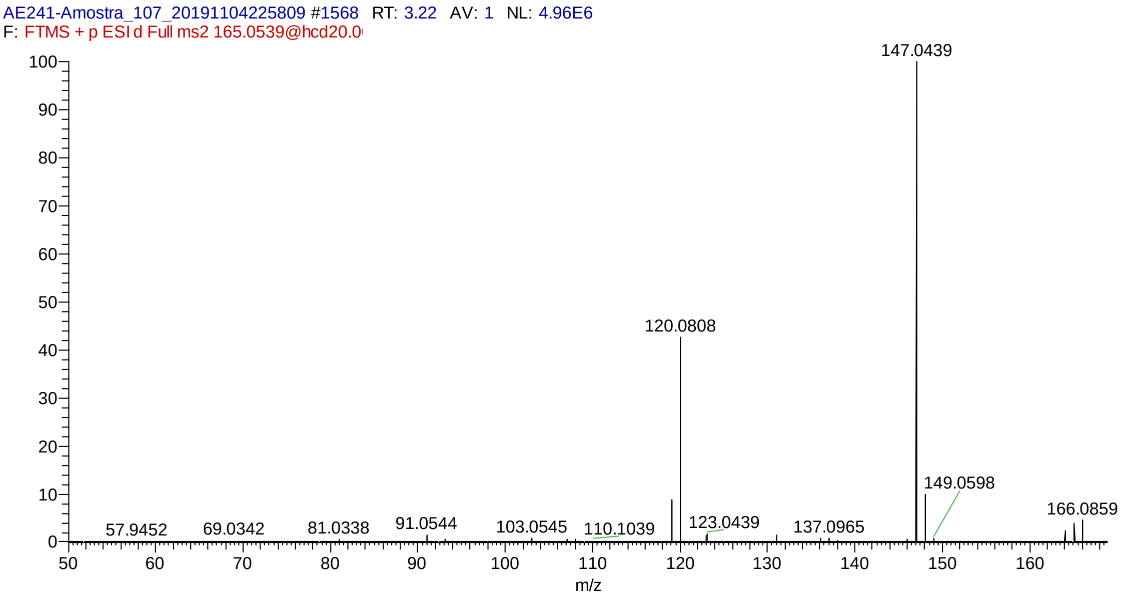

Figure S37. LC-MS/MS in positive mode (*m/z* 165.0539) of leaf samples from *Citrus sinensis*.

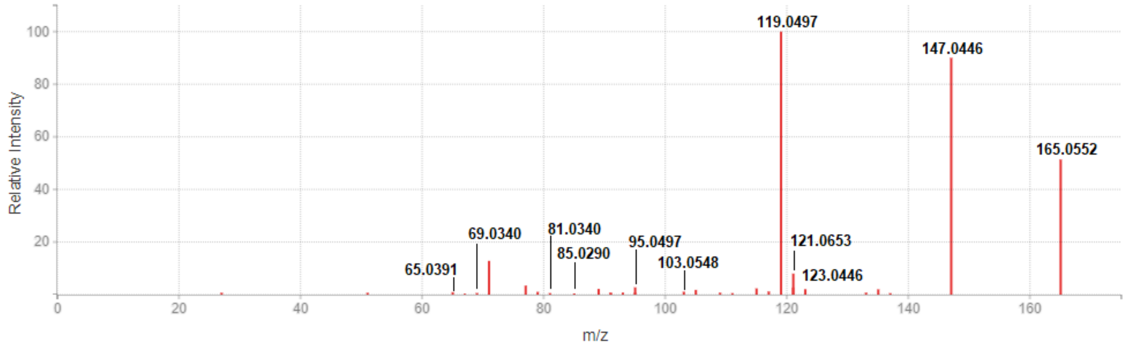

Figure S38. Predicted LC-MS/MS spectrum in positive mode of *p*-hydroxycinnamic acid available in Human Metabolome Database (http://www.hmdb.ca/spectra/ms_ms/179818).

Comparison between experimental *m/z* values of LC-MS/MS and database to *p*-hydroxycinnamic acid in positive mode

Database *m/z* (HMDB) predicted spectrum	Database *m/z* (GNPS) deposited spectrum	Experimental *m/z*	Error (ppm)	Formula Xcalibur
165.0552	165.06	165.0547	0.48	$C_9H_9O_3$
147.0446	147.04	147.0439	-1.40	$C_9H_7O_2$
123.0446	---	123.0439	-1.19	$C_7H_7O_2$
121.0653	---	121.0647	-0.43	C_8H_9O
119.0497	119.05	119.0492	0.32	C_8H_7O
103.0548	---	103.0545	3.04	C_8H_7
95.0497	---	95.0492	0.20	C_6H_7O
85.0290	---	85.0287	3.11	$C_4H_5O_2$
81.0340	---	81.0338	4.06	C_5H_5O
69.0340	---	69.0342	10.12	C_4H_5O
65.0391	65.04	65.0393	10.97	C_5H_5

p-Hydroxycinnamic acid ($C_9H_8O_3$)

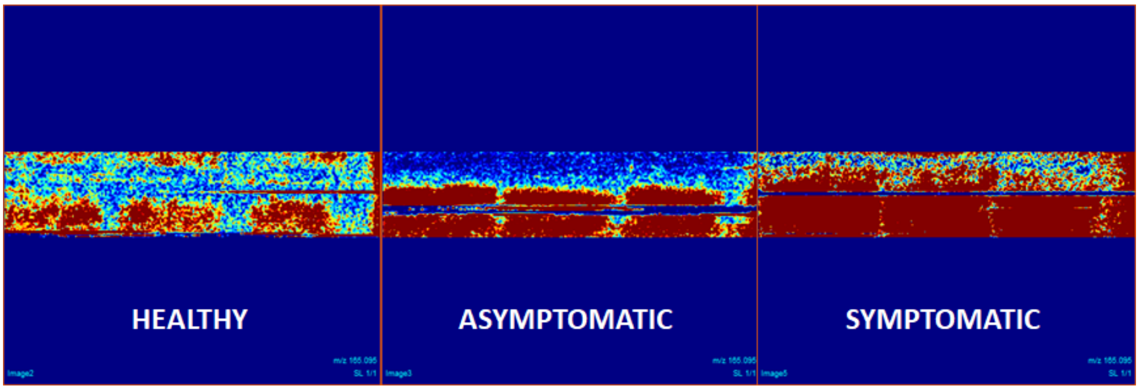

Figure S39. Image obtained by Mass Spectrometry Imaging (MSI) in positive mode of leaf samples from *Citrus sinensis* for *p*-hydroxycinnamic acid produced in different conditions.

Isoleucine ($C_6H_{13}NO_2$)

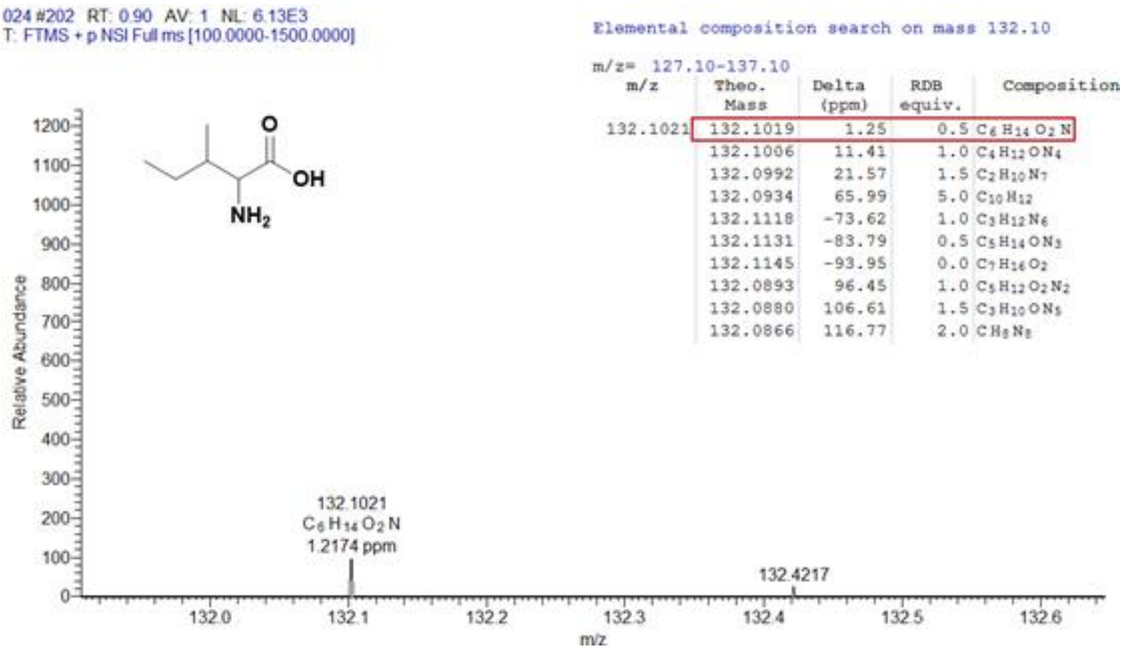

Figure S40. Mass spectrum - LC-MS in positive mode of leaf samples from *Citrus sinensis*. Assignment of *m/z* 132.1019 to isoleucine.

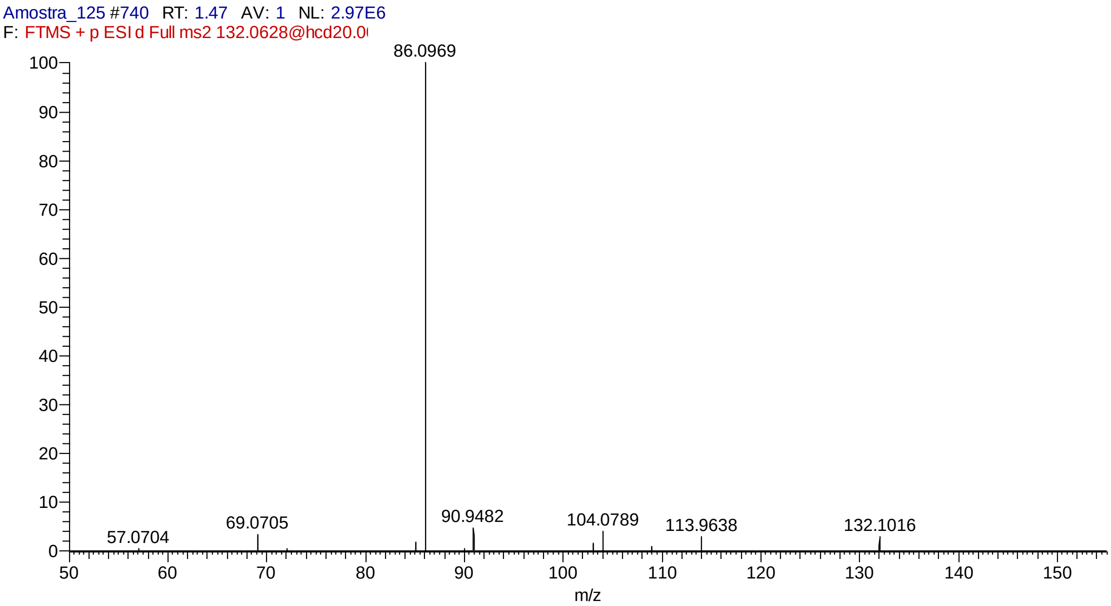

Figure S41. LC-MS/MS in positive mode (*m/z* 132.0628) of leaf samples from *Citrus sinensis*.

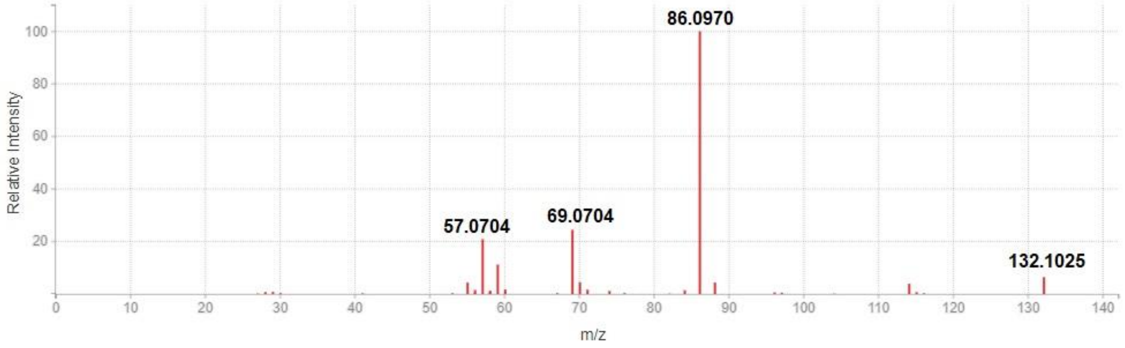

Figure S42. Predicted LC-MS/MS spectrum in positive mode of isoleucine available in Human Metabolome Database (https://hmdb.ca/spectra/ms_ms/178771).

Comparison between experimental *m/z* values of LC-MS/MS and database to isoleucine in positive mode

Database *m/z* (HMDB) predicted spectrum	Experimental *m/z*	Error (ppm)	Formula Xcalibur
132.1025	132.1019	-2.39	$C_6H_{14}NO_2$
86.0970	86.0964	5.27	$C_5H_{12}N$
69.0704	69.0699	8.59	C_5H_9
57.0704	57.0699	8.99	C_4H_9

Isoleucine ($C_6H_{13}NO_2$)

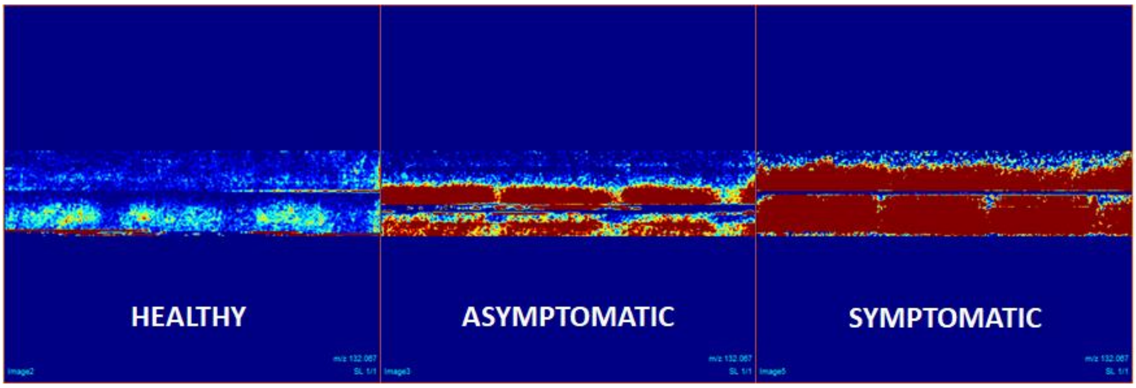

Figure S43. Image obtained by Mass Spectrometry Imaging (MSI) in positive mode of leaf samples from *Citrus sinensis* for isoleucine produced in different conditions.

trans-jasmonic acid ($C_{12}H_{18}O_3$)

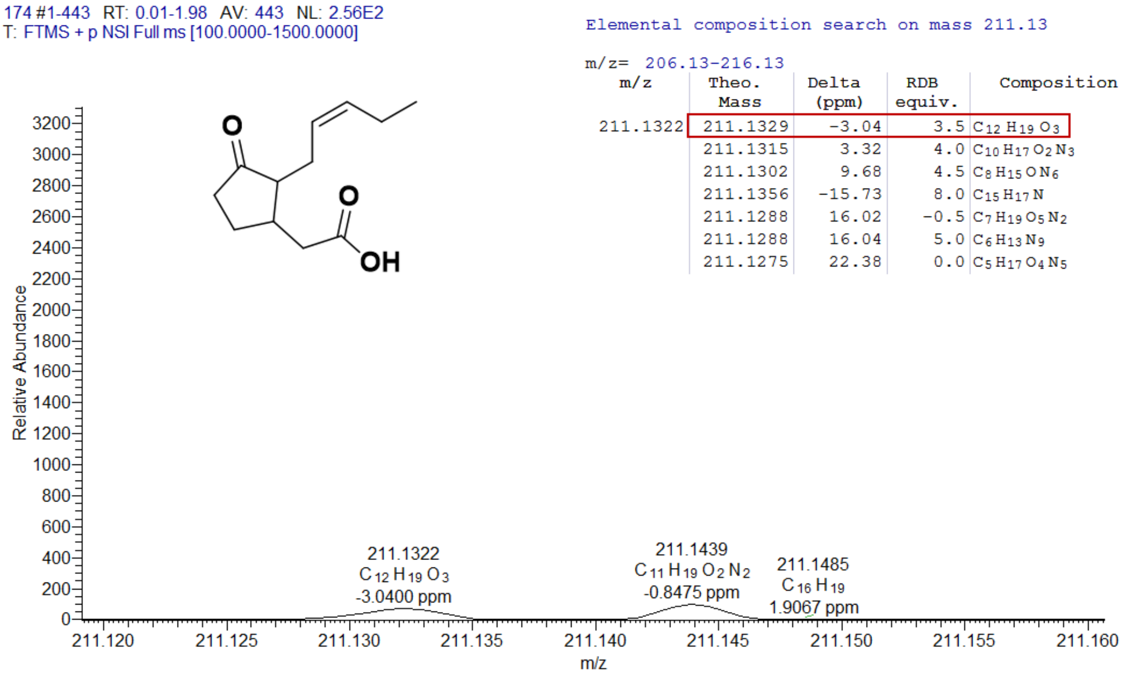

Figure S44. Mass spectrum - LC-MS in positive mode of leaf samples from *Citrus sinensis*. Assignment of *m/z* 211.1329 to *trans*-jasmonic acid.

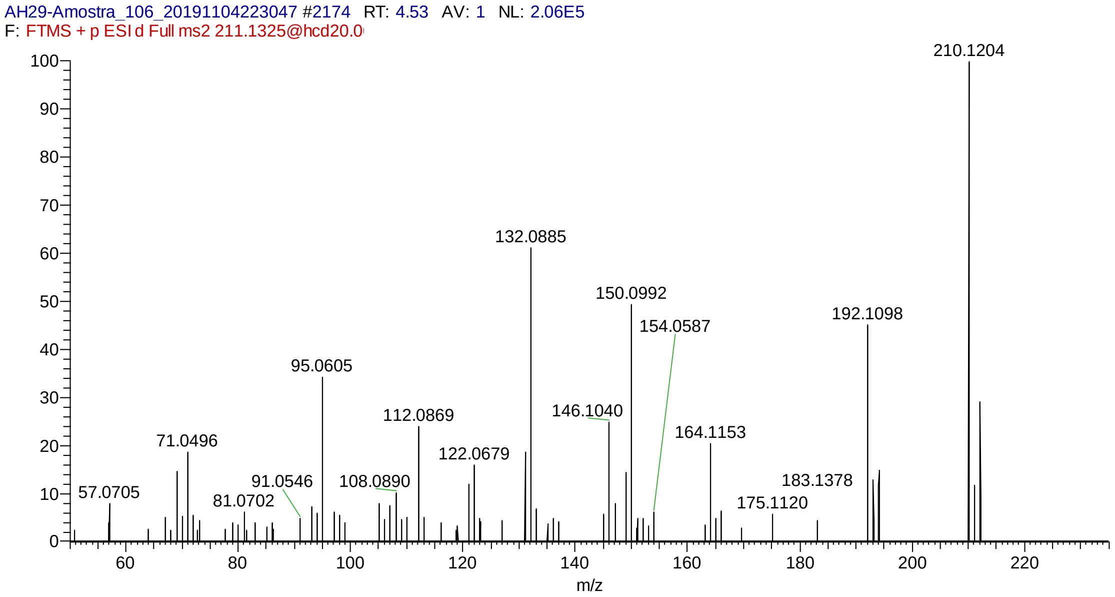

Figure S45. LC-MS/MS in positive mode (*m/z* 211.1325) of leaf samples from *Citrus sinensis*.

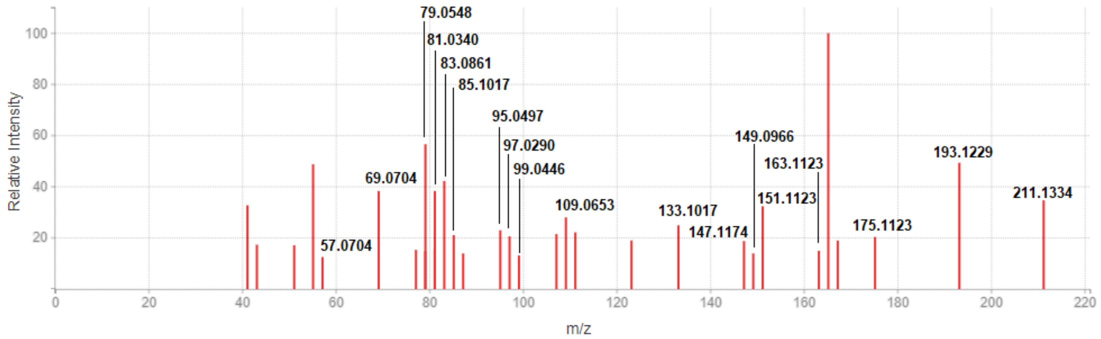

Figure S46. Predicted LC-MS/MS spectrum in positive mode of *trans*-jasmonic acid available in Human Metabolome Database (http://www.hmdb.ca/spectra/ms_ms/58216).

Comparison between experimental *m/z* values of LC-MS/MS and database to *trans*-jasmonic acid in positive mode

Database *m/z* (HMDB) predicted spectrum	Database *m/z* (GNPS) deposited spectrum	Experimental *m/z*	Error (ppm)	Formula Xcalibur
211.1334	211.13	211.1323	-2.89	$C_{12}H_{19}O_3$
193.1229	193.12	193.1228	2.71	$C_{12}H_{17}O_2$
175.1123	---	175.1120	1.30	$C_{12}H_{15}O$
163.1123	---	163.1120	1.65	$C_{11}H_{15}O$
151.1123	151.11	151.1125	5.15	$C_{10}H_{15}O$
149.0966	149.10	149.0956	-3.50	$C_{10}H_{13}O$
147.1174	147.11	147.1165	-2.09	$C_{11}H_{15}$
133.1017	133.10	133.1014	1.53	$C_{10}H_{13}$
109.0653	109.07	109.0649	1.27	C_7H_9O
99.0446	---	99.0442	1.55	$C_5H_7O_2$
97.0290	---	97.0285	1.28	$C_5H_5O_2$
95.0497	---	95.0494	2.93	C_6H_7O
85.1017	---	85.1013	1.45	C_6H_{13}
83.0861	---	83.0859	5.09	C_6H_{11}
81.0340	---	81.0339	4.55	C_5H_5O
79.0548	---	79.0546	4.47	C_6H_7
69.0704	---	69.0704	7.72	C_5H_9
57.0704	---	57.0705	10.74	C_4H_9

trans-jasmonic acid ($C_{12}H_{18}O_3$)

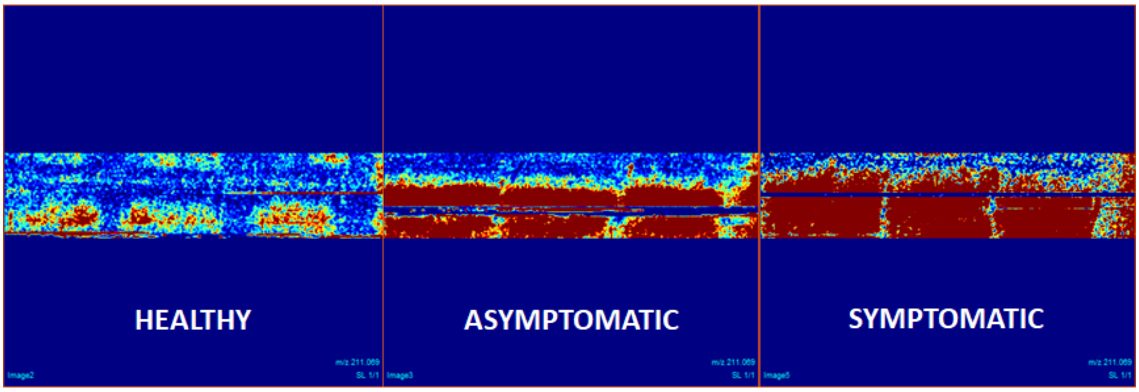

Figure S47. Image obtained by Mass Spectrometry Imaging (MSI) in positive mode of leaf samples from *Citrus sinensis* for *trans*-jasmonic acid produced in different conditions.

Nobiletin ($C_{21}H_{22}O_8$)

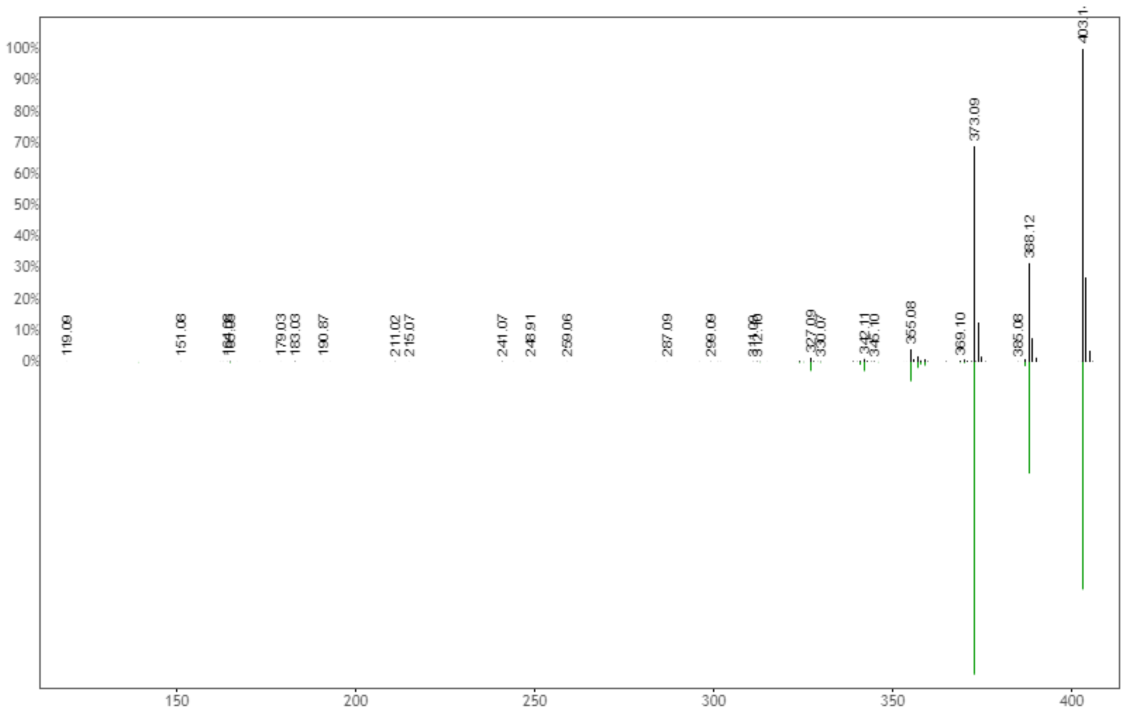

Figure S48. Mirror Match of GNPS to nobiletin in positive mode with Gold classification in Library Class of asymptomatic, symptomatic and healthy leaf sample. Green data are *m/z* values of GNPS Library and black are experimental data.

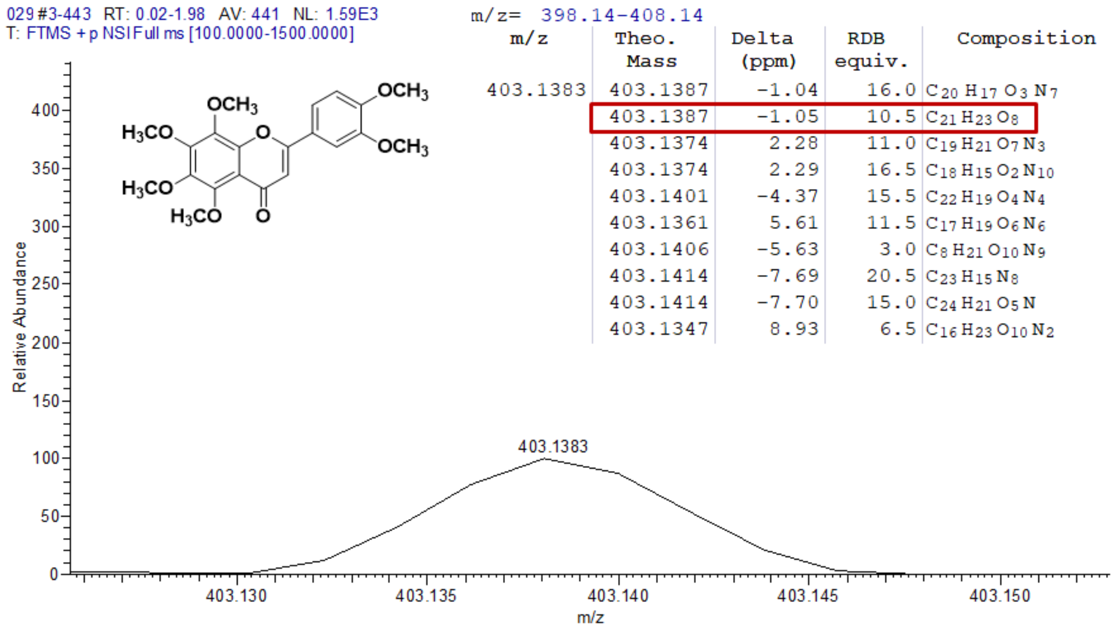

Figure S49. Mass spectrum - LC-MS in positive mode of leaf samples from *Citrus sinensis*. Assignment of *m/z* 403.1387 to nobiletin.

Nobiletin ($C_{21}H_{22}O_8$)

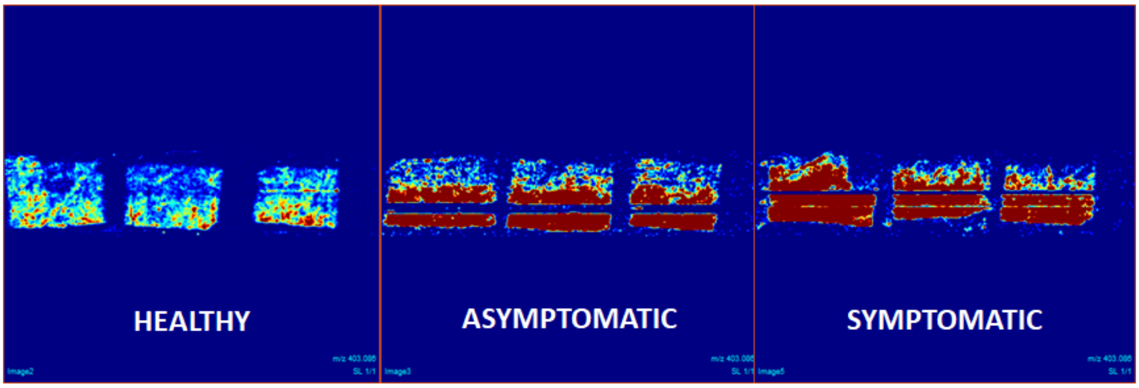

Figure S50. Image obtained by Mass Spectrometry Imaging (MSI) in positive mode of leaf samples from *Citrus sinensis* for nobiletin produced in different conditions.

Phenylalanine ($C_9H_{11}NO_2$)

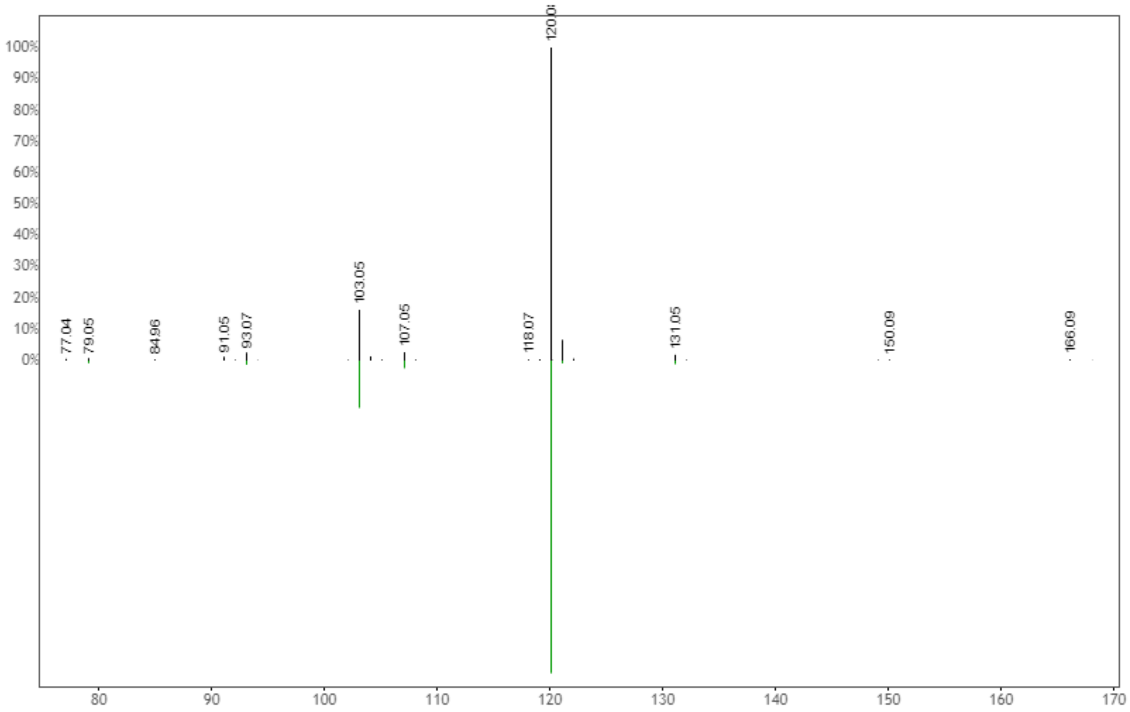

Figure S51. Mirror Match of GNPS to phenylalanine in positive mode with Gold classification in Library Class of asymptomatic sample. Green data are *m/z* values of GNPS Library and black are experimental data.

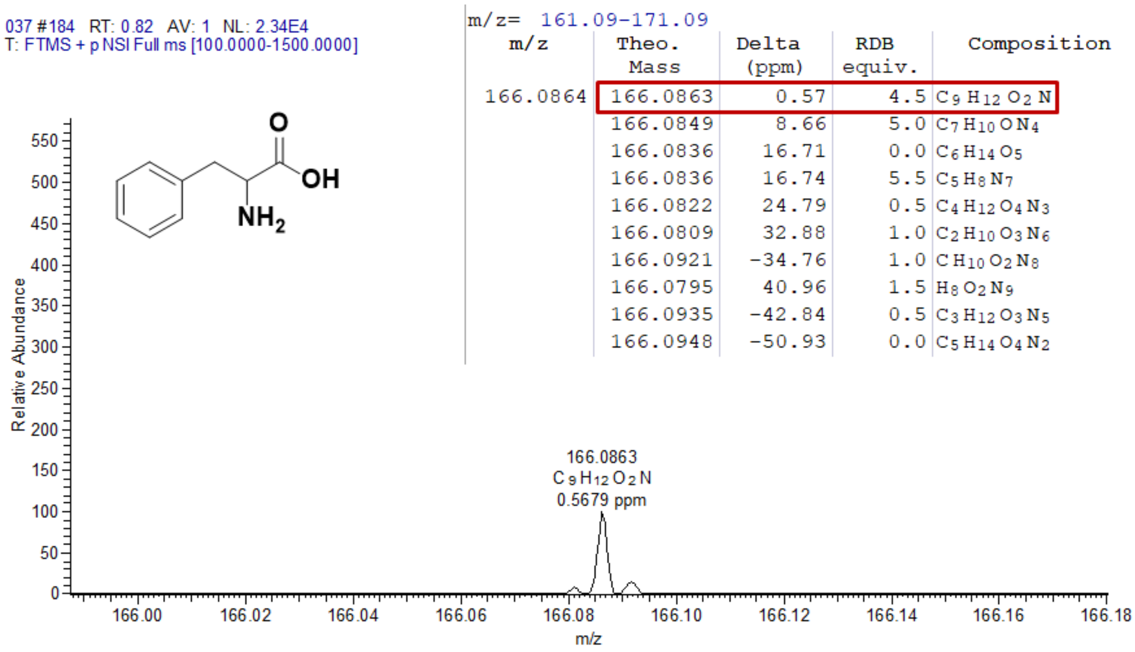

Figure S52. Mass spectrum - LC-MS in positive mode of leaf samples from *Citrus sinensis*. Assignment of *m/z* 188.0863 to phenylalanine.

Phenylalanine ($C_9H_{11}NO_2$)

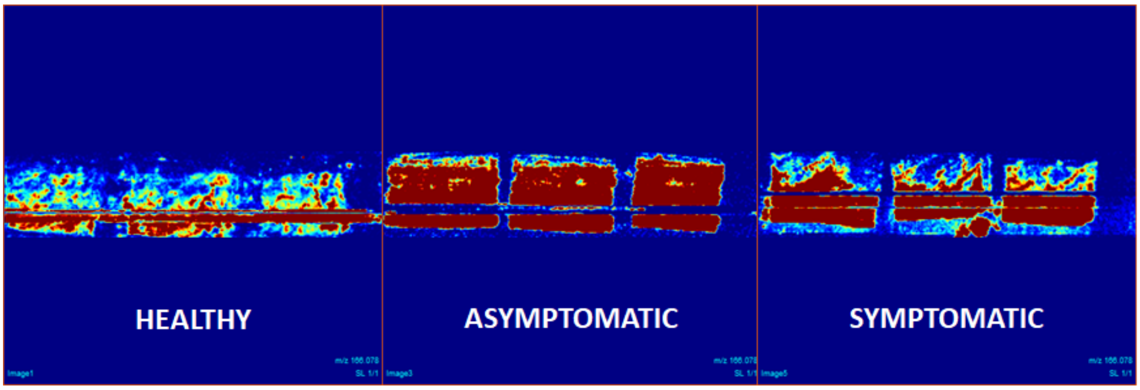

Figure S53. Image obtained by Mass Spectrometry Imaging (MSI) in positive mode of leaf samples from *Citrus sinensis* for phenylalanine produced in different conditions.

Pipecolic acid ($C_6H_{11}NO_2$)

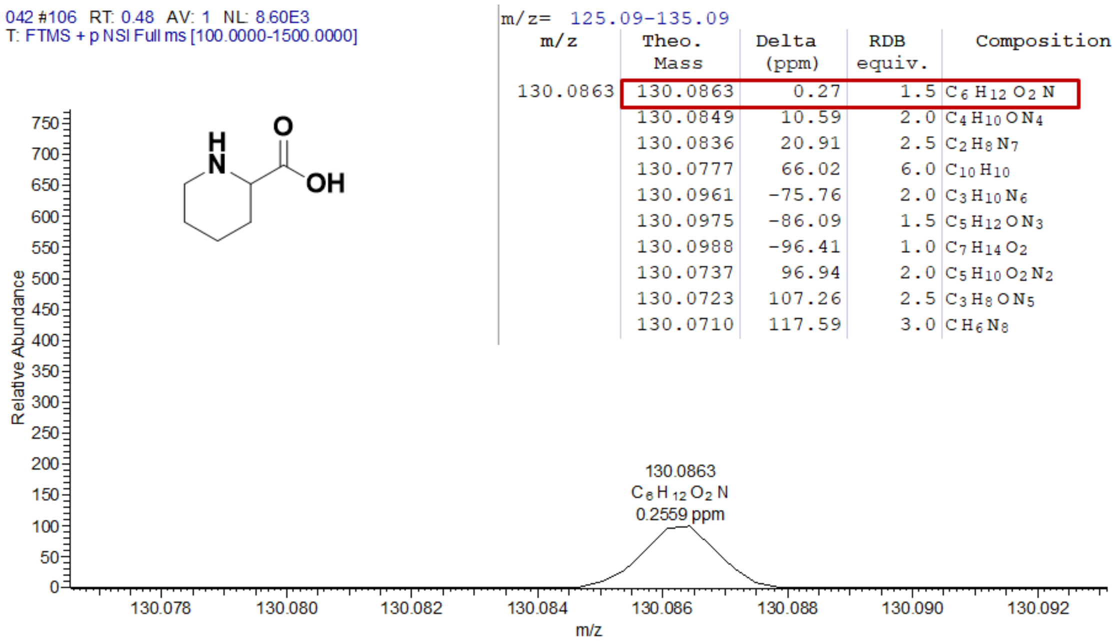

m/z	Theo. Mass	Delta (ppm)	RDB equiv.	Composition
130.0863	130.0863	0.27	1.5	$C_6H_{12}O_2N$
	130.0849	10.59	2.0	$C_4H_{10}ON_4$
	130.0836	20.91	2.5	$C_2H_8N_7$
	130.0777	66.02	6.0	$C_{10}H_{10}$
	130.0961	-75.76	2.0	$C_3H_{10}N_6$
	130.0975	-86.09	1.5	$C_5H_{12}ON_3$
	130.0988	-96.41	1.0	$C_7H_{14}O_2$
	130.0737	96.94	2.0	$C_5H_{10}O_2N_2$
	130.0723	107.26	2.5	$C_3H_8ON_5$
	130.0710	117.59	3.0	CH_6N_8

Figure S54. Mass spectrum - LC-MS in positive mode of leaf samples from *Citrus sinensis*. Assignment of *m/z* 130.0863 to pipecolic acid.

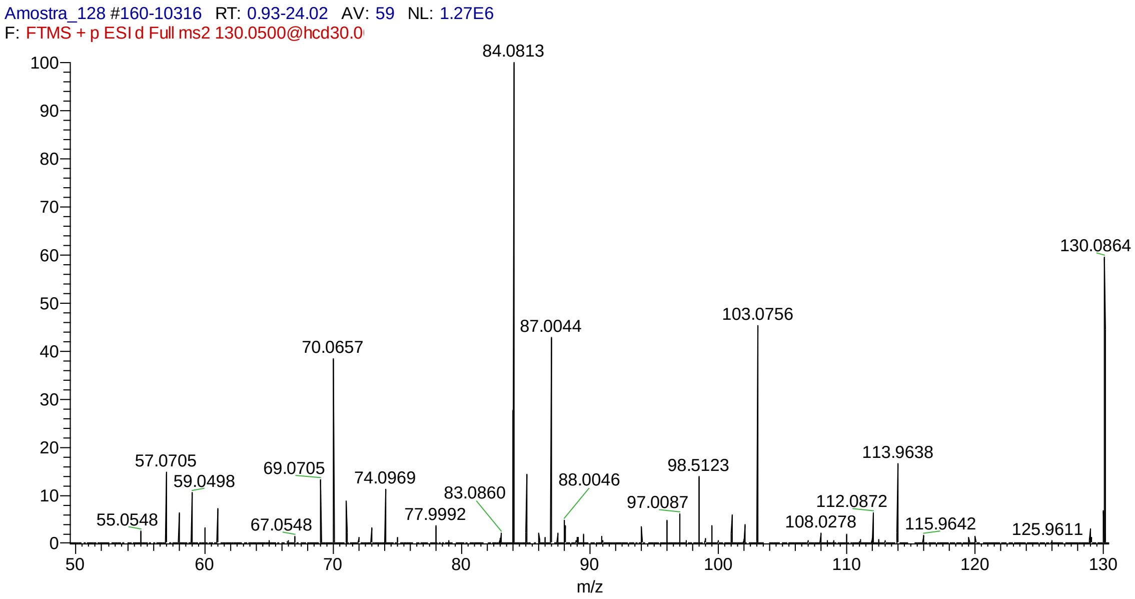

Figure S55. LC-MS/MS in positive mode (*m/z* 130.0500) of leaf samples from *Citrus sinensis*.

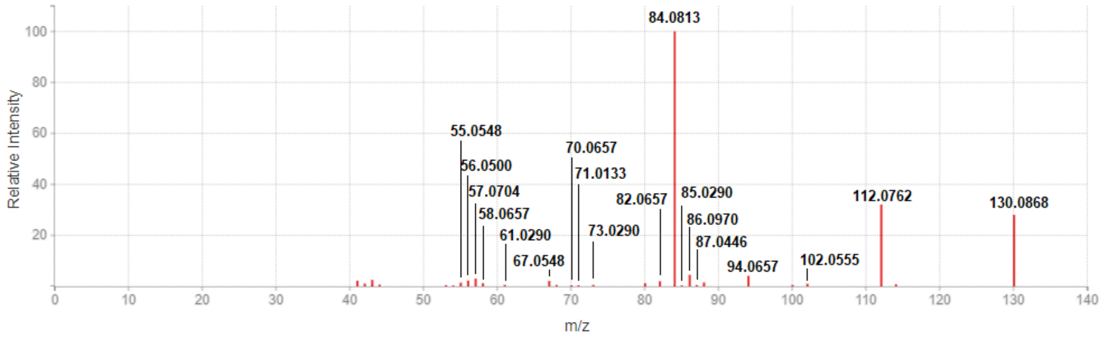

Figure S56. Predicted LC-MS/MS spectrum in positive mode of pipecolic acid available in Human Metabolome Database (http://www.hmdb.ca/spectra/ms_ms/20742).

Comparison between experimental *m/z* values and database to pipecolic acid in positive mode

**Database *m/z* (HMDB)**	**Experimental *m/z***	**Error (ppm)**	**Formula Xcalibur**
130.0868	130.0864	0.81	$C_6H_{12}NO_2$
112.0762	112.0759	2.05	$C_6H_{10}NO$
102.0555	102.0552	2.11	$C_4H_8NO_2$
94.0657	94.0656	4.93	C_6H_8N
87.0446	87.0445	4.53	$C_4H_7O_2$
86.0970	86.0968	4.58	$C_5H_{12}N$
85.0290	85.0288	4.87	$C_4H_5O_2$
84.0813	84.0813	5.88	$C_5H_{10}N$
82.0657	82.0656	5.41	C_5H_8N
73.0290	73.0289	6.35	$C_3H_5O_2$
71.0133	71.0134	9.35	$C_3H_3O_2$
70.0657	70.0657	8.77	C_4H_8N
67.0548	67.0548	9.14	C_5H_7
61.0290	61.0290	9.41	$C_2H_5O_2$
58.0657	58.0658	11.44	C_3H_8N
57.0704	57.0705	11.44	C_4H_9
55.0548	55.0548	11.32	C_4H_7

Pipecolic acid ($C_6H_{11}NO_2$)

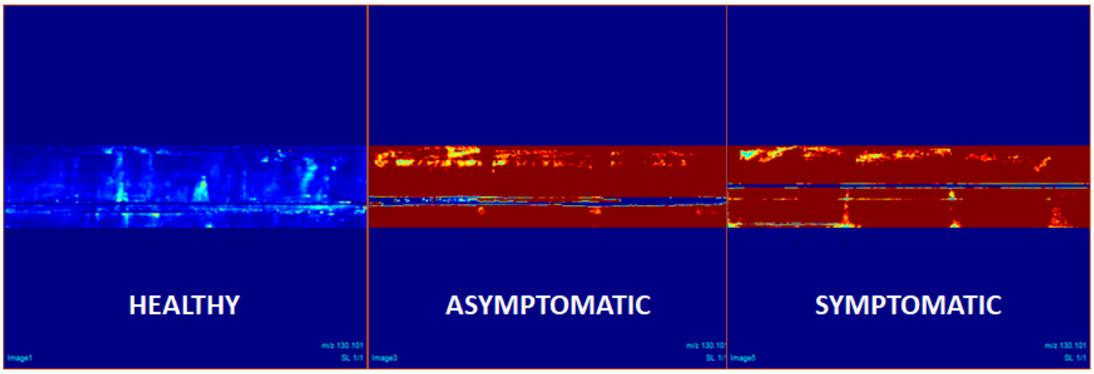

Figure S57. Image obtained by Mass Spectrometry Imaging (MSI) in positive mode of leaf samples from *Citrus sinensis* for pipecolic acid produced in different conditions.

Quinic acid ($C_7H_{12}O_6$)

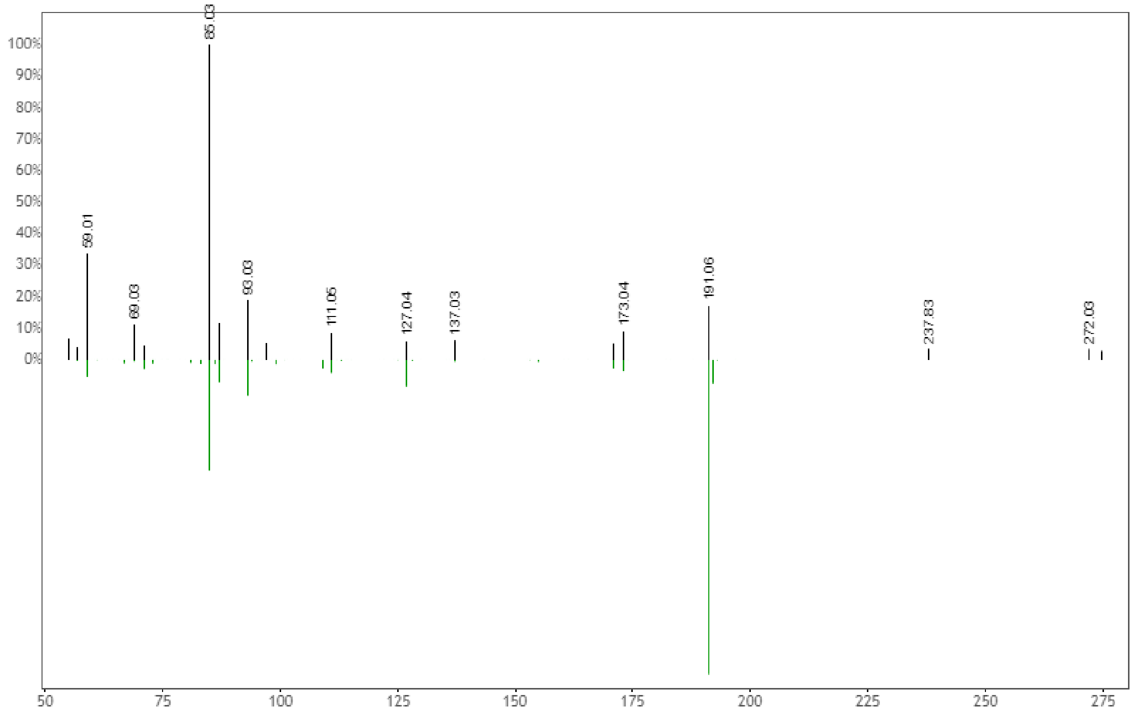

Figure S58. Mirror Match of GNPS to quinic acid in positive mode with Bronze classification in Library Class of asymptomatic sample. Green data are *m/z* values of GNPS Library and black are experimental data.

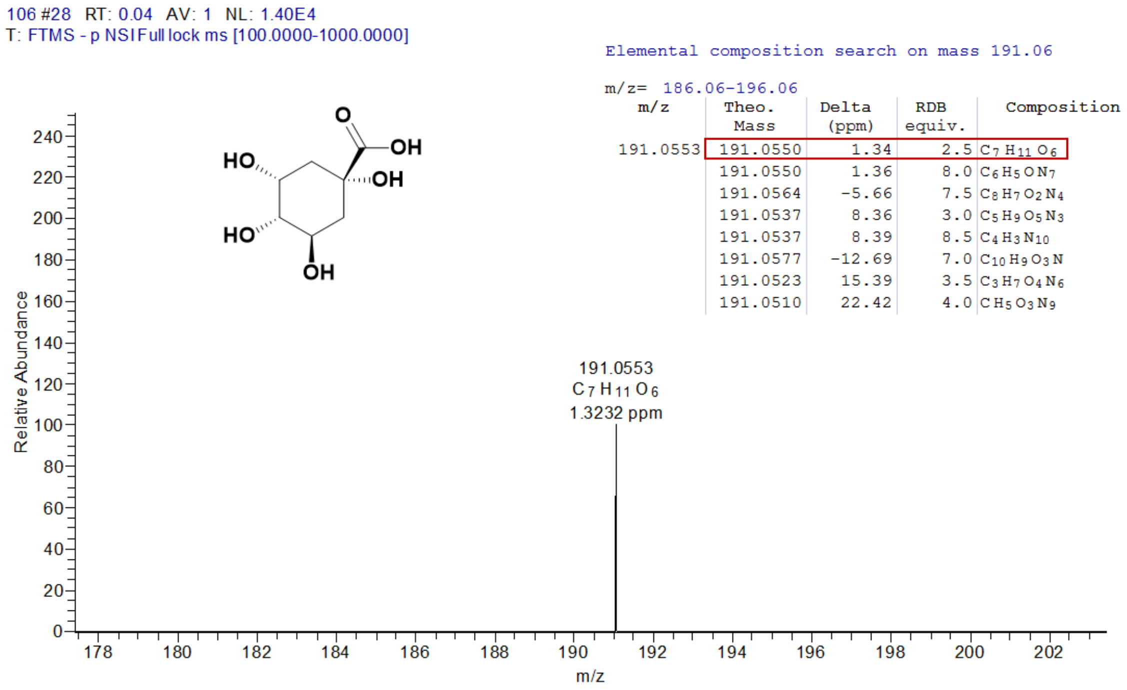

Figure S59. Mass spectrum - LC-MS in negative mode of leaf samples from *Citrus sinensis*. Assignment of *m/z* 191.0550 to quinic acid.

Quinic acid ($C_7H_{12}O_6$)

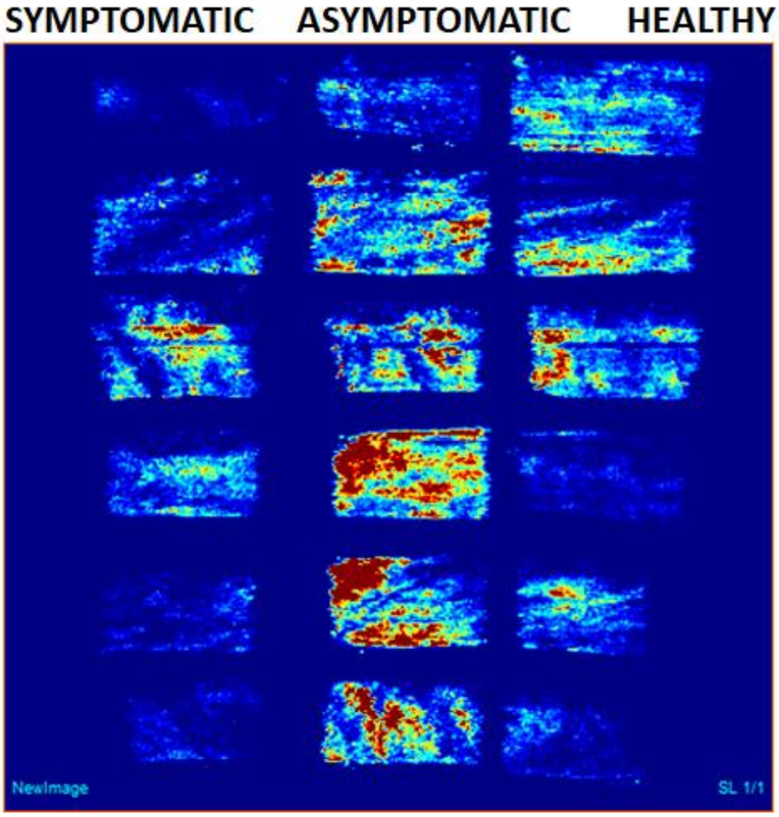

Figure S60. Image obtained by Mass Spectrometry Imaging (MSI) in negative mode of leaf samples from *Citrus sinensis* for quinic acid produced in different conditions.

Sucrose ($C_{12}H_{22}O_{11}$)

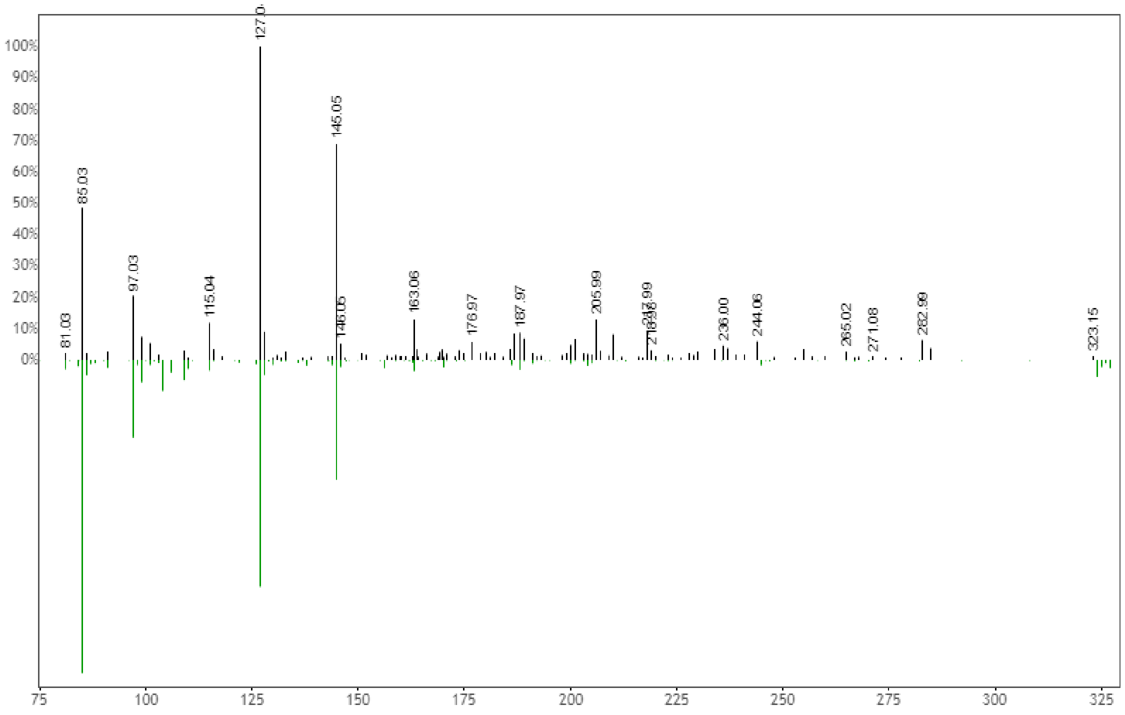

Figure S61. Mirror Match of GNPS to sucrose in positive mode with Bronze classification in Library of healthy sample Class. Green data are *m/z* values of GNPS Library and black are experimental data.

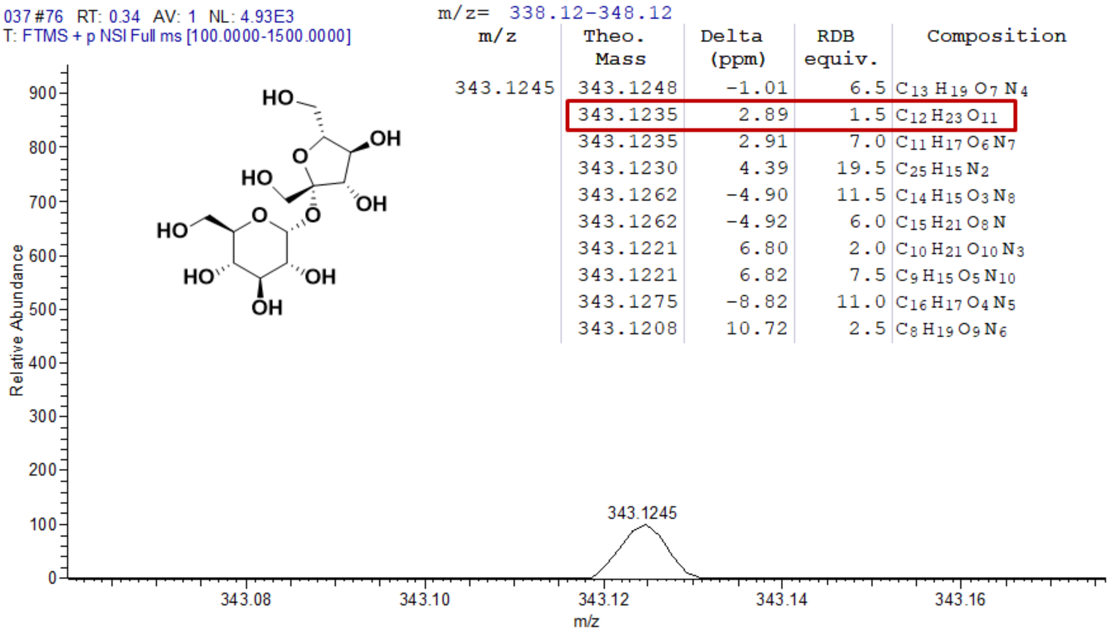

Figure S62. Mass spectrum - LC-MS in positive mode of leaf samples from *Citrus sinensis*. Assignment of *m/z* 343.1235 to sucrose.

Sucrose ($C_{12}H_{22}O_{11}$)

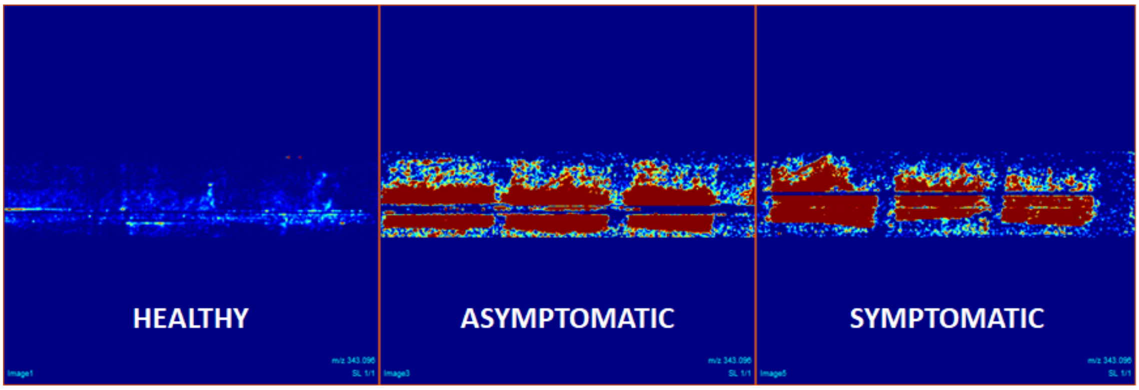

Figure S63. Image obtained by Mass Spectrometry Imaging (MSI) in positive mode of leaf samples from *Citrus sinensis* for sucrose produced in different conditions.

Synephrine ($C_9H_{13}NO_2$)

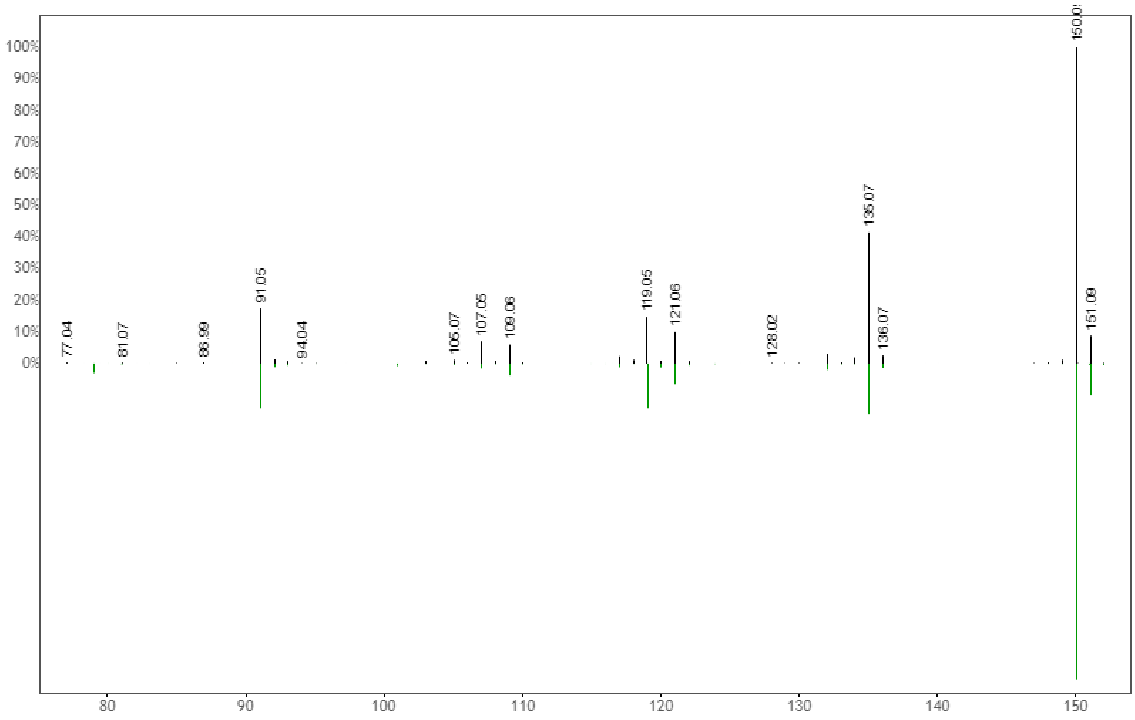

Figure S64. Mirror Match of GNPS to synephrine in positive mode with Bronze classification in Library Class of asymptomatic, symptomatic and healthy sample. Green data are *m/z* values of GNPS Library and black are experimental data.

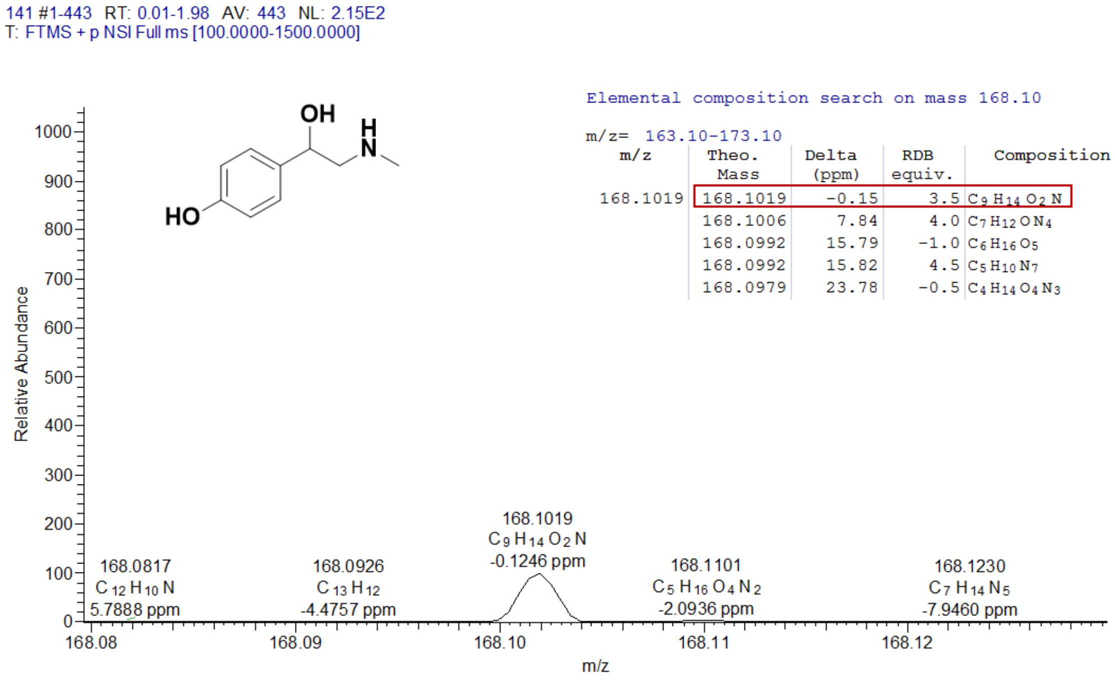

Figure S65. Mass spectrum - LC-MS in positive mode of leaf samples from *Citrus sinensis*. Assignment of *m/z* 168.1019 to synephrine.

Synephrine ($C_9H_{13}NO_2$)

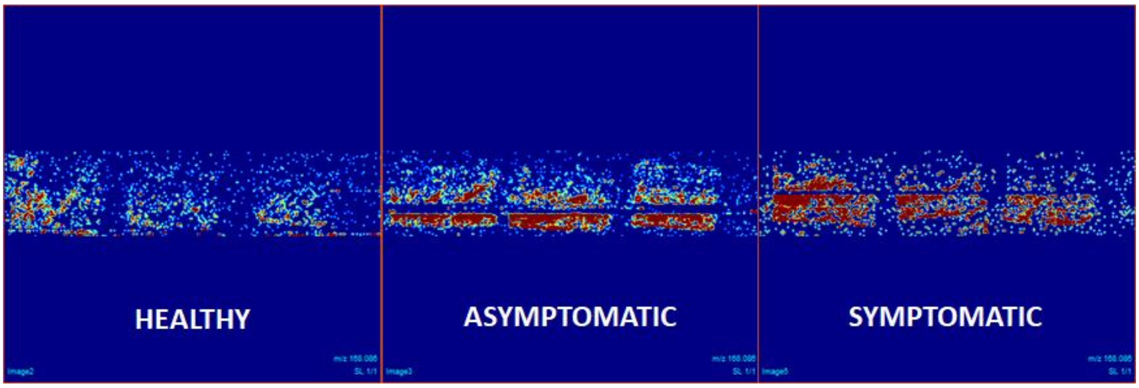

Figure S66. Image obtained by Mass Spectrometry Imaging (MSI) in positive mode of leaf samples from *Citrus sinensis* for synephrine produced in different conditions.

Tangeretin ($C_{20}H_{20}O_7$)

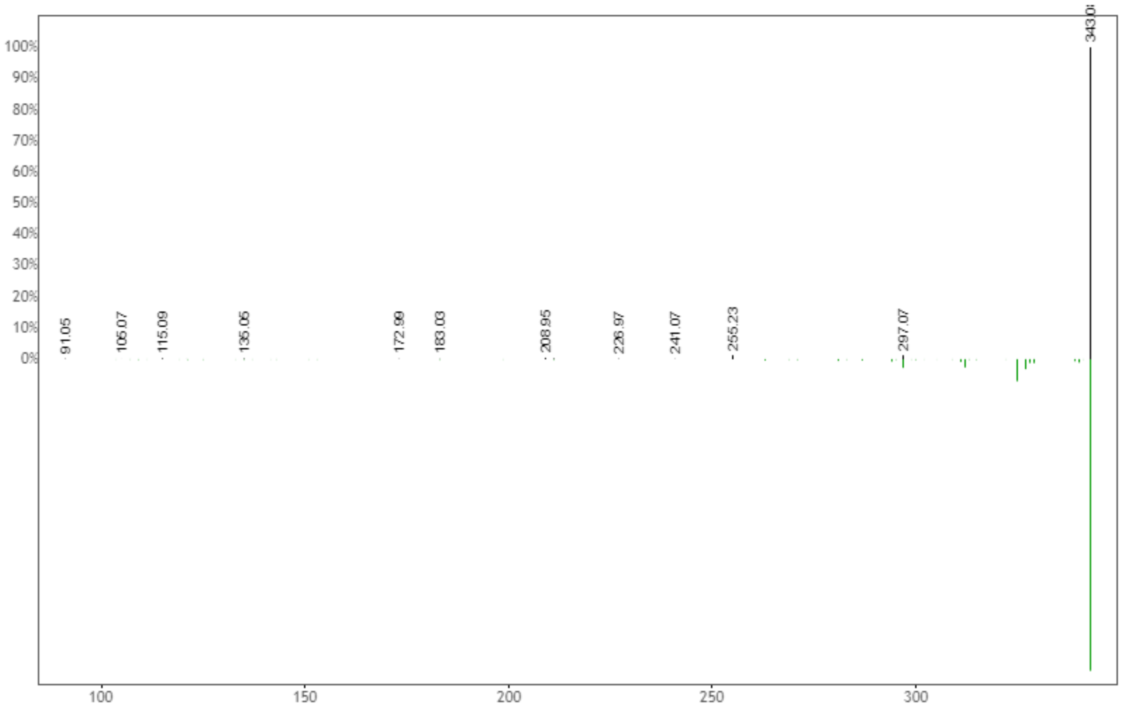

Figure S67. Mirror Match of GNPS to tangeretin in positive mode with Bronze classification in Library Class of asymptomatic, symptomatic and healthy leaf sample. Green data are *m/z* values of GNPS Library and black are experimental data.

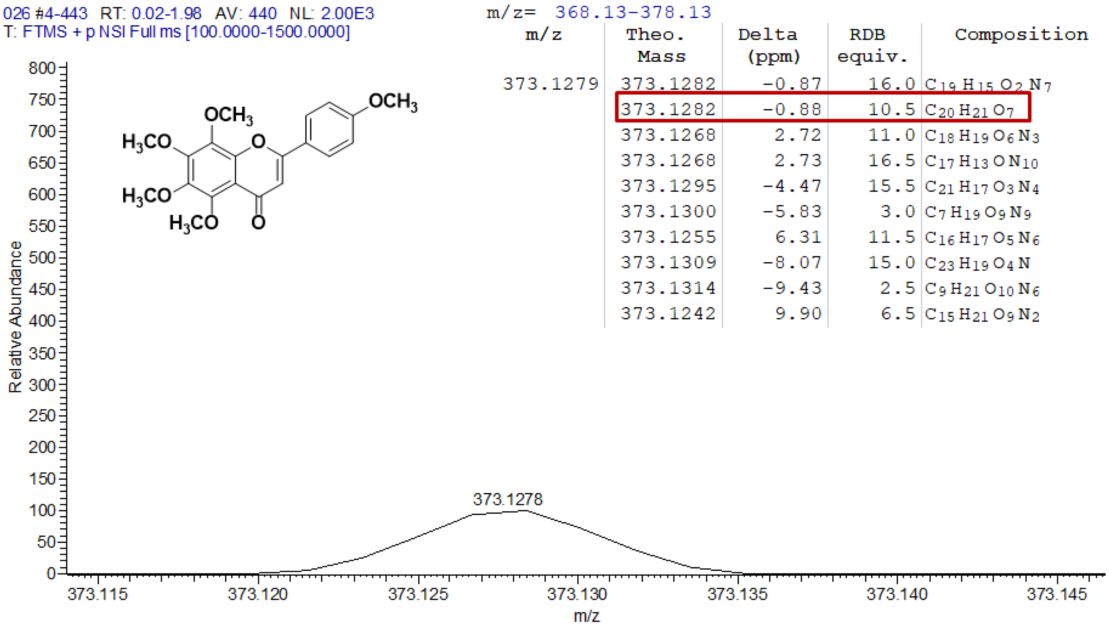

Figure S68. Mass spectrum - LC-MS in positive mode of leaf samples from *Citrus sinensis*. Assignment of *m/z* 373.1282 to tangeretin.

Tangeretin ($C_{20}H_{20}O_7$)

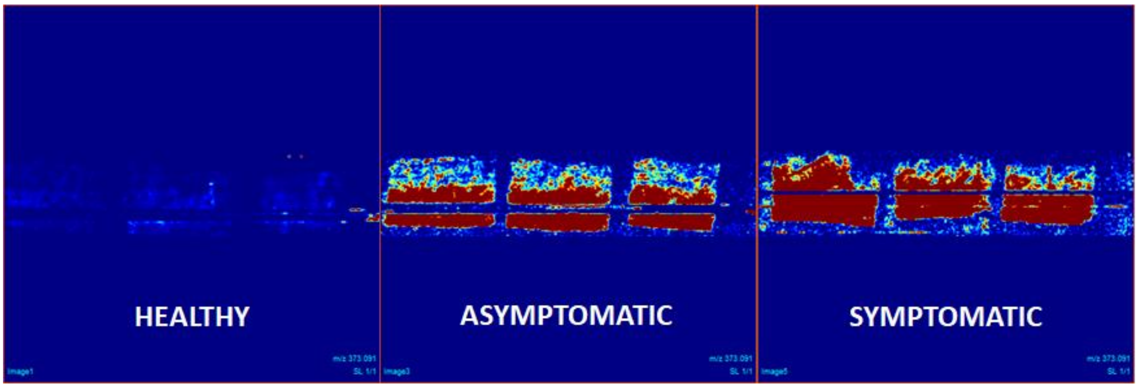

Figure S69. Image obtained by Mass Spectrometry Imaging (MSI) in positive mode of leaf samples from *Citrus sinensis* for tangeretin produced in different conditions.

4',5,6,7-Tetramethoxyflavone ($C_{19}H_{18}O_6$)

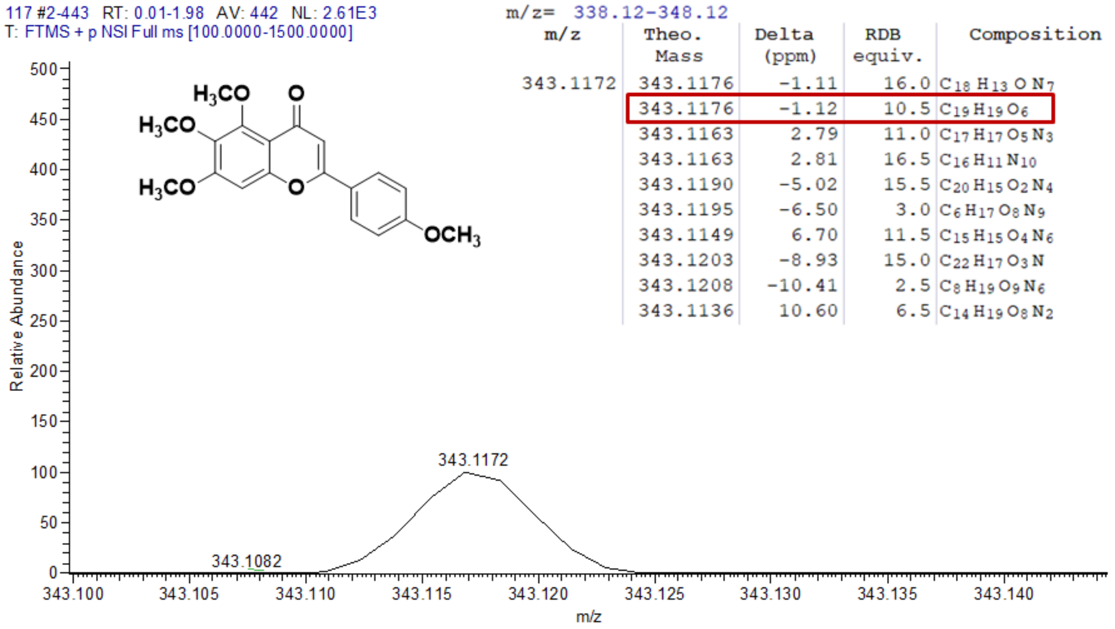

m/z	Theo. Mass	Delta (ppm)	RDB equiv.	Composition
343.1172	343.1176	-1.11	16.0	$C_{18}H_{13}ON_7$
	343.1176	-1.12	10.5	$C_{19}H_{19}O_6$
	343.1163	2.79	11.0	$C_{17}H_{17}O_5N_3$
	343.1163	2.81	16.5	$C_{16}H_{11}N_{10}$
	343.1190	-5.02	15.5	$C_{20}H_{15}O_2N_4$
	343.1195	-6.50	3.0	$C_6H_{17}O_8N_9$
	343.1149	6.70	11.5	$C_{15}H_{15}O_4N_6$
	343.1203	-8.93	15.0	$C_{22}H_{17}O_3N$
	343.1208	-10.41	2.5	$C_8H_{19}O_9N_6$
	343.1136	10.60	6.5	$C_{14}H_{19}O_8N_2$

Figure S70. Mass spectrum - LC-MS in positive mode of leaf samples from *Citrus sinensis*. Assignment of *m/z* 343.1176 to 4',5,6,7-tetramethoxyflavone.

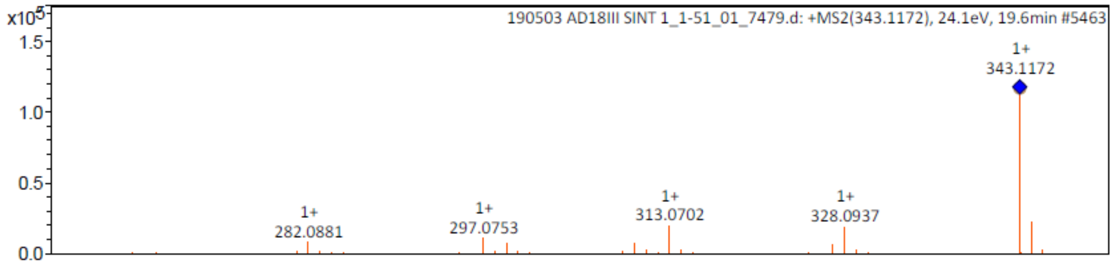

Figure S71. LC-MS/MS in positive mode (*m/z* 343.1172) of leaf samples from *Citrus sinensis*.

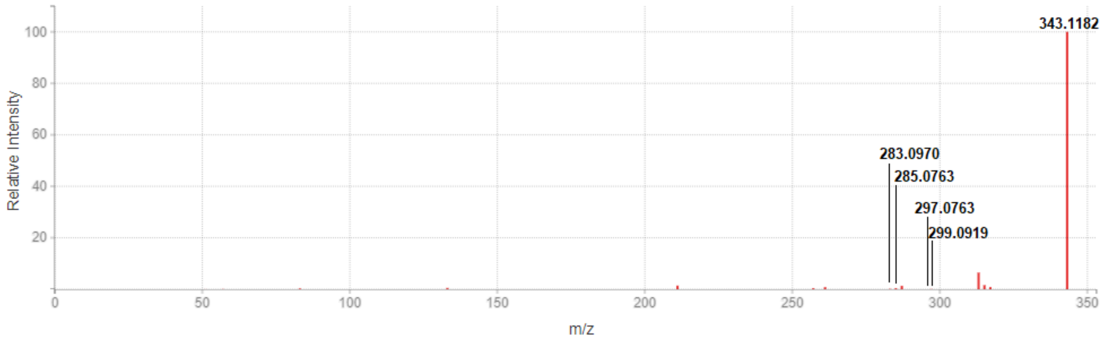

Figure S72. Predicted LC-MS/MS spectrum in positive mode of 4',5,6,7-tetramethoxyflavone available in Human Metabolome Database (http://www.hmdb.ca/spectra/ms_ms/73348).

Comparison between experimental *m/z* values of LC-MS/MS and database to 4',5,6,7-tetramethoxyflavone in positive mode

Database *m/z* (HMDB)	Experimental *m/z*	Error (ppm)	Formula Xcalibur
343.1182	343.1172	1.1	$C_{19}H_{19}O_6$
299.0919	299.0908	1.9	$C_{17}H_{15}O_5$
297.0763	297.0753	1.5	$C_{17}H_{13}O_5$
285.0763	285.0757	5.7	$C_{16}H_{13}O_5$
283.0970	283.0917	---	$C_{17}H_{15}O_4$

4',5,6,7-Tetramethoxyflavone ($C_{19}H_{18}O_6$)

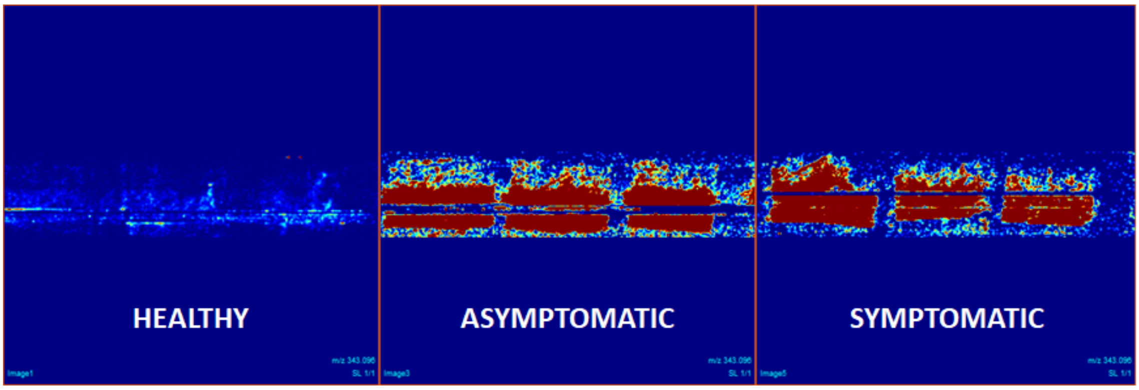

Figure S73. Image obtained by Mass Spectrometry Imaging (MSI) in positive mode of leaf samples from *Citrus sinensis* for 4',5,6,7-Tetramethoxyflavone produced in different conditions.

Tryptophan ($C_{11}H_{12}N_2O_2$)

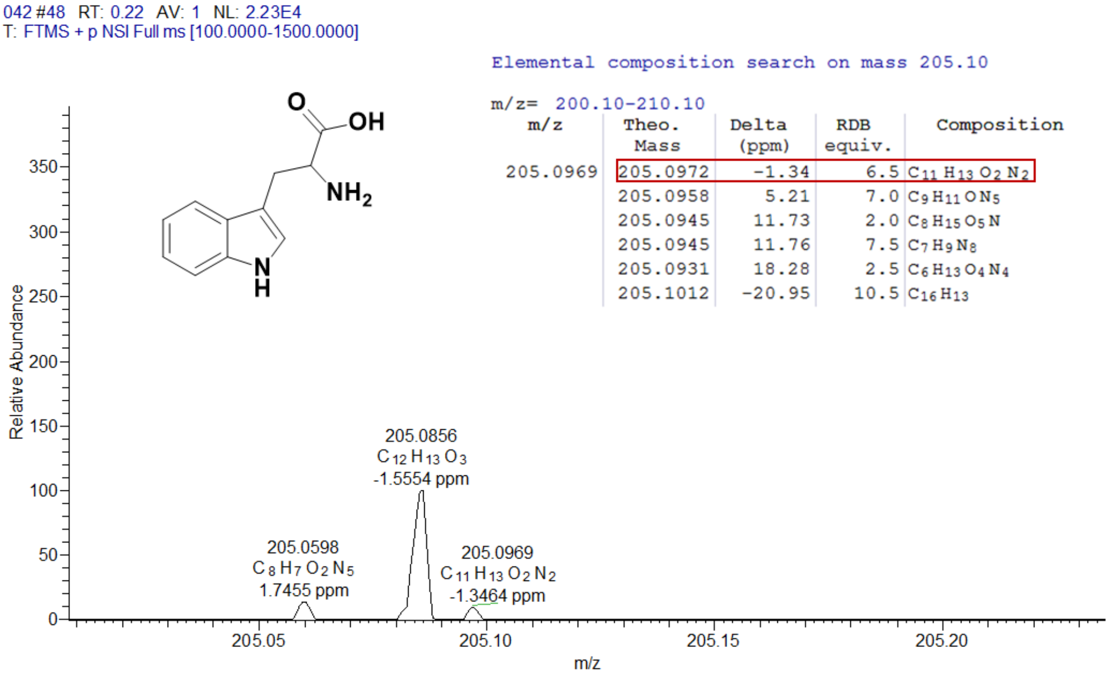

Figure S74. Mass spectrum - LC-MS in positive mode of leaf samples from *Citrus sinensis*. Assignment of *m/z* 205.0972 to tryptophan.

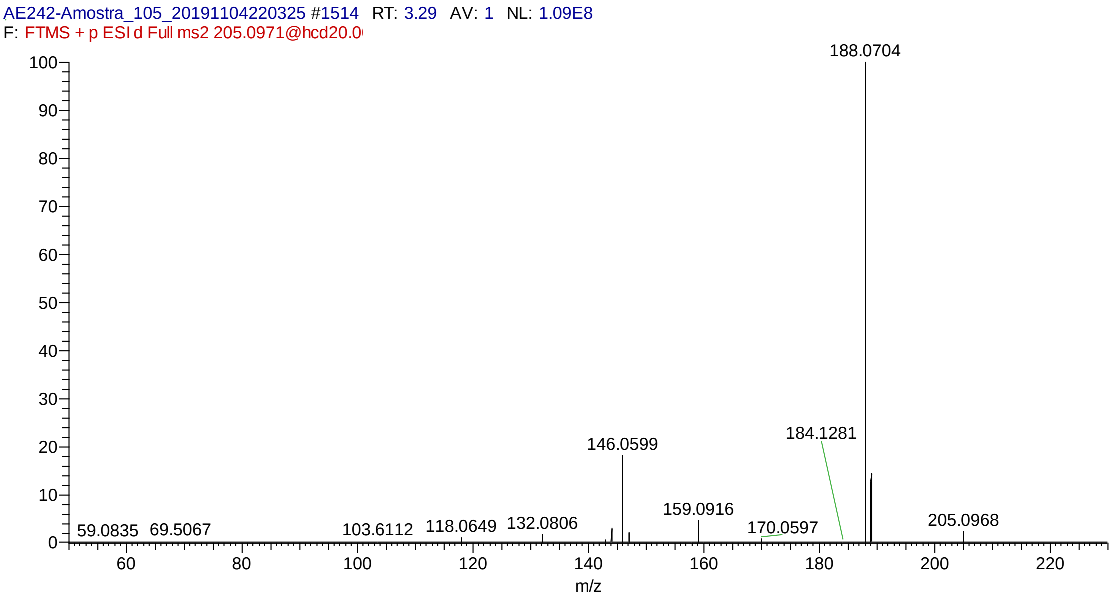

Figure S75. LC-MS/MS in positive mode (*m/z* 205.0971) of leaf samples from *Citrus sinensis*.

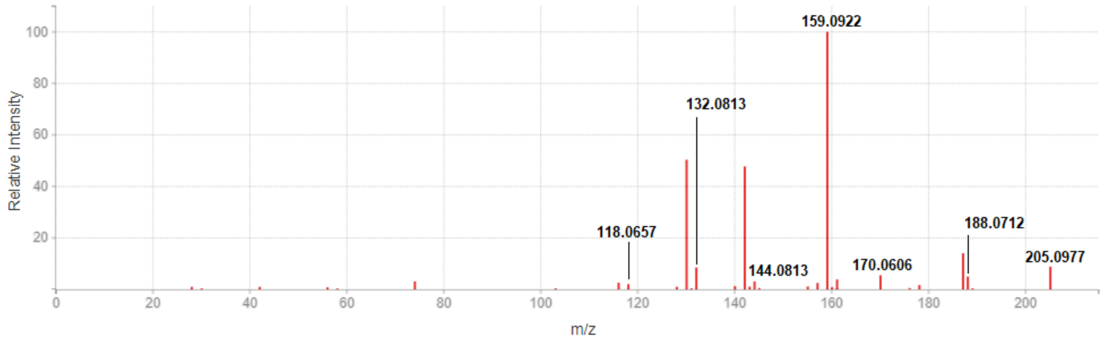

Figure S76. Predicted LC-MS/MS spectrum in positive mode of tryptophan available in Human Metabolome Database (http://www.hmdb.ca/spectra/ms_ms/21129).

Comparison between experimental *m/z* values of LC-MS/MS and database to tryptophan in positive mode

Database *m/z* (HMDB) predicted spectrum	Database *m/z* (GNPS) deposited spectrum	Experimental *m/z*	Error (ppm)	Formula Xcalibur
205.0977	---	205.0970	-0.61	$C_{11}H_{13}N_2O_2$
188.0712	188.07	188.0704	-0.98	$C_{11}H_{10}NO_2$
170.0606	170.06	170.0603	1.29	$C_{11}H_8NO$
159.0922	159.09	159.0915	-0.85	$C_{10}H_{11}N_2$
144.0813	144.08	144.0808	0.44	$C_{10}H_{10}N$
132.0813	132.08	132.0808	0.49	$C_9H_{10}N$
118.0657	118.07	118.0656	4.10	C_8H_8N

Tryptophan ($C_{11}H_{12}N_2O_2$)

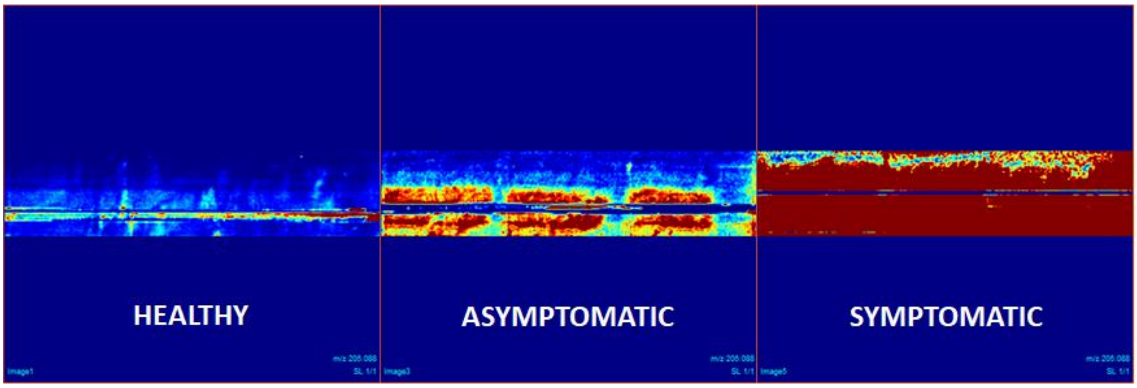

Figure S77. Image obtained by Mass Spectrometry Imaging (MSI) in positive mode of leaf samples from *Citrus sinensis* for tryptophan produced in different conditions.

Tyrosine ($C_9H_{11}NO_3$)

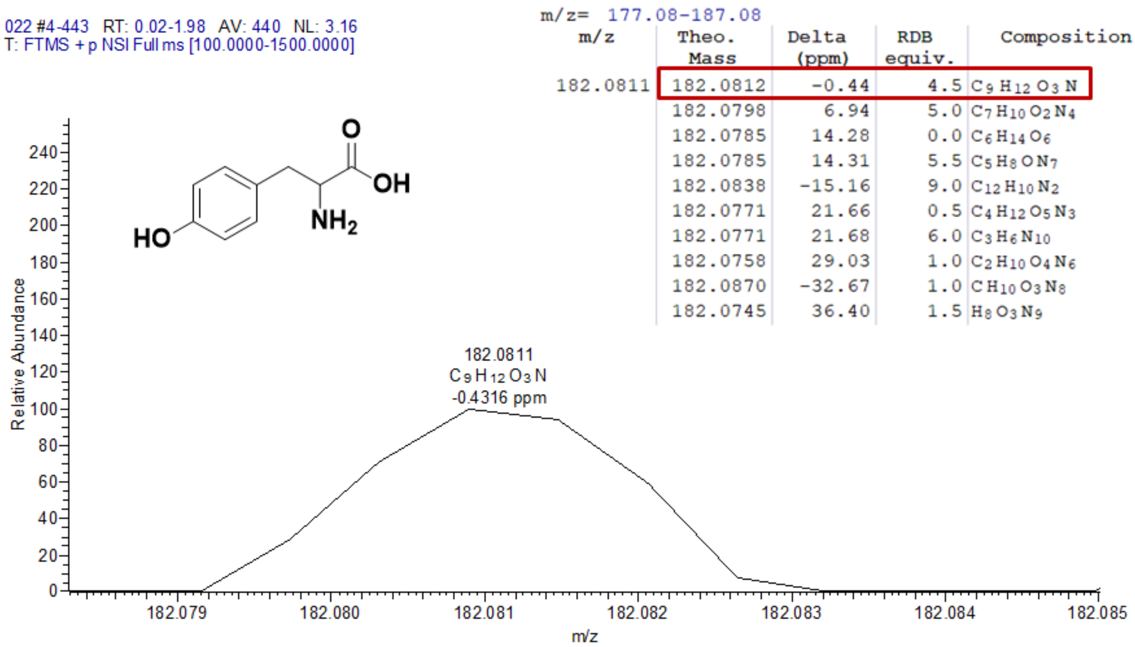

m/z= 177.08-187.08

m/z	Theo. Mass	Delta (ppm)	RDB equiv.	Composition
182.0811	182.0812	-0.44	4.5	$C_9H_{12}O_3N$
	182.0798	6.94	5.0	$C_7H_{10}O_2N_4$
	182.0785	14.28	0.0	$C_6H_{14}O_6$
	182.0785	14.31	5.5	$C_5H_8ON_7$
	182.0838	-15.16	9.0	$C_{12}H_{10}N_2$
	182.0771	21.66	0.5	$C_4H_{12}O_5N_3$
	182.0771	21.68	6.0	$C_3H_6N_{10}$
	182.0758	29.03	1.0	$C_2H_{10}O_4N_6$
	182.0870	-32.67	1.0	$CH_{10}O_3N_8$
	182.0745	36.40	1.5	$H_8O_3N_9$

Figure S78. Mass spectrum - LC-MS in positive mode of leaf samples from *Citrus sinensis*. Assignment of *m/z* 182.0812 to tyrosine.

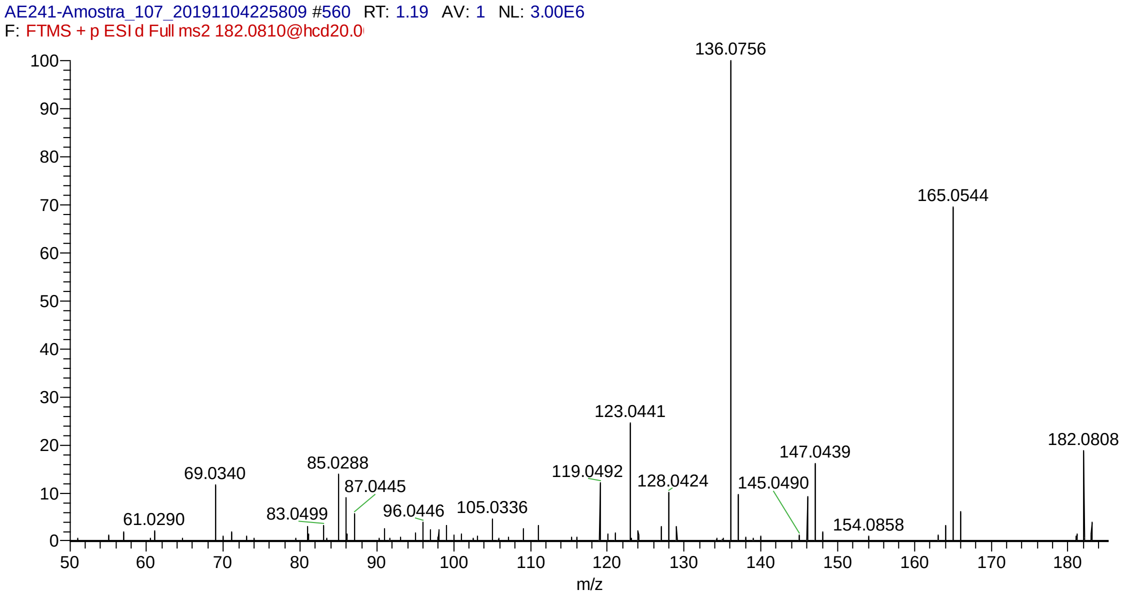

Figure S79. LC-MS/MS in positive mode (*m/z* 182.0810) of leaf samples from *Citrus sinensis*.

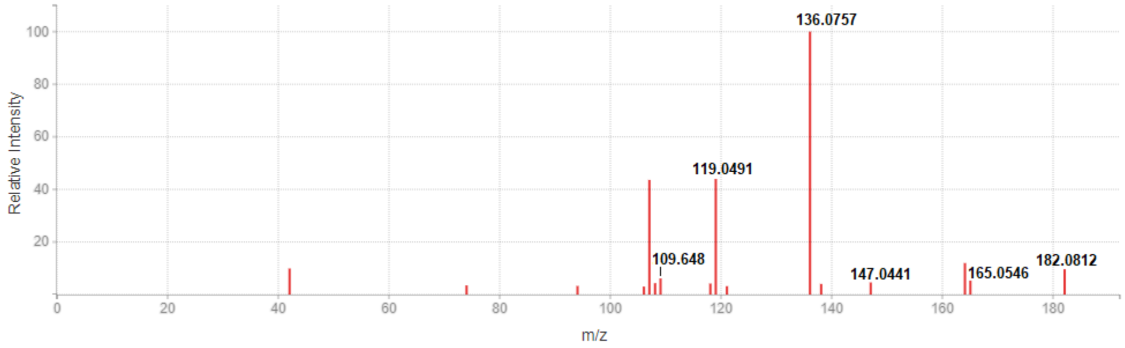

Figure S80. Predicted LC-MS/MS spectrum in positive mode of tyrosine available in Human Metabolome Database (http://www.hmdb.ca/spectra/ms_ms/296222).

Comparison between experimental *m/z* values of LC-MS/MS and database to tyrosine in positive mode

Database *m/z* (HMDB) predicted spectrum	Database *m/z* (GNPS) deposited spectrum	Experimental *m/z* (full scan)	Error (ppm)	Formula Xcalibur
182.0812	182.08	182.0808	-1.87	$C_9H_{12}NO_3$
165.0546	165.05	165.0544	-1.34	$C_9H_9O_3$
147.0441	147.04	147.0439	-1.20	$C_9H_7O_2$
136.0757	136.08	136.0756	-0.96	$C_8H_{10}NO$
119.0491	119.05	119.0492	0.41	C_8H_7O
109.0648	---	109.0651	2.74	C_7H_9O

Tyrosine ($C_9H_{11}NO_3$)

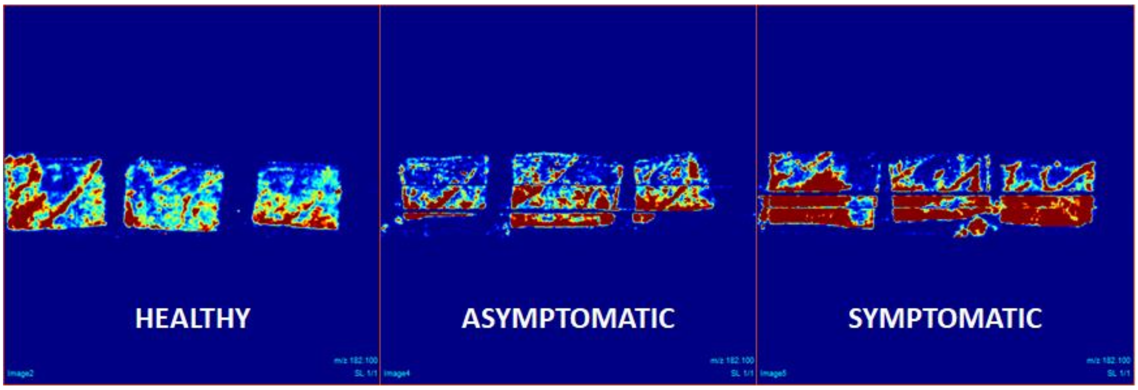

Figure S81. Image obtained by Mass Spectrometry Imaging (MSI) in positive mode of leaf samples from *Citrus sinensis* for tyrosine produced in different conditions.

Valine ($C_5H_{11}NO_2$)

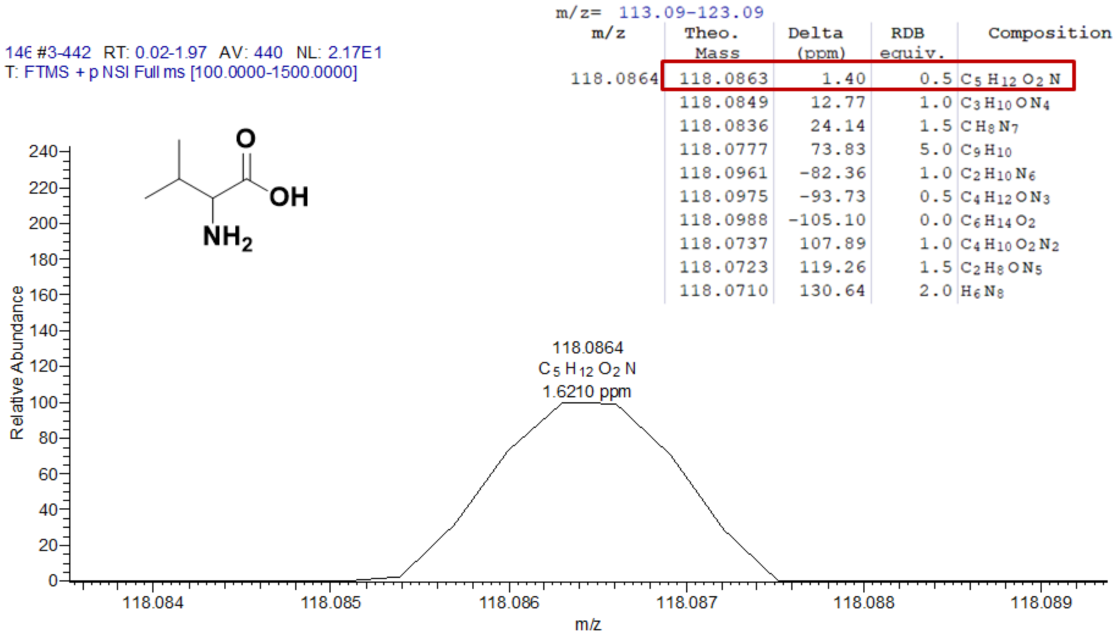

m/z= 113.09-123.09

m/z	Theo. Mass	Delta (ppm)	RDB equiv.	Composition
118.0864	118.0863	1.40	0.5	$C_5H_{12}O_2N$
	118.0849	12.77	1.0	$C_3H_{10}ON_4$
	118.0836	24.14	1.5	CH_8N_7
	118.0777	73.83	5.0	C_9H_{10}
	118.0961	-82.36	1.0	$C_2H_{10}N_6$
	118.0975	-93.73	0.5	$C_4H_{12}ON_3$
	118.0988	-105.10	0.0	$C_6H_{14}O_2$
	118.0737	107.89	1.0	$C_4H_{10}O_2N_2$
	118.0723	119.26	1.5	$C_2H_8ON_5$
	118.0710	130.64	2.0	H_6N_8

Figure S82. Mass spectrum - LC-MS in positive mode of leaf samples from *Citrus sinensis*. Assignment of *m/z* 118.0863 to valine.

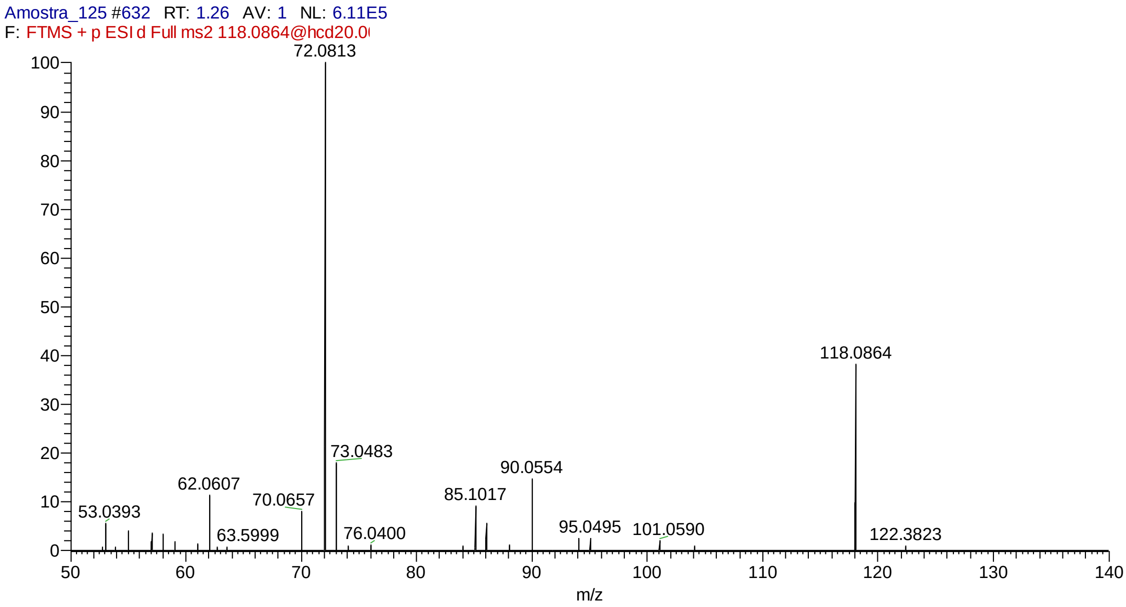

Figure S83. LC-MS/MS in positive mode (*m/z* 118.0864) of leaf samples from *Citrus sinensis*.

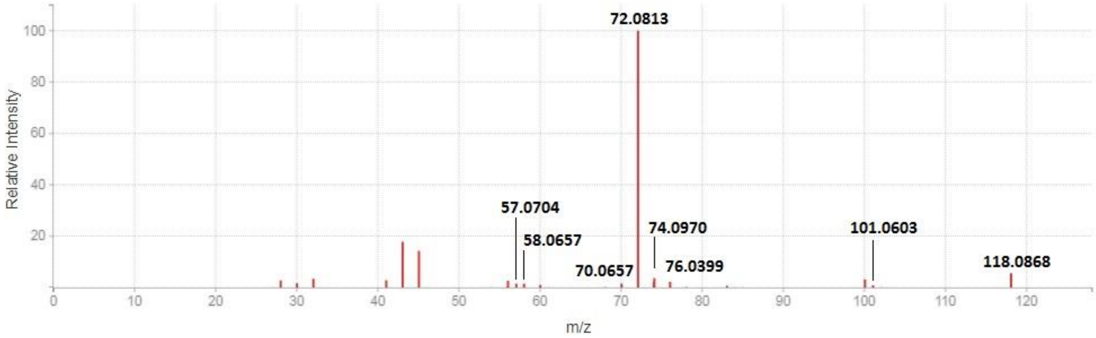

Figure S84. Predicted LC-MS/MS spectrum in positive mode of valine available in Human Metabolome Database (https://hmdb.ca/spectra/ms_ms/178807).

Comparison between experimental *m/z* values of LC-MS/MS and database to valine in positive mode

Database *m/z* (HMDB) predicted spectrum	Experimental *m/z* (full scan)	Error (ppm)	Formula Xcalibur
118.0868	118.0863	1.31	$C_5H_{12}NO_2$
101.0603	101.0597	-6.69	$C_5H_9O_2$
76.0399	76.0393	8.61	$C_2H_6NO_2$
74.0970	74.0964	3.97	$C_4H_{12}N$
72.0813	72.0813	7.83	$C_4H_{10}N$
70.0657	70.0651	8.05	C_4H_8N
58.0657	58.0651	11.61	C_4H_9
57.0704	57.0699	10.04	C_3H_8N

Valine ($C_5H_{11}NO_2$)

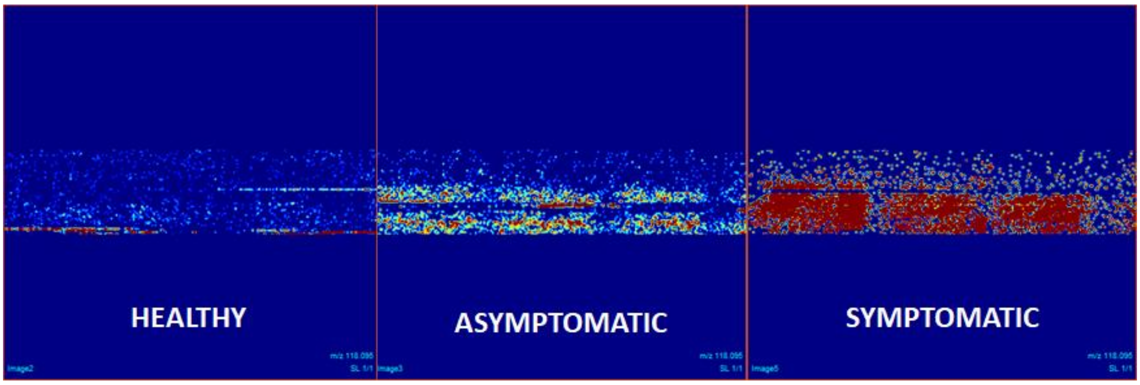

Figure S85. Image obtained by Mass Spectrometry Imaging (MSI) in positive mode of leaf samples from *Citrus sinensis* for valine produced in different conditions.

Accumulation of metabolites in healthy metabolic profile

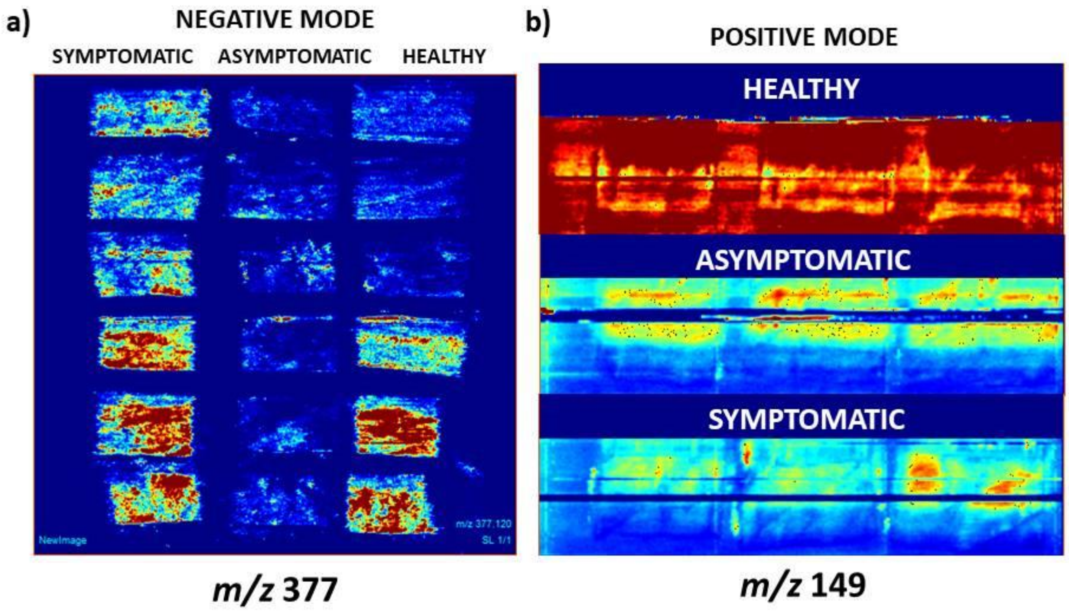

Figure S86. Images obtained by Mass Spectrometry Imaging (MSI) of leaf samples from *Citrus sinensis* in **a)** negative mode for the ion at *m/z* 377; **b)** positive mode for the ion at *m/z* 149. The compounds related to these images were not identified; however, they show that in our analyses there are examples of accumulation of compounds in the healthy metabolic profile.
